# Supplementary material for: Full eradication of pre‐clinical human papilloma virus‐induced tumors by a lentiviral vaccine
Source: EMBO Mol Med. 2023 Sep 7;15(10):e17723. doi: 10.15252/emmm.202317723 (PMC10565635; doi:10.15252/emmm.202317723)
Supplement: Supplementary file 1 — Appendix S1 [file EMMM-15-e17723-s002.pdf]

Appendix

Supplemental Data

Full Eradication of Pre-clinical Human PapillomaVirus-Induced Tumors by a Lentiviral Vaccine

Table of Contents

Appendix Figure S1. HPV-specific T-splenocyte responses in tumor-free or tumor-bearing mice.

Appendix Figure S2. Decreased proportions of intra-tumoral Treg and unchanged proportions of intra-tumoral B cells in Lenti-HPV-07-vaccinated mice.

Appendix Figure S3. Anti-PD1 treatment does not synergize with suboptimal dose of Lenti-HPV-07 on very large tumors.

Appendix Figure S4. Map of the plasmid encoding various HPV-07 poly-antigenic fusion proteins.

Appendix Table S1. Sequences of primers used in qRT-PCR.

Amino acid sequences of the detoxified HPV antigens.

Amino acid sequences of HPV poly-antigens.

Nucleic sequences of HPV poly-antigens.

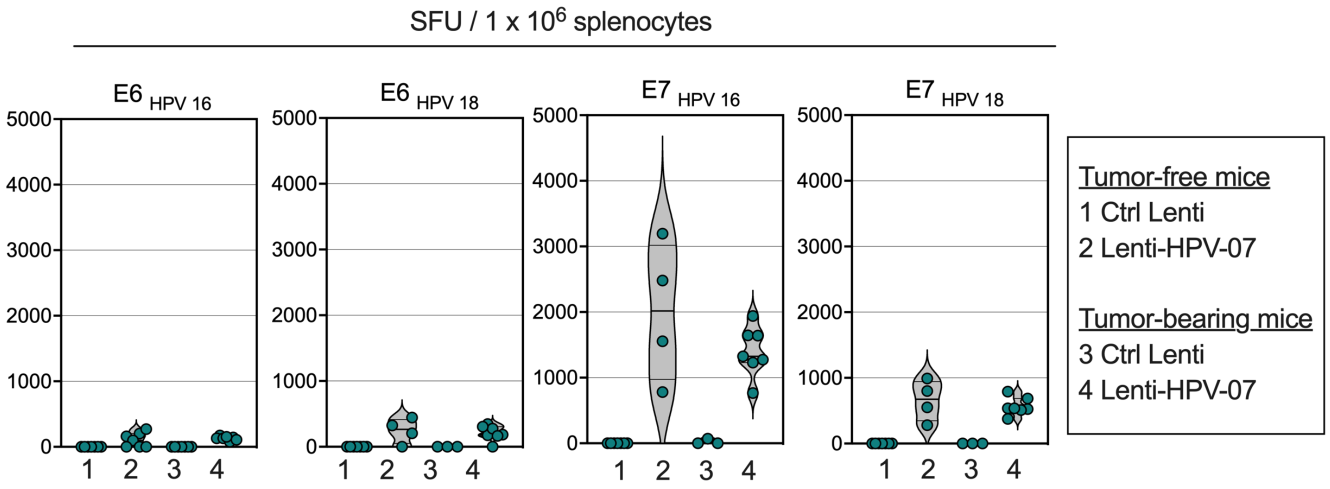

Appendix Figure S1. HPV-specific T-splenocyte responses in tumor-free or tumor-bearing mice. Tumor-free or tumor engrafted C57BL/6 mice (*n* = 3-7/group) were immunized i.m. with Ctrl Lenti or Lenti-HPV-07. On day 14 post-vaccination, T-splenocyte responses were assessed by IFN-γ ELISPOT, as detailed in Figure 2A.

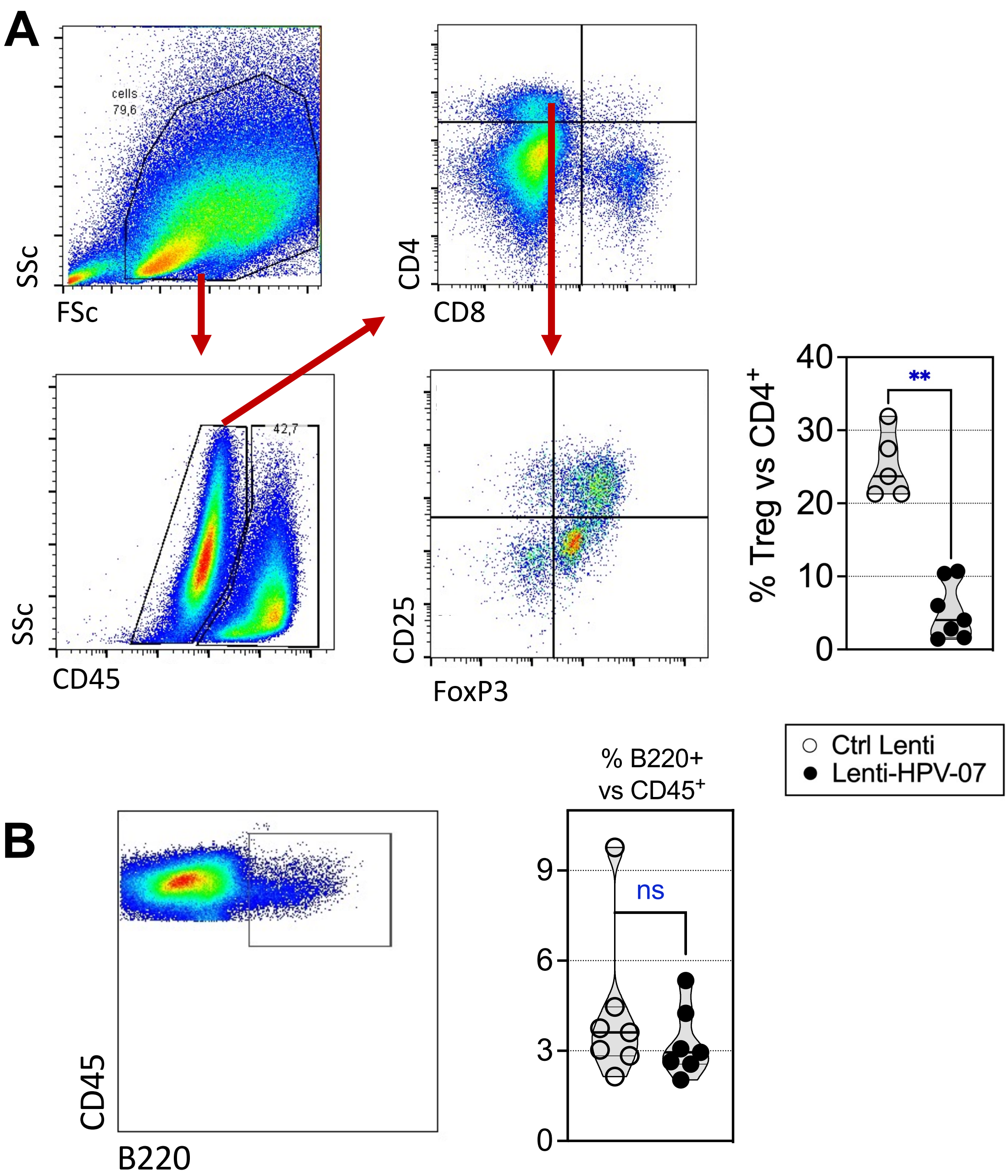

Appendix Figure S2. Decreased proportions of intra-tumoral Treg and unchanged proportions of intra-tumoral B cells in Lenti-HPV-07-vaccinated mice.

(A) Treg cytometric gating strategy and comparative proportions of intra-tumoral Treg populations in Lenti Ctrl- or Lenti-HPV-07-treated C57BL/6 mice studied on day 11 post-vaccination.

(B) Representative dot blot and recapitulative percentages of each subset compared between the two groups. Statistical significance was determined using two-tailed unpaired t tests (ns: not significant, $**p \leq 0.01$).

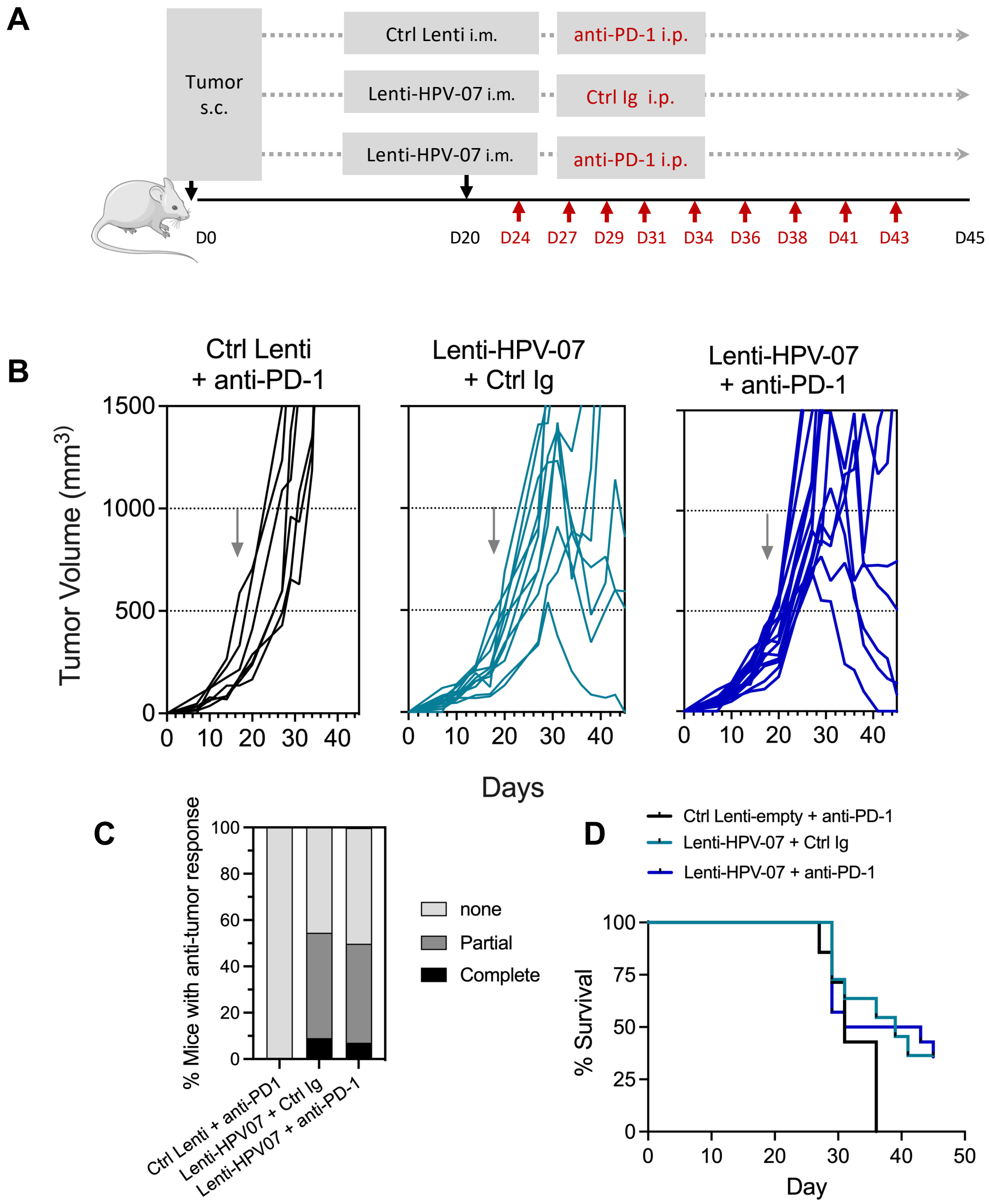

Appendix Figure S3. Anti-PD1 treatment does not synergize with suboptimal dose of Lenti-HPV-07 on very large tumors.

A. Timeline of tumor s.c. engraftment and combinatory Lenti-HPV-07 and anti-PD1 mAb treatment. C57BL/6 mice ($n = 7$ to 14/group) were engrafted on the flank with 1×10^6 tumor cells. On day 20 post-engraftment, when the tumor volume reached an average of 390 mm^3, mice were treated with the suboptimal dose of 1×10^8 TU of Ctrl Lenti or Lenti-HPV-07. Mice were then treated 2 to 3 times a week with anti-PD1 mAb for a total of nine injections from day 24 to 43.

B. Spaghetti plots of tumor growth. The gray arrows indicate the time point at which Ctrl Lenti or Lenti-HPV-07 were injected.

C. Percentages of mice, without, with partial or complete antitumor response.

D. Survival curves of animals, followed for 45 days.

Mice were sacrificed when the size of the tumors reached 1500 mm^3, in accordance with the defined humane endpoints.

A

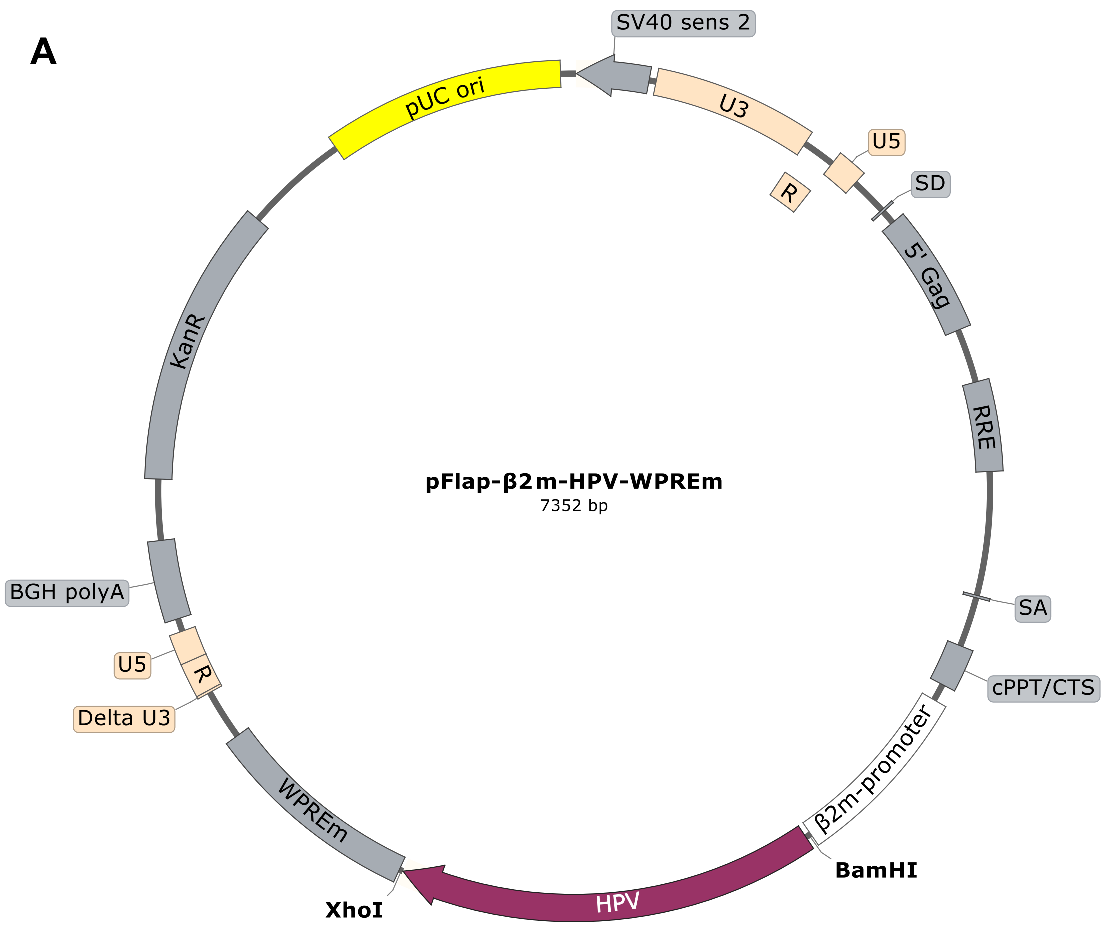

B

$E7_{HPV16}E6_{HPV16}E7_{HPV18}E6_{HPV18}$

PGDTPTLHEYMLDLQPETTDPDRAHYNIVTFCCKCDSTLR**R**CVQSTHV
DIRTLEDLLMGTLGIVCPIasqaFQDPQERPRKLPQLCTELQTTIHDIILE
CVYCKQQLLRREVYDFAFRD**G**CIVYRNPYAVCDKCLKFYSKISEYRHYCY
SLYGTTLEQQYNKPLCDLLIRCINCQKPLRFHNIRGRWTGRCMSCCRsa
gpgpKATLQDIVLHLEPQNEIPVDSEEENDEIDGVNHQHLPARRAEPQ
RHTMLCMCCKCEARIKLVVESSADDLRAFQQLFLNTLSFVCPWvgepg
rtiPYKLPDLCTELNTSLQDIEITCVYCKTVLELTEVFEFAFKD**G**FVVYRDSI
PHAACHKLEKLTNTGLYNLLIRCLRCQKAEKLRHLNEKRRFHNIAG

Appendix Figure S4. Map of the plasmid encoding various HPV-07 poly-antigenic fusion proteins.

(A) The codon-optimized cDNA sequences, encoding various poly-antigens, indicated in Figure 1 were inserted under the β2m promoter in a pFLAP backbone plasmid.

(B) Protein sequence of the HPV-07 antigen, containing 395 aa. The underlined lower-case characters indicate linker sequences between each HPV antigen.

Appendix Table S1. Sequences of primers used in qRT-PCR.

Gene	cDNA accession number	Forward	Reverse	Amplicon (bp)
β2m	NM_009735.3	ATGGGAAGCCGAACATACTG	CAGTCTCAGTGGGGGTGAAT	177
IFNα	NM_010502.2	GGATGTGACCTTCCTCAGACTC	ACCTTCTCCTGCGGGAATCCAA	131
IFNγ	NM_008337.4	TCAAGTGGCATAGATGTGGAAGAA	TGGCTCTGCAGGATTTTCATG	92
TNFα	NM_013693	CATCTTCTCAAAATTCGAGTGACAA	TGGGAGTAGACAAGGTACAACCC	175
TGFβ	NM_011577.2	TGACGTCACTGGAGTTGTACGG	GGTTCATGTCATGGATGGTGC	170
IL-1β	NM_008361.4	TGCCACCTTTTGACAGTGATG	AAGGTCCACGGGAAAGACAC	220
IL-2	NM_008366.3	CCTGAGCAGGATGGAGAATTACA	TCCAGAACATGCCGCAGAG	141
IL-4	NM_021283.2	GTACCAGGAGCCATATCCACG	ACGAGCTCACTCTCTGTGGT	154
IL-5	NM_010558.1	GATGAGGCTTCCTGTCCCTACT	TGACAGGTTTTGGAATAGCATTTCC	130
IL-6	NM_031168.2	CTGCAAGTGCATCATCGTTGTTC	TACCACTTCACAAGTCGGAGGC	116
IL-10	NM_010548.2	CAGTGGAGCAGGTGAAGAGT	GTCCAGCAGACTCAATACACACT	157
IL-12p40	NM_001303244.1	GGAAGCACGGCAGCAGAATA	AACTTGAGGGAGAAGTAGGAATGG	180
IL-18	NM_008360.2	GACAGCCTGTGTTCGAGGATATG	TGTTCTTACAGGAGAGGGTAGAC	159
IL-33	NM_001164724.2	CTACTGCATGAGACTCCGTTCTG	AGAATCCCGTGGATAGGCAGAG	136
CCL2	NM_011333.3	AGGTCCCTGTCATGCTTCTG	TCTGGACCCATTCCTTCTTG	249
CCL3	NM_011337.2	CCTCTGTCAC CTGCTCAACA	GATGAATTGGCGTGGAATCT	163
CCL4	NM_013652.2	TGCAAACCTAACCCCGAGC	TCTGTCTGCCTCTTTTGGTCA	242
CCL5	NM_013653.3	GTGCCCACGTCAAGGAGTAT	GGGAAGCTATACAGGGTCA	185
CCL19	NM_011888.4	CTGCCTCAGATTATCTGCCAT	AGGTAGCGGAAGGCTTTCAC	177
CCL21	NM_002989.4	AAGGCAGTGATGGAGGGG	CGGGGTAAGAACAGGATTG	136
CXCL5	NM_009141.3	GCGTTGTGTTTGCTTAACCG	GAACACTGGCCGTTCTTTCC	242
CXCL9	NM_008599.4	AAAATT TCATCACGCCCTTG	TCTCCAGCTTGGTGAGGTCT	207
CXCL10	NM_021274.2	TGCCGTCATTTTCTGCCTCA	AGGCTCGCAGGGATGATTTC	150
FGF-2	NM_008006.2	AAGCGGCTCTACTGCAAGAA	TGTAACACACTTAGAAGCCAGCA	207
FGF-7	NM_008008.4	CGTGGCAGTTGGAATTGTGG	AGGCAACGAACATTTCCCCT	194
VEGF-C	NM_009506.2	GCTGATGTCTGTCCTGTACCC	AGAAGGTGTTTGTGGCTGCT	239
VCAM-1	NM_011693.3	GACCATGGAGCCTGTCAGTT	CAAACACTTGACCGTGACCG	175
ICAM-1	NM_010493.3	AAGCTGTTTGAGCTGAGCGA	GAGGTCCTTGCCTACTTGCT	165
MCSF	NM_001113529.1	CCTTCTTCGACATGGCTGGG	ATCATCCAGCTGTTCCTGGTC	201
GMCSF	NM_009969.4	GTAGAGGCCATCAAAGAAGCCC	GGTAACTTGTGTTTCACAGTCCG	249
MIF	NM_010798.3	GACTTTTAGCGGCACGAACG	AAGAACAGCGGTGCAGGTAA	238
MMP-2	NM_008610.3	GATAACCTGGATGCCGTCGT	TGGTGTGCAGCGATGAAGAT	188
MMP-9	NM_013599.5	TCTAGGCCCAGAGGTAACCC	TGGAAACTCACACGCCAGAA	240
Col1A1	NM_007742.4	CATGAGCCGAAGCTAACCCC	AGCATACCTCGGGTTTCCAC	209

Amino acid sequences of the detoxified HPV antigens

E6-HPV16

FQDPQERPRKLPQLCTELQTTIHDIILECVYCKQQLLRREVYDFAFRDGCIVYRNPYAVCDKCLKFYSKISEYRHYCYSLYGTTLEQ QYNKPLCDLLIRCINCQKPLRFHNIRGRWTGRCMSCCR

E6-HPV18

PYKLPDLCTELNTSLQDIEITCVYCKTVLELTEVFEFAFKDGFVVYRDSIPHAACHKLEKLTNTGLYNLLIRCLRCQKAEKLRHLNEK RRFHNIAG

E7-HPV16

PGDTPTLHEYMLDLQPETTDPDRAHYNIVTFCCKCDSTLRRCVQSTHVDIRTLEDLLMGTLGIVCPI

E7-HPV18

KATLQDIVLHLEPQNEIPVDSEEENDEIDGVNHQHLPARRAEPQRHTMLCMCCKCEARIKLVVESSADDLRAFQQLFLNTLSFV CPW

Amino acid sequences of HPV poly-antigens

HPV-07

E7-HPV16-E6-HPV16-E7-HPV18-E6-HPV18

MPGDTPTLHEYMLDLQPETTDPDRAHYNIVTFCCKCDSTLRRCVQSTHVDIRTLEDLLMGTLGIVCPIasqaFQDPQERPRKLPQ LCTELQTTIHDIILECVYCKQQLLRREVYDFAFRDGCIVYRNPYAVCDKCLKFYSKISEYRHYCYSLYGTTLEQQYNKPLCDLLIRCIN CQKPLRFHNIRGRWTGRCMSCCRsagpgpKATLQDIVLHLEPQNEIPVDSEEENDEIDGVNHQHLPARRAEPQRHTMLCMCC KCEARIKLVVESSADDLRAFQQLFLNTLSFVCPWvgepgrtiPYKLPDLCTELNTSLQDIEITCVYCKTVLELTEVFEFAFKDGFVVYR DSIPHAACHKLEKLTNTGLYNLLIRCLRCQKAEKLRHLNEKRRFHNIAGh

HPV-08

E6-HPV16-E6-HPV18-E7-HPV16-E7-HPV18

MFQDPQERPRKLPQLCTELQTTIHDIILECVYCKQQLLRREVYDFAFRDGCIVYRNPYAVCDKCLKFYSKISEYRHYCYSLYGTTLE QQYNKPLCDLLIRCINCQKPLRFHNIRGRWTGRCMSCCRgpddPYKLPDLCTELNTSLQDIEITCVYCKTVLELTEVFEFAFKDGF VVYRDSIPHAACHKLEKLTNTGLYNLLIRCLRCQKAEKLRHLNEKRRFHNIAGPGDTPTLHEYMLDLQPETTDPDRAHYNIVTFC CKCDSTLRRCVQSTHVDIRTLEDLLMGTLGIVCPIgpddKATLQDIVLHLEPQNEIPVDSEEENDEIDGVNHQHLPARRAEPQRH TMLCMCCKCEARIKLVVESSADDLRAFQQLFLNTLSFVCPWa

HPV-09

E6-HPV18-E6-HPV16-E7-HPV18-E7-HPV16

MrrPYKLPDLCTELNTSLQDIEITCVYCKTVLELTEVFEFAFKDGFVVYRDSIPHAACHKLEKLTNTGLYNLLIRCLRCQKAEKLRHL NEKRRFHNIAGhFQDPQERPRKLPQLCTELQTTIHDIILECVYCKQQLLRREVYDFAFRDGCIVYRNPYAVCDKCLKFYSKISEYRH YCYSLYGTTLEQQYNKPLCDLLIRCINCQKPLRFHNIRGRWTGRCMSCCRsagpgpKATLQDIVLHLEPQNEIPVDSEEENDEIDG VNHQHLPARRAEPQRHTMLCMCCKCEARIKLVVESSADDLRAFQQLFLNTLSFVCPWagPGDTPTLHEYMLDLQPETTDPDR AHYNIVTFCCKCDSTLRRCVQSTHVDIRTLEDLLMGTLGIVCPIa

HPV-10

E7-HPV18-E6-HPV18-E7-HPV16-E6-HPV16

MgpKATLQDIVLHLEPQNEIPVDSEEENDEIDGVNHQHLPARRAEPQRHTMLCMCCKCEARIKLVVESSADDLRAFQQLFLNT LSFVCPgepgrtiPYKLPDLCTELNTSLQDIEITCVYCKTVLELTEVFEFAFKDGFVVYRDSIPHAACHKLEKLTNTGLYNLLIRCLRCQ KAEKLRHLNEKRRFHNIAGPGDTPTLHEYMLDLQPETTDPDRAHYNIVTFCCKCDSTLRRCVQSTHVDIRTLEDLLMGTLGIVCP IasqaFQDPQERPRKLPQLCTELQTTIHDIILECVYCKQQLLRREVYDFAFRDGCIVYRNPYAVCDKCLKFYSKISEYRHYCYSLYGT TLEQQYNKPLCDLLIRCINCQKPLRFHNIRGRWTGRCMSCCRs

Nucleic sequences of HPV poly-antigens

ggatcc : restriction enzyme BamHI
GCCACC : Kozak *sequence*
ATG : initiation codon
TGATAA : Double STOP codon
ctcgag : restriction enzyme XhoI

Codon optimized **HPV-07** (E7-HPV16-E6-HPV16-E7-HPV18-E6-HPV18) sequence :

ggatccGCCACCATGCCCGGAGACACCCCCACCCTGCACGAATACATGCTGGACCTGCAGCCCGAAACCACCGACCCCGAC
CGCGCTCACTACAACATCGTTACATTCTGTTGTAAATGCGACTCCACCCTGAGAAGATGCGTGCAGTCCACCCACGTGGAC
ATCAGGACCCTGGAGGACCTCCTCATGGGAACCCTGGGTATCGTCTGCCCCATCgcctcccaggctTTTCAGGACCCCCAGGA
AAGGCCCAGGAAGTTGCCCCAGCTCTGCACCGAACTGCAGACCACCATTCATGACATCATCCTCGAATGCGTGTACTGCA
AGCAGCAGCTCCTGAGGAGGGAGGTGTACGATTTCGCCTTCAGAGACGGCTGTATCGTCTACAGGAACCCCTATGCCGTC
TGCGACAAATGCCTGAAGTTTTATTCCAAGATCTCCGAGTACAGGCACTATTGCTACAGCCTGTATGGGACCACCCTGGAG
CAGCAGTACAACAAGCCCCTGTGCGACCTCCTGATCAGGTGCATCAACTGCCAGAAGCCCCTGAGGTTCCACAACATCCG
CGGCAGGTGGACCGGAAGGTGCATGTCCTGCTGCAGGtccgccggccccggacctAAAGCCACCCTCCAGGACATCGTTCTCCA
CCTGGAGCCCCAGAACGAGATCCCCGTGGACTCAGAAGAGGAGAACGACGAGATCGACGGCGTCAACCACCAGCACCTG
CCCGCTCGCAGAGCCGAACCCCAGAGACACACCATGCTCTGCATGTGCTGCAAATGCGAAGCCCGGATTAAGTTGGTGGT
GGAAAGCAGCGCCGACGATCTGAGGGCCTTCCAGCAGCTCTTCCTCAACACCCTGTCCTTCGTGTGCCCCTGGgtgggcgag
cccggtagaaccatcCCCTACAAGCTGCCCGATCTGTGCACAGAGCTGAACACCTCCCTGCAGGACATCGAGATCACCTGCGTC
TACTGCAAGACCGTGCTGGAACTGACCGAGGTGTTCGAATTCGCCTTCAAGGACGGCTTCGTGGTGTACAGGGACAGCAT
TCCCCACGCCGCCTGCCATAAGCTGGAGAAACTGACCAACACCGGACTGTATAACCTGCTGATCAGGTGTCTGAGGTGCC
AGAAGGCAGAGAAACTGAGACATCTGAACGAGAAAAGGAGGTTCCACAATATTGCCGGGcacTGATAActcgag

Codon optimized **HPV-08** (E6-HPV16-E6-HPV18-E7-HPV16-E7-HPV18) sequence :

ggatccGCCACCATGTTCCAGGACCCCCAGGAGAGGCCCCGGAAGTTGCCCCAGCTGTGCACCGAGCTGCAGACCACCATC
CACGACATCATCCTCGAATGCGTGTACTGCAAGCAGCAGCTGCTGAGGAGGGAGGTGTATGACTTTGCCTTCAGAGACG
GATGCATTGTCTACAGGAACCCCTACGCCGTGTGCGACAAATGCCTGAAGTTCTACTCCAAGATCAGCGAGTACAGGCAC
TACTGCTACTCCCTGTACGGCACCACCCTCGAACAGCAGTACAACAAACCCCTGTGCGACCTCCTGATTAGGTGCATCAAC
TGCCAGAAGCCCCTCAGGTTCCACAACATCCGCGGCCGCTGGACCGGCCGATGCATGTCTTGCTGCAGGggccccgacgacC
CCTACAAGCTCCCCGACCTGTGCACCGAACTCAACACCTCCCTGCAGGACATCGAGATCACCTGCGTGTATTGCAAGACCG
TGCTGGAGCTGACCGAGGTTTTCGAATTTGCCTTTAAGGACGGCTTCGTCGTGTATAGGGACTCCATCCCCCACGCCGCCT
GCCATAAGCTGGAGAAGCTCACCAACACCGGACTGTATAATCTGCTGATCAGGTGCCTCAGGTGCCAGAAGGCAGAAAA
GCTGAGGCATCTCAACGAGAAGCGCCGGTTCCACAATATTGCCGGCCCCGGAGACACCCCCACACTCCATGAGTACATGC
TCGACCTGCAGCCCGAAACCACCGACCCCGACAGAGCCCACTACAACATCGTGACCTTCTGCTGCAAGTGCGACTCCACCC
TGAGAAGATGCGTGCAGTCCACCCACGTGGACATCCGCACACTCGAAGACCTGCTGATGGGAACCCTGGGCATCGTGTG
CCCCATCggccccgatgacAAGGCCACCTTGCAGGACATCGTGCTGCACCTGGAACCACAGAACGAGATCCCCGTCGACTCCG
AAGAAGAAAACGACGAAATCGACGGAGTGAATCACCAGCACCTGCCCGCCAGAAGGGCCGAGCCTCAGAGACACACCA
TGCTCTGCATGTGCTGCAAATGCGAAGCCAGGATTAAGCTGGTGGTGGAGAGCAGCGCCGACGACCTGAGGGCCTTCCA
GCAGCTCTTCCTGAACACACTGTCCTTCGTGTGCCCCTGGgccTGATAActcgag

Codon optimized **HPV-09** (E6-HPV18-E6-HPV16-E7-HPV18-E7-HPV16) sequence :

ggatccGCCACCATGaggcggCCCTACAAGCTGCCCGACCTGTGCACCGAGCTGAACACCTCCCTGCAGGACATCGAGATCAC
CTGCGTGTACTGCAAGACCGTGCTGGAGCTGACCGAGGTGTTCGAATTCGCATTCAAGGACGGATTCGTCGTGTATAGG
GACAGCATTCCACACGCCGCCTGCCACAAGCTGGAGAAATTGACTAACACCGGACTGTATAATCTGCTGATCCGGTGCCT
GAGGTGTCAGAAGGCCGAGAAGCTGAGGCATCTGAACGAGAAAAGGAGATTCCACAATATCGCCGGAcacTTCCAGGAC
CCCCAGGAGAGGCCCAGGAAACTGCCCCAGTTGTGCACCGAGCTCCAGACAACCATCCACGACATCATCCTGGAGTGCGT
GTACTGTAAGCAGCAGTTGCTGAGGAGAGAGGTGTATGACTTCGCCTTCAGAGACGGATGCATTGTCTATAGGAACCCCT
ACGCCGTGTGCGACAAGTGCCTGAAGTTCTACTCCAAGATCAGTGAGTACAGGCATTACTGCTACAGCCTGTATGGAACC

ACACTGGAACAGCAGTACAACAAGCCCCTGTGCGACCTCCTGATTAGGTGCATCAACTGCCAGAAGCCCCTCAGGTTCCA
CAACATCCGGGGCAGGTGGACCGGAAGGTGCATGTCCTGCTGCAGGtccgccggccccggacctAAAGCCACCCTCCAGGACA
TCGTGCTGCACCTGGAGCCCCAGAACGAGATCCCCGTCGACTCAGAGGAGGAGAACGACGAAATTGACGGCGTCAACCA
CCAGCACCTGCCCGCTCGCAGAGCCGAACCCCAGAGACACACCATGCTCTGCATGTGCTGCAAATGCGAGGCCCGGATTA
AGCTGGTGGTGGAGAGCTCCGCCGACGATCTGAGAGCCTTCCAGCAGCTCTTCCTGAACACCCTGTCCTTCGTGTGCCCCT
GGgccggtCCCGGTGACACACCTACCCTGCACGAGTACATGCTCGATCTGCAGCCCGAGACCACCGACCCCGATCGCGCAC
ACTACAACATCGTGACCTTCTGCTGCAAATGTGACAGCACCCTGAGACGGTGCGTCCAGTCCACCCACGTTGACATCCGCA
CCCTCGAAGACCTGCTCATGGGAACCCTGGGCATCGTGTGCCCCATCgccTGATAActcgag

Codon optimized **HPV-10** (E7-HPV18-E6-HPV18-E7-HPV16-E6-HPV16) sequence :

ggatccGCCACCATGggccctAAGGCCACCCTGCAGGACATCGTGCTGCACTTGGAGCCCCAGAACGAGATCCCCGTGGACA
GCGAGGAGGAGAACGACGAAATCGACGGCGTGAACCACCAGCACCTGCCCGCAAGAAGGGCCGAACCCCAGAGGCACA
CCATGCTCTGCATGTGCTGCAAATGCGAGGCCAGGATCAAGCTGGTGGTGGAAAGCAGCGCCGACGATCTGAGGGCATT
CCAGCAGCTGTTCCTGAACACCCTCTCCTTCGTGTGCCCTggggaacccggcaggaccatcCCCTATAAACTGCCCGACCTCTGCA
CCGAGCTGAACACCTCCCTGCAGGACATTGAGATCACCTGCGTCTACTGCAAAACCGTCCTGGAACTGACCGAGGTGTTC
GAGTTCGCCTTCAAAGACGGCTTCGTCGTGTACAGGGACAGCATCCCCCACGCCGCCTGCCATAAGCTGGAGAAACTGAC
CAACACCGGCCTGTACAACCTGCTGATCCGGTGCCTGAGATGTCAGAAGGCCGAGAAACTGAGGCACCTCAACGAGAAA
AGGAGATTCCACAATATTGCCGGGCCCGGCGACACCCCAACCCTGCACGAATACATGCTCGACCTGCAGCCCGAAACCAC
CGACCCCGACAGAGCCCACTACAACATCGTGACCTTCTGCTGCAAGTGCGACTCCACCCTGAGAAGATGCGTGCAGTCCA
CCCACGTGGACATCCGCACACTCGAAGACCTGCTGATGGGAACCCTGGGCATCGTGTGCCCCATCgcttcccaggccTTTCAG
GACCCCCAGGAACGGCCAAGAAAGCTGCCCCAGCTCTGCACCGAACTGCAGACCACCATCCACGACATCATCCTGGAATG
CGTCTACTGTAAGCAGCAGTTGCTGAGGAGGGAGGTGTATGATTTCGCCTTCAGAGACGGCTGCATCGTCTACAGGAACC
CCTACGCCGTGTGCGACAAATGCCTGAAGTTCTACTCCAAGATCTCCGAATACAGACACTATTGCTACAGCCTGTACGGCA
CCACCCTCGAACAGCAGTACAACAAACCCCTGTGCGACCTCCTGATCAGGTGCATCAACTGCCAGAAGCCCCTCCGGTTCC
ACAACATCCGAGGAAGATGGACCGGCCGGTGCATGTCCTGCTGCAGGtccTGATAActcgag
