# Supplementary material for: Full eradication of pre‐clinical human papilloma virus‐induced tumors by a lentiviral vaccine
Source: EMBO Mol Med. 2023 Sep 7;15(10):e17723. doi: 10.15252/emmm.202317723 (PMC10565635; doi:10.15252/emmm.202317723)
Supplement: Supplementary file 2 — Expanded View Figures PDF [file EMMM-15-e17723-s004.pdf]

Expanded View Figures

$E6_{HPV\ 16}$ Native

~~MHQKRTAM~~FQDPQERPRKLPQLCTELQTTIHDIILECVYCKQQLLRREVYDFAFRD**L**CIVYR~~DG~~NPYAVCDKCLKFYSKI
SEYRHYCYSLYGTTLEQQYNKPLCDLLIRCINCQKPL~~CPEEKQRHLDKKQ~~RFHNIRGRWTGRCMSCCR~~SSRTRRETQL~~

$E6_{HPV\ 16}$ in Lenti-HPV

FQDPQERPRKLPQLCTELQTTIHDIILECVYCKQQLLRREVYDFAFRD**G**CIVYRNPYAVCDKCLKFYSKISEYRHYCYSLYG
TTLEQQYNKPLCDLLIRCINCQKPLRFHNIRGRWTGRCMSCCR

$E7_{HPV\ 16}$ Native

~~M~~**H**GDTPTLHEYMLDLQPETTD~~LYCYEQLNDSSEEEDEIDGPAGQAE~~PDRAHYNIVTFCCKCDSTLR**L**CVQSTHVDIRT
LEDLLMGTLGIVCPI~~CSQKP~~

$E7_{HPV\ 16}$ in Lenti-HPV

PGDTPTLHEYMLDLQPETTDPDRAHYNIVTFCCKCDSTLR**R**CVQSTHVDIRTLEDLLMGTLGIVCPI

$E6_{HPV\ 18}$ Native

~~MARFEDPTRR~~PYKLPDLCTELNTSLQDIEITCVYCKTVLELTEVFEFAFKD**L**FVVYRDSIPHAACHK~~CIDFYSRIRELRHYS~~
~~DSVYGDT~~LEKLTNTGLYNLLIRCLRCQK~~PLNP~~AEKLRHLNEKRRFHNIAG~~HYRGQCHSCCNRARQERLQRRRETQV~~

$E6_{HPV\ 18}$ in Lenti-HPV

PYKLPDLCTELNTSLQDIEITCVYCKTVLELTEVFEFAFKD**G**FVVYRDSIPHAACHKLEKLTNTGLYNLLIRCLRCQKAEKLR
HLNEKRRFHNIAG

$E7_{HPV\ 18}$ Native

~~MHGP~~KATLQDIVLHLEPQNEIPVD~~LLCHEQLSD~~SEEENDEIDGVNHQHLPARRAEPQRHTMLCMCCKCEARIKLVVES
SADDLRAFQQLFLNTLSFVCPW~~CASQQ~~

$E7_{HPV\ 18}$ in Lenti-HPV

KATLQDIVLHLEPQNEIPVDSEEENDEIDGVNHQHLPARRAEPQRHTMLCMCCKCEARIKLVVESSADDLRAFQQLFL
NTLSFVCPW

Figure EV1. Protein sequence of each HPV antigen encoded by Lenti-HPV vectors.

Protein sequence of each HPV antigen, the native sequence and that included in the Lenti-HPV vectors. The segments underlined in the native sequences are those deleted in the Lenti-HPV antigen design. The a.a. substitutions are indicated in bold characters on the sequences included in Lenti-HPV. The native $E6_{HPV\ 16}$ sequence has been deleted for the segments 1–8, 63–64, 118–130, 149–158 and substituted at the position 57 of the native sequence (L57G). L57 is part of the binding site to the p53 tumor suppressor protein and activation of telomerase. Note that the deleted 149–158 segment encompasses the PDZ-interacting domain (156–158). The native $E7_{HPV\ 16}$ sequence has been deleted for the a.a. at the position 1 and a.a. segments 22–46, 94–98 and substituted at the positions 2 (H2P), that is, at the binding site to the Rb tumor suppressor protein, and 67 (L67R), that is, at the binding site to CHD4, of the native sequence. The native $E6_{HPV\ 18}$ sequence has been deleted for the a.a. segments 1–10, 68–89, 111–114, 133–158 and substituted at the position 52 of the native sequence (L52R), part of the binding site to the p53 tumor suppressor protein. Note that the deleted 133–158 segment encompasses the PDZ-interacting domain (156–158). The native $E7_{HPV\ 18}$ sequence has been deleted for the a.a. segments 1–4, 25–33, and 101–105.

© 2023 The Authors

Figure EV2. T-cell immunogenicity of various Lenti-HPV against each selected HPV antigen and efficacy of mAb-mediated T subset depletion for functional analysis.

A Comparable immunogenicity of various Lenti-HPV. C57BL/6 mice (n = 5/group) were immunized i.m. at day 0 with Ctrl Lenti or each of the Lenti-HPV. On day 14, T splenocyte responses were assessed by IFN-γ ELISPOT after *in vitro* stimulation with a pool of 15-mer peptides spanning the sequence of $E6_{HPV16}$, $E6_{HPV18}$, $E7_{HPV16}$ or $E7_{HPV18}$, as detailed in Fig 2A. Comparable T-cell immunogenicity of the four Lenti-HPVs showed that the position of the antigen in the four poly-antigens encoded by these vectors has no effect on their immunogenicity.

B Efficacy of anti-CD4 or anti-CD8 depletion *in vivo*. Tumor-free C57BL/6 mice (n = 2/group) received two i.p. injections of 250 μg anti-CD4 (clone GK1.5), anti-CD8 (clone H35.17.2) or a control Ig isotype 3 days apart. On day 4 after the second injection, the spleens from individual mice were assessed by anti-CD3, anti-CD4, and anti-CD8 mAb staining and cytometry analysis to evaluate the efficacy of T-subset depletion. The results from two individual mice are shown. Note that the mAbs used for depletion and cytometric studies were different to allow the distinction between epitope masking and genuine T-subset depletion.

Source data are available online for this figure.

 © 2023 The Authors

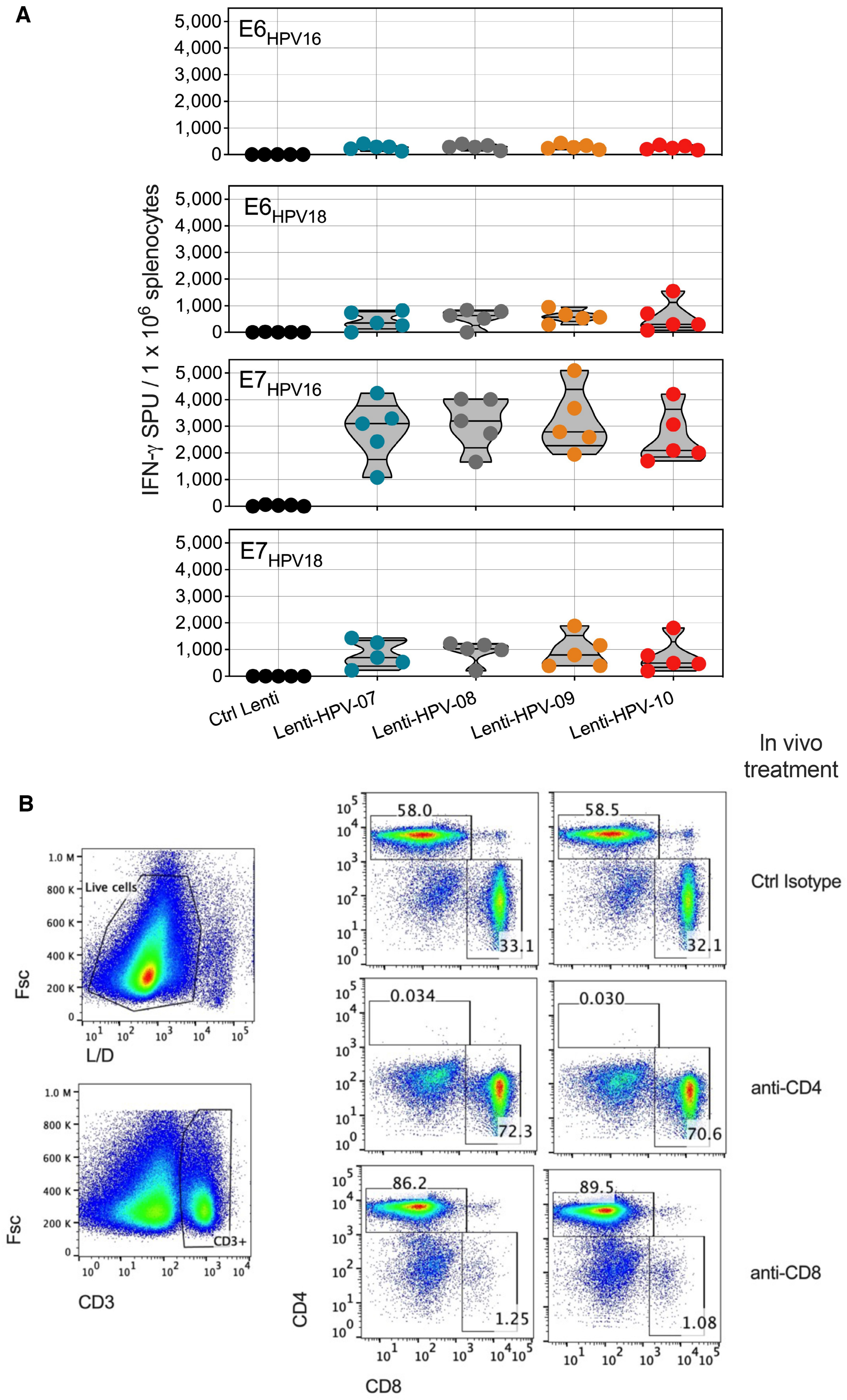
A
E6HPV16
E6HPV18
E7HPV16
E7HPV18
IFN-γ SPU / 1 x 10^6 splenocytes
5,000
4,000
3,000
2,000
1,000
0
Ctrl Lenti
Lenti-HPV-07
Lenti-HPV-08
Lenti-HPV-09
Lenti-HPV-10
B
Live cells
Fsc
L/D
CD3+
CD3
In vivo treatment
58.0
33.1
58.5
32.1
Ctrl Isotype
0.034
72.3
0.030
70.6
anti-CD4
86.2
1.25
89.5
1.08
anti-CD8
CD4
CD8

Figure EV2.

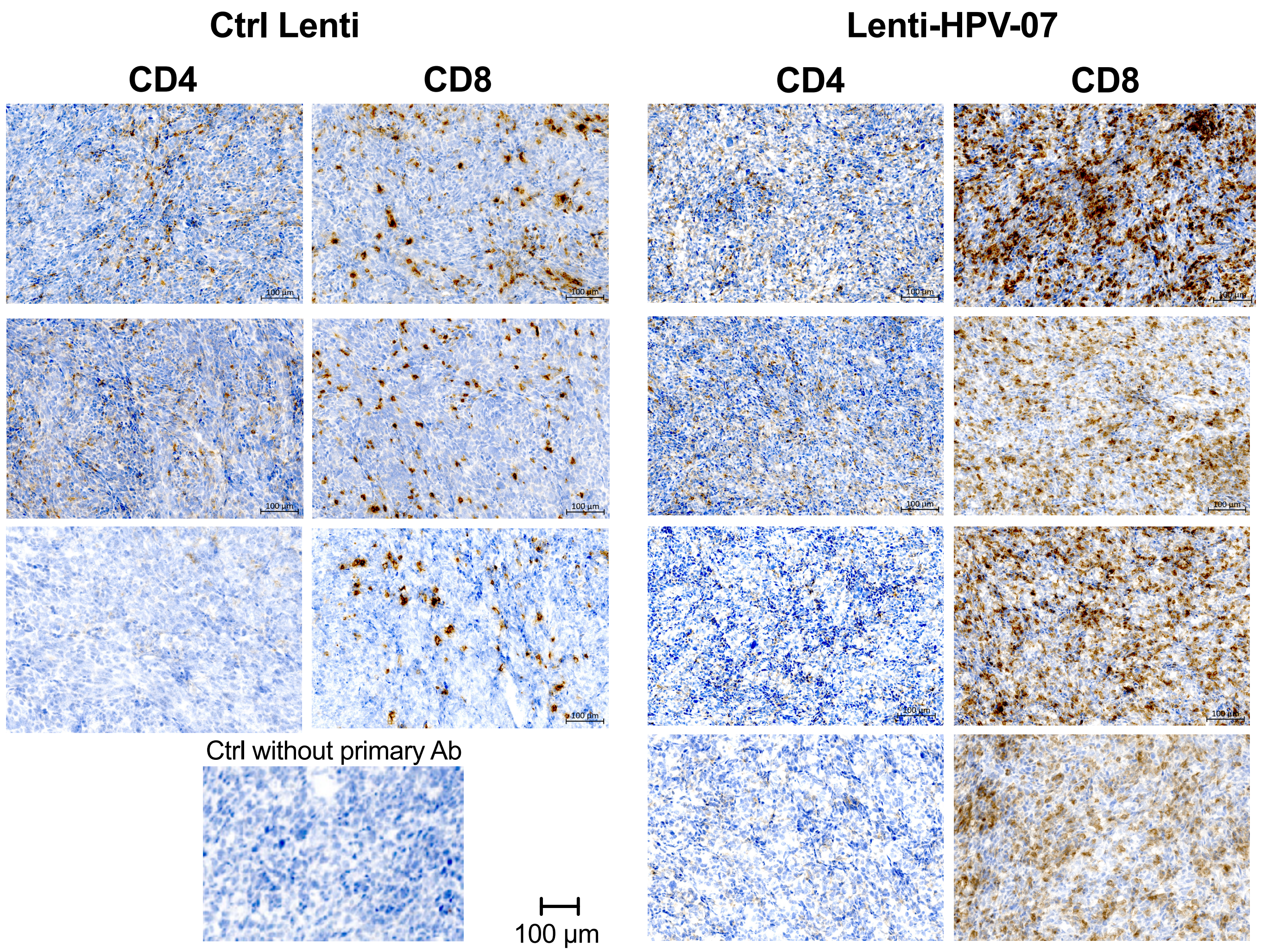

Figure EV3. Comparative immunohistochemistry analysis of tumor infiltrating T cells in Ctrl Lenti- or Lenti-HPV-07-treated, tumor-bearing mice.

C57BL/6 mice were engrafted s.c. with 1×10^6 tumor cells and injected with 1×10^9 TU of Lenti Ctrl or Lenti-HPV-07 ($n = 4$–5). Tumor-infiltrating T cells were studied by CD4 or CD8 immunohistochemistry on day 11 post-vaccination. Tumors from individual mice are shown.

Source data are available online for this figure.

Figure EV4. No role for NK cells in the tumor regression of TC-1 cells induced by Lenti-HPV-07 therapy.

A Timeline of the tumor engraftment, Lenti-HPV-07 treatment and anti-NK1.1 mAb injection. C57BL/6 mice ($n = 7$/group) were engrafted s.c. on the flank with 1×10^6 tumor cells. On day 14 post-engraftment, mice were injected with 1×10^9 TU of Ctrl Lenti or Lenti-HPV-07. From day 13 to 30 mice were treated with a control Ig or anti-NK1.1 mAb by 9 successive i.p. injection of 300 µg/mouse/injection.

B Efficacy of anti-NK1.1 depletion *in vivo* was verified on the splenoctyes of one mouse per group assessed by anti-CD11b and anti-NKp46 mAb staining and cytometry analysis. Note that the mAbs used for NK cell depletion and cytometric studies target distinct markers to ensure genuine NK cell depletion and not epitope masking.

C Spaghettis plots of tumor growth in the mice.

Source data are available online for this figure.

 © 2023 The Authors

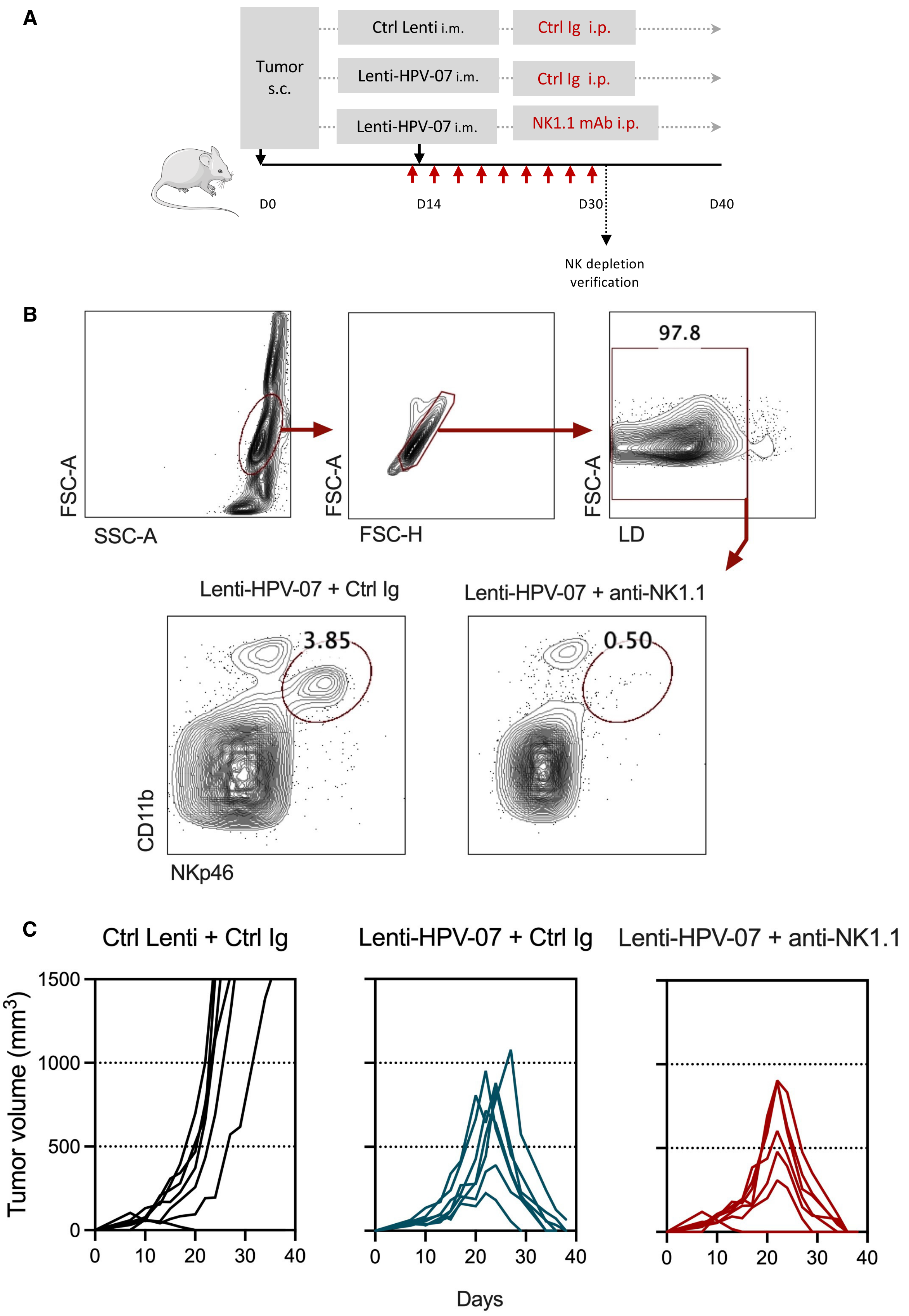
A
Tumor s.c.
Ctrl Lenti i.m.
Ctrl Ig i.p.
Lenti-HPV-07 i.m.
Ctrl Ig i.p.
Lenti-HPV-07 i.m.
NK1.1 mAb i.p.
D0
D14
D30
D40
NK depletion verification
B
FSC-A
SSC-A
FSC-A
FSC-H
97.8
FSC-A
LD
Lenti-HPV-07 + Ctrl Ig
3.85
Lenti-HPV-07 + anti-NK1.1
0.50
CD11b
NKp46
C
Ctrl Lenti + Ctrl Ig
Lenti-HPV-07 + Ctrl Ig
Lenti-HPV-07 + anti-NK1.1
Tumor volume (mm3)
1500
1000
500
0
0 10 20 30 40
Days

Figure EV4.

Figure EV5. Failure of combinatory anti-PD1 treatment and suboptimal vaccination with Lenti-HPV-07 when the two treatments are given simultaneously.

A Timeline of the tumor engraftment and combinatory treatment with Lenti-HPV-07 vaccination and anti-PD1 injections. C57BL/6 mice (n = 10/group) were engrafted s.c. on the flank with 1×10^6 tumor cells. On day 10 post-engraftment, when the tumor volume reached an average of 100–120 mm^3, mice were injected with a suboptimal dose of 1×10^8 TU Ctrl Lenti or Lenti-HPV-07. Mice were treated with anti-PD1 mAb on the same day and on day 13.

B Spaghettis plots of tumor growth in the mice. The gray arrows indicate the time point at which the vaccine was injected.

C Survival curves of the animals (n = 10/group) shown in (B) followed for 130 days. Statistical significance was determined by Log-rank Mantel-Cox tests (ns, not significant, $^{**}P \leq 0.01$, $^{***}P \leq 0.001$). Note that there is a net tendency to decline in the survival of mice treated with Lenti-HPV-07 + anti-PD1 compared to the mice treated with Lenti-HPV-07 alone, even though this difference did not reach statistical significance. Mice were sacrificed when the size of the tumors reached 1,500 mm^3, in accordance with the defined humane endpoints.

Source data are available online for this figure.

 © 2023 The Authors

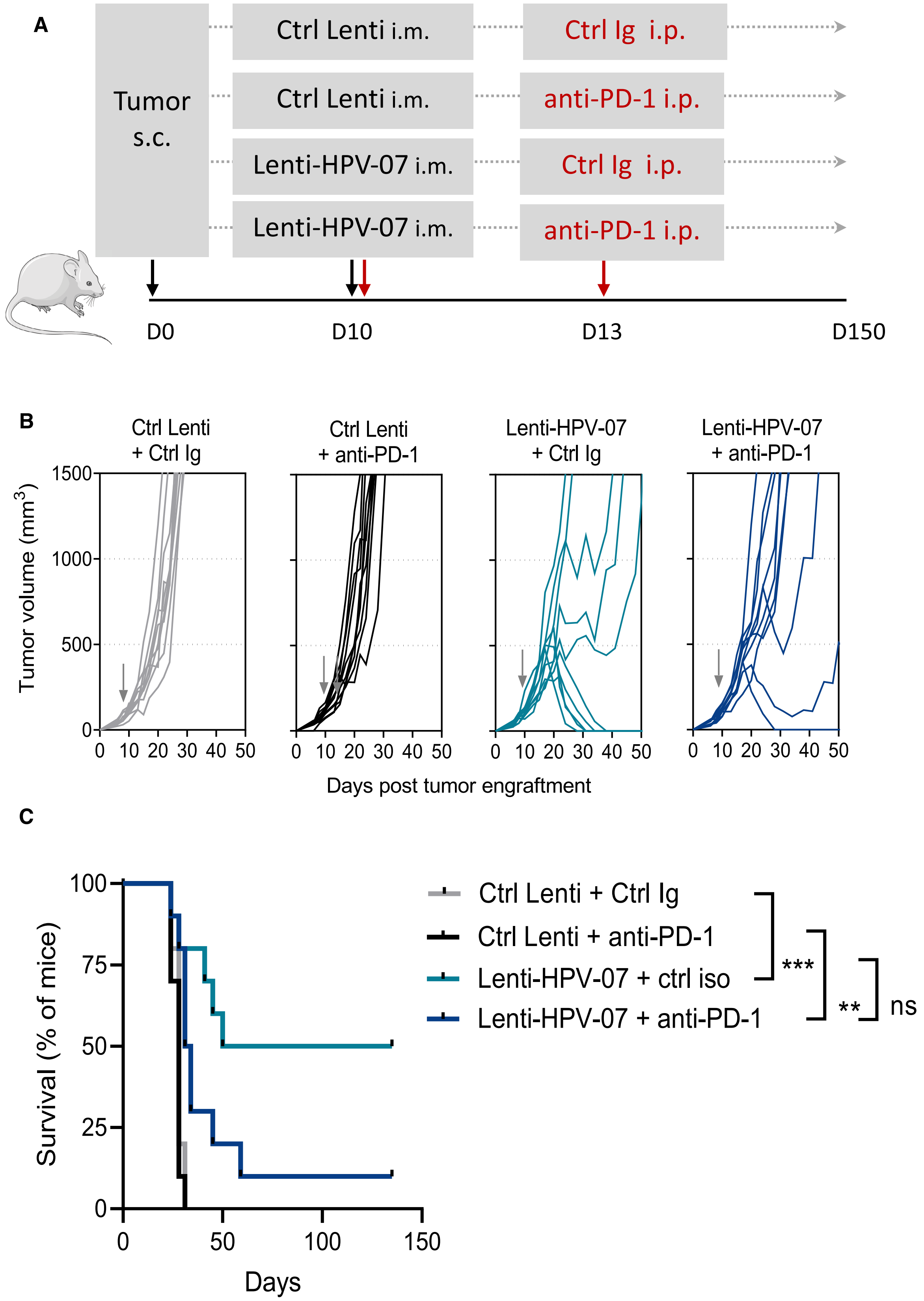
A
Tumor s.c.
Ctrl Lenti i.m.
Ctrl Ig i.p.
Ctrl Lenti i.m.
anti-PD-1 i.p.
Lenti-HPV-07 i.m.
Ctrl Ig i.p.
Lenti-HPV-07 i.m.
anti-PD-1 i.p.
D0
D10
D13
D150
B
Ctrl Lenti + Ctrl Ig
Ctrl Lenti + anti-PD-1
Lenti-HPV-07 + Ctrl Ig
Lenti-HPV-07 + anti-PD-1
Tumor volume (mm3)
Days post tumor engraftment
C
Survival (% of mice)
Days
Ctrl Lenti + Ctrl Ig
Ctrl Lenti + anti-PD-1
Lenti-HPV-07 + ctrl iso
Lenti-HPV-07 + anti-PD-1
***
**
ns

Figure EV5.
