# Supplementary material for: Full eradication of pre‐clinical human papilloma virus‐induced tumors by a lentiviral vaccine
Source: EMBO Mol Med. 2023 Sep 7;15(10):e17723. doi: 10.15252/emmm.202317723 (PMC10565635; doi:10.15252/emmm.202317723)
Supplement: Supplementary file 3 — PDF+ [file EMMM-15-e17723-s008.pdf]

SOURCE DATA TRANSPARENT PROCESS OPEN ACCESS

EMBO Molecular Medicine

Article

Full eradication of pre-clinical human papilloma virus-induced tumors by a lentiviral vaccine

Laëtitia Douguet[1,†], Ingrid Fert[1,†], Jodie Lopez[1,†], Benjamin Vesin[1,†], Fabien Le Chevalier[1], Fanny Moncoq[1], Pierre Authié[1], Trang-My Nguyen[1], Amandine Noirat[1], Fabien Névo[1], Catherine Blanc[1], Maryline Bourgine[1], David Hardy[2], François Anna[1], Laleh Majlessi[1,*,‡] & Pierre Charneau[1,‡]

Abstract

Human papillomavirus (HPV) infections are the cause of all cervical and numerous oropharyngeal and anogenital cancers. The currently available HPV vaccines, which induce neutralizing antibodies, have no therapeutic effect on established tumors. Here, we developed an immuno-oncotherapy against HPV-induced tumors based on a non-integrative lentiviral vector encoding detoxified forms of the Early E6 and E7 oncoproteins of HPV16 and 18 genotypes, namely, "Lenti-HPV-07". A single intramuscular injection of Lenti-HPV-07 into mice bearing established HPV-induced tumors resulted in complete tumor eradication in 100% of the animals and was also effective against lung metastases. This effect correlated with CD8^{+} T-cell induction and profound remodeling of the tumor microenvironment. In the intra-tumoral infiltrates of vaccinated mice, the presence of large amounts of activated effector, resident memory, and transcription factor T cell factor-1 (TCF-1)$^{+}$ "stem-like" CD8^{+} T cells was associated with full tumor eradication. The Lenti-HPV-07-induced immunity was long-lasting and prevented tumor growth after a late re-challenge, mimicking tumor relapse. Lenti-HPV-07 therapy synergizes with an anti-checkpoint inhibitory treatment and therefore shows promise as an immuno-oncotherapy against established HPV-mediated malignancies.

Keywords early E6-E7 oncoproteins; immuno-oncotherapy; intra-tumoral immune cells; lentiviral vector; tumor microenvironment
Subject Categories Cancer; Immunology; Microbiology, Virology & Host Pathogen Interaction
DOI 10.15252/emmm.202317723 | Received 20 March 2023 | Revised 22 August 2023 | Accepted 23 August 2023 | Published online 7 September 2023
EMBO Mol Med (2023) 15: e17723

Introduction

Persistent human papillomavirus (HPV) infections are responsible for all cervical and numerous oropharyngeal and anogenital cancers (de Sanjose *et al*, 2018). At least 14 of 100 related HPV types thus far described can cause malignant transformation. The two most abundant types, HPV16 followed by HPV18, are the most high-risk types and are responsible for 71% of cervical cancers (Zottnick *et al*, 2020). At least 3% of oral, 12% of pharyngeal, and 30–60% of oropharyngeal cancers are caused by HPV (Kobayashi *et al*, 2018). The early (E) E6 and E7 HPV oncoproteins initiate and maintain the malignant phenotype of infected host cells (zur Hausen, 2002). While E6 induces inactivation of p53 or its proteasomal degradation, E7 inactivates the retinoblastoma protein. Together, these events lead to persistent viral DNA synthesis, abnormal host cell division, and tumor development (Doorbar *et al*, 2012). All the tumor cells from HPV-associated cancers express E6 and E7, which are thus major viral antigens to target in antitumor therapeutic vaccination approaches (Steinbach & Riemer, 2018; Lechner *et al*, 2022).

The currently available prophylactic HPV vaccines, that is, Cervarix, Gardasil and Gardasil 9, bivalent, quadrivalent, and nonavalent vaccines, respectively, are based on the main L1 protein of the HPV capsid, which forms immunogenic virus-like particles (VLPs), adjuvanted in alum and/or monophosphoryl lipid A (Rosalik *et al*, 2021; Hampson, 2022). These vaccines mainly induce HPV-neutralizing antibodies which prevent viral infection but have no therapeutic effect on already infected or malignant cells. Therefore, when administered after the onset of sexual life, these vaccines provide no significant protection, except in individuals not yet infected with HPV (Rosalik *et al*, 2021; Hampson, 2022). The efficacy of future therapeutic vaccines will rely on their ability to elicit HPV-specific T-cell responses that are able to target oncoprotein-

1 Virology Department, Pasteur-TheraVectys Joint Lab, Institut Pasteur, Université de Paris, Paris, France
2 Histopathology Platform, Institut Pasteur, Université de Paris, Paris, France
*Corresponding author. Tel: +33 1 40 61 32 68; E-mail: laleh.majlessi@pasteur.fr
†These authors contributed equally to this work as first authors
‡These authors contributed equally to this work as senior authors

© 2023 The Authors. Published under the terms of the CC BY 4.0 license

expressing dysplastic precancerous and malignant epithelial cells to confer a long-lasting memory response. Several therapeutic vaccines, based on adjuvanted peptides or proteins or viral or bacterial vectors expressing E6/E7 oncoproteins have been investigated, mostly as treatments for cervical cancer. However, no notable long-term antitumor immunity or improved clinical outcome has been recorded (Lechner *et al*, 2022), highlighting the need to progress toward more effective curative vaccines.

Lentiviral vectors (LVs) are promising vaccination vectors for immuno-oncotherapy due to their tropism for dendritic cells (DCs), in which they induce endogenous antigen expression, hence, the ability of LVs to induce strong and long-lasting T-cell immunity (Ku *et al*, 2021d). Only non-integrative LVs can be used for these purposes. In non-integrative LVs, the catalytic site of the integrase is mutated and therefore the viral retrotranscribed DNA cannot integrate into the host chromosome and is maintained in the nucleus as a non-integrated episomal form (Ku *et al*, 2021d; Nemirov *et al*, 2023). The use of non-integrative LVs requires adjustment of the dose. Typically, the same immunogenicity efficacy is reached with 10 times more non-integrative than integrative LVs. LVs are non-replicative, non-cytopathic, self-inactivating, and only weakly inflammatory (Ku *et al*, 2021d; Lopez *et al*, 2022). With LVs, effective T-cell responses are induced at the lowest possible inflammatory response cost. The safety of LVs has been established in humans in phase I/IIa human immunodeficiency virus-1 therapeutic trial (TheraVectys-Clinical-Trial, 2019) in which a 5-year follow-up of cohorts of AIDS patients detected no serious adverse events, no genotoxicity, and no treatment-related safety issues, but induction of polyfunctional T-cell responses. In another phase I therapeutic study based on a non-integrative LV performed on patients with sarcoma and other solid tumors, no treatment-related adverse effects were reported. No genotoxicity was recorded and the induction of T-cell responses was detected and associated with a clinical benefit (Pollack *et al*, 2017; Somaiah *et al*, 2019).

Lentiviral vector particles are predominately pseudotyped with the glycoprotein from vesicular stomatitis virus (VSV-G), responsible for broad tropism for diverse cell types, notably DCs, the avoidance of LVs being targets of preexisting immunity in humans. Our laboratory has already established the preclinical proofs of concept and efficacy of prophylactic LV-based vaccination against West Nile virus (Iglesias *et al*, 2006), Japanese encephalitis virus (de Wispelaere *et al*, 2015; Garcia-Nicolas *et al*, 2017), Zika virus (Ku *et al*, 2020), SARS-CoV-2 (Ku *et al*, 2021a,c; Vesin *et al*, 2022), and *Mycobacterium tuberculosis* (Anna *et al*, 2022; Lopez *et al*, 2022). The efficacy of an LV-based oncotherapeutic vaccination was also established using an LV-encoding human telomerase (Adotevi *et al*, 2010). A single intramuscular (i.m.) injection of this LV vaccine-induced complete eradication of B16 melanoma tumors in 30% of mice and significantly reduced the tumor size in the remaining mice (Adotevi *et al*, 2010). In addition, a single i.m. administration of LV::OVA to mice bearing large OVA-expressing EG.7 tumors led to complete tumor eradication (Ku *et al*, 2021b). The LV::OVA-mediated protective effect was higher than that of the adenoviral-based "Ad5::OVA". The use of an LV, encoding inactivated HPV16 E7 antigen fused to calreticulin to enhance MHC-I antigen presentation, generated preliminary encouraging results in HPV-induced tumor eradication (Grasso *et al*, 2013).

Here, we generated a panel of non-integrative LVs encoding assortments of inactivated E6 and E7 antigens from HPV16 and HPV18 to enlarge the vaccine specificity. Furthermore, these antigens were designed in various permutations in case their differential positioning could impact their T-cell immunogenicity. We evaluated the efficacy of these LVs in immuno-oncotherapy in mice, engrafted with HPV-induced tumors. One of the generated vaccine candidates, "Lenti-HPV-07", was able to fully eradicate solid tumors in 100% of animals that correlated with polyfunctional $CD8^+$ T-cell immunity and a large potential to remodel the immune tumor infiltrates toward a pro-inflammatory microenvironment. Furthermore, Lenti-HPV-07 immunotherapy induced long-term immune protection, preventing tumor growth after a late rechallenge. Lenti-HPV-07 therapy also showed its full efficacy in a lung metastatic tumor model, correlated with the presence of $CD44^+$ $CD69^+$ $CD103^+$ $CXCR3^+$ resident memory $CD8^+$ T cells in the lung parenchyma. A synergistic effect of Lenti-HPV-07 and anti-programmed cell death protein (PD) 1 treatment was also demonstrated.

Results

Down-selection of Lenti-HPV-07 as an effective LV-based oncotherapeutic treatment

We designed an assortment of non-oncogenic, inactivated forms of E6 and E7 antigens from HPV16 and HPV18, ordered in various permutations, in four distinct LV vaccine candidates, namely "Lenti-HPV-07 to Lenti-HPV-10" (Fig 1A). The oncogenic properties of E6 and E7 were inactivated by removing their binding motifs to: (i) p53 tumor suppressor protein (Wieking *et al*, 2012; Hussain *et al*, 2021), (ii) chromodomain-helicase-DNA-binding protein 4 (CHD4) (Mills, 2017), (iii) retinoblastoma (Rb) tumor suppressor protein (Bellacchio & Paggi, 2013), and (iv) PDZ, named for the common structural domain shared by the post-synaptic density protein, drosophila disc large tumor suppressor, and zonula occludens-1 protein (Romero *et al*, 2011) (Fig EV1). We generated non-integrative, VSV-G pseudo-typed Lenti-HPV, each encoding one of the four distinct designed polyantigens, under the human β2-microglobulin promoter which can transcriptionally favor antigen expression by LV-transduced DCs and which induces higher antigen expression *in vivo* compared to cytomegalovirus promoter (Ku *et al*, 2021b). The human β2-microglobulin promoter contains elements regulated by pro-inflammatory cytokines which are upregulated during the DC activation. In addition, this promoter has minimal proximal enhancers, which improves the biosafety of the vector (Ku *et al*, 2021b). Using this promoter, the higher proportions of LV-transduced cells after i.m. immunization are detected in macrophages, conventional, and plasmacytoid DCs versus minutes proportions of lymphocytes (Ku *et al*, 2021b).

The antitumor efficacy of each Lenti-HPV was directly assessed in C57BL/6 mice subcutaneously (s.c.) engrafted with 1×10^6 syngeneic HPV-induced TC-1 cells ($n = 8$/group). Eleven days after tumor inoculation, mice were randomized and immunized by a single i.m. injection of individual Lenti-HPV. An LV encoding an irrelevant protein was used as a negative control LV ("Ctrl Lenti"). We detected a complete tumor regression in 100% of mice vaccinated with Lenti-HPV-07 and Lenti-HPV-10 (Fig 1B). In contrast, Lenti-

 © 2023 The Authors

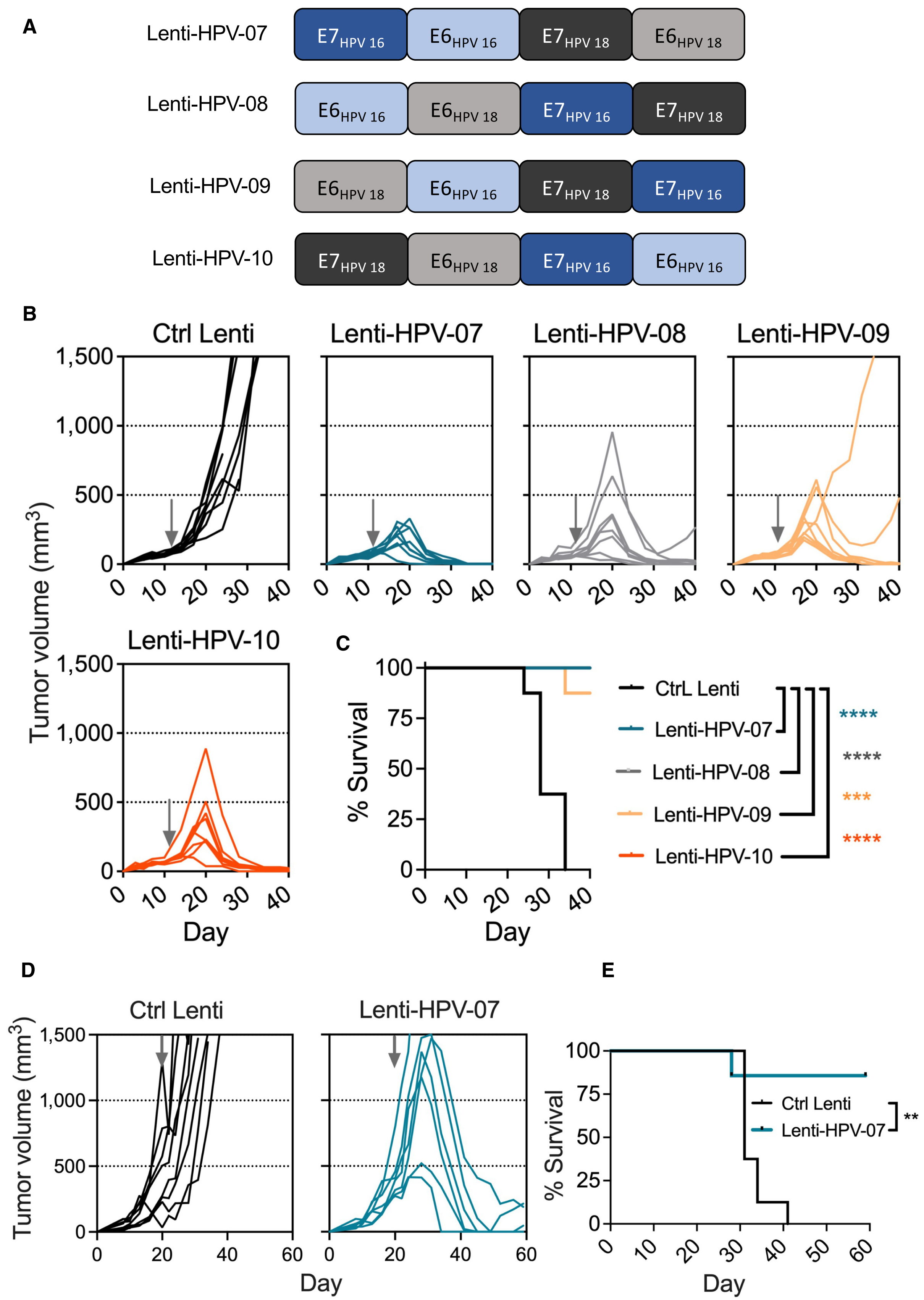
A
Lenti-HPV-07
E7HPV 16 E6HPV 16 E7HPV 18 E6HPV 18
Lenti-HPV-08
E6HPV 16 E6HPV 18 E7HPV 16 E7HPV 18
Lenti-HPV-09
E6HPV 18 E6HPV 16 E7HPV 18 E7HPV 16
Lenti-HPV-10
E7HPV 18 E6HPV 18 E7HPV 16 E6HPV 16
B
Ctrl Lenti
Lenti-HPV-07
Lenti-HPV-08
Lenti-HPV-09
Lenti-HPV-10
Tumor volume (mm3)
Day
C
% Survival
CtrL Lenti
Lenti-HPV-07
Lenti-HPV-08
Lenti-HPV-09
Lenti-HPV-10
****
****
***
****
Day
D
Ctrl Lenti
Lenti-HPV-07
Tumor volume (mm3)
Day
E
% Survival
Ctrl Lenti
Lenti-HPV-07
**
Day

Figure 1.

Figure 1. Therapeutic effect of vaccination in mice bearing HPV-induced tumors with various Lenti-HPV vectors.

A Four antigenic designs of non-oncogenic, inactivated forms of E6 and E7 antigens from HPV16 and HPV18 with distinct permutations, as encoded by non-integrative LV-based HPV therapeutic vaccine candidates.

B, C C57BL/6 mice ($n = 8$/group) were engrafted s.c. on the right flank with 1×10^6 TC-1 cells. At day 10 post-engraftment, when the tumor volume reached an average of 70 mm^3, mice were randomized. Vaccination was performed at day 11 by i.m. injection of 1×10^9 TU/mouse of individual Lenti-HPV vectors. Tumor-bearing control animals received Ctrl Lenti. (B) Spaghetti plots of tumor size over time. The gray arrows indicate the time point at which the vaccine was injected. (C) Survival curve of the animals.

D, E C57BL/6 mice ($n = 7$–8/group) were engrafted s.c. on the right flank with 1×10^6 TC-1 cells. At day 20, when the tumor volume reached an average of 450 mm^3, mice were randomized. Vaccination was performed at day 20 by i.m. injection of 1×10^9 TU/mouse of Lenti-HPV-07. Tumor-bearing control animals received Ctrl Lenti. (D) Spaghetti plots of tumor size over time. The gray arrows indicate the time point at which the vaccine was injected. (E) Survival curve of the animals.

Data information: Statistical significance was determined by Log-rank Mantel-Cox tests (ns, not significant, $**P \leq 0.01$, $***P \leq 0.001$, $****P \leq 0.0001$. Mice were sacrificed when the tumor volume reached 1,500 mm^3, defined as humane endpoints. The experiments shown is representative of two independent experiments. The experiment shown in (D, E) has been performed once.
Source data are available online for this figure.

HPV-08 and Lenti-HPV-09 induced tumor regression in only 87.5% (7/8) or 75% (6/8) of mice, respectively. Furthermore, tumor cells grew much less in Lenti-HPV-07-treated mice than in Lenti-HPV-10-treated mice (Fig 1B). All Ctrl Lenti-injected mice reached the humane endpoint before day 35, whereas the survival of the Lenti-HPV-immunized groups was significantly prolonged (Fig 1C).

To evaluate the Lenti-HPV-07 efficacy in a more aggressive model, we treated mice bearing tumors with an average volume of 450 mm^3 with a single i.m. injection of 1×10^9 TU of Lenti-HPV-07 or Ctrl Lenti (Fig 1D and E). Six of 7 Lenti-HPV-07-treated mice were able to fully eradicate such large tumors or to induce a significant decrease in tumor growth (Fig 1D). One mouse with the largest tumor in the Lenti-HPV-07 group had to be killed at day 28 post-tumor implantation because of ethical consideration, yet we cannot exclude that this individual would have been able to control the tumor growth. Accordingly, the survival of Lenti-HPV-07-treated mice was significantly increased compared to the Ctrl Lenti group (Fig 1E). Therefore, Lenti-HPV-07 induced full tumor regression in all animals that were ethically able to be maintained alive because of the large tumor size.

Altogether, the LV platform is effective for use in solid tumor immunotherapy, and among the LV constructs tested here, Lenti-HPV-07 is the most effective.

Features of systemic Lenti-HPV-07-induced T-cell immunity

We assessed the induced T-cell immunogenicity by injecting C57BL/6 mice i.m. at day 0 with 1×10^8 or 1×10^9 TU of Ctrl Lenti or Lenti-HPV-07. At day 14, splenocytes were used in IFN-γ ELISPOT after *in vitro* stimulation with pooled peptides derived from $E6_{HPV16}$, $E7_{HPV16}$, $E6_{HPV18}$, or $E7_{HPV18}$ (Fig 2A). Lenti-HPV-07 induced IFN-γ T-cell responses notably against $E7_{HPV16}$ in C57BL/6 H-2^b mice. Weaker responses were also recorded against $E6_{HPV16}$, $E6_{HPV18}$ and $E7_{HPV18}$. We also comparatively analyzed IFN-γ T-cell responses against $E6_{HPV16}$, $E6_{HPV18}$, $E6_{HPV18}$, and $E7_{HPV18}$ antigens after immunization of C57BL/6 mice by Lenti-HPV-07, Lenti-HPV-08, Lenti-HPV-09 or Lenti-HPV-10 vectors. The T-cell immunogenicity profiles of the four Lenti-HPVs were very similar (Fig EV2A), consistent with their notable antitumor potential (Fig 1B and C). Indeed, the slight differences between the antitumor activity of the four vectors may have originated from a few outliers without significant impact on the tumor eradication rate or percentages of survival (Fig 1B and C). Importantly, the comparable immunogenicity of the four Lenti-HPVs against the selected HPV antigens clearly showed the lack of antigen positioning effect on T-cell immunogenicity of antigens in various Lenti-HPVs.

To gain further insight into the quality of the T-cell responses, splenocytes from mice injected with Ctrl Lenti or Lenti-HPV-07 were left untreated or stimulated *in vitro* with a mixture of $E7_{HPV16}$: ETTDPDRAHYNIVTF and $E7_{HPV16}$:PDRAHYNIVTFCCKC peptides, both containing the immunodominant RAHYNIVTF T-cell epitope (Feltkamp *et al*, 1993) and were analyzed by intracellular staining (ICS) (Fig 2B). In Lenti-HPV-07-vaccinated mice, stimulation with these $E7_{HPV16}$ peptides detected functional $CD8^+$ T-cell effectors, mainly distributed among IFN-γ^+ (single positive), TNF-α^+ IFN-γ^+ or IL-2^+ IFN-γ^+ (double positive), and IL-2^+ TNF-α^+ IFN-γ^+ (triple positive) subsets (Fig 2C). Most IFN-γ^+ $CD8^+$ T cells also expressed the surface CD107a degranulating marker, showing the effector properties of these T cells (Fig 2D). Importantly, Lenti-HPV-07 was also immunogenic in "HHD-DR1" mice which are fully devoid of murine MHC molecules and express only those from human leukocyte antigen (HLA) 02.01, DRA01.01, and DRB1.01.01 alleles, widely expressed in human populations (Altmann *et al*, 1995; Pascolo *et al*, 1997; Madsen *et al*, 1999; Pajot *et al*, 2004). The main T-cell response in Lenti-HPV-07-vaccinated HHD-DR1 mice was oriented against $E6_{HPV18}$ (Fig 2E). Altogether, the immunogenicity of Lenti-HPV-07: (i) against $E7_{HPV16}$ and also at a lesser extent against $E6_{HPV18}$ and $E7_{HPV18}$ in C57BL/6 (H-2^b) mice (Fig 2A), and (ii) against $E6_{HPV18}$ in MHC-humanized HHD-DR1 mice (Fig 2E), reflected well the accurate expression of these various antigens in the host antigen presenting cells transduced by Lenti-HPV-07. We also emphasize that the four antigens are expressed as a single polyprotein and that the four segments can only be expressed with the same intensity. The differences in the profile of Lenti-HPV-07 immunogenicity in these distinct animal models are linked to the selection of different T-cell epitopes by diverse MHC molecules from various H-2 or HLA haplotypes for presentation to T cells.

To determine the phenotype of T cells responsible for tumor eradication, tumor-bearing C57BL/6 mice, vaccinated with Lenti-HPV-07 at day 14, received i.p. a control Ig or depleting anti-CD4 or anti-CD8 monoclonal antibodies (mAbs). The resulting efficient *in vivo* T-subset depletion (Fig EV2B), showed that the main Lenti-HPV-07-induced effectors involved in tumor elimination were $CD8^+$ T cells and that $CD4^+$ T cells made no sizeable contribution (Fig 2F).

 © 2023 The Authors

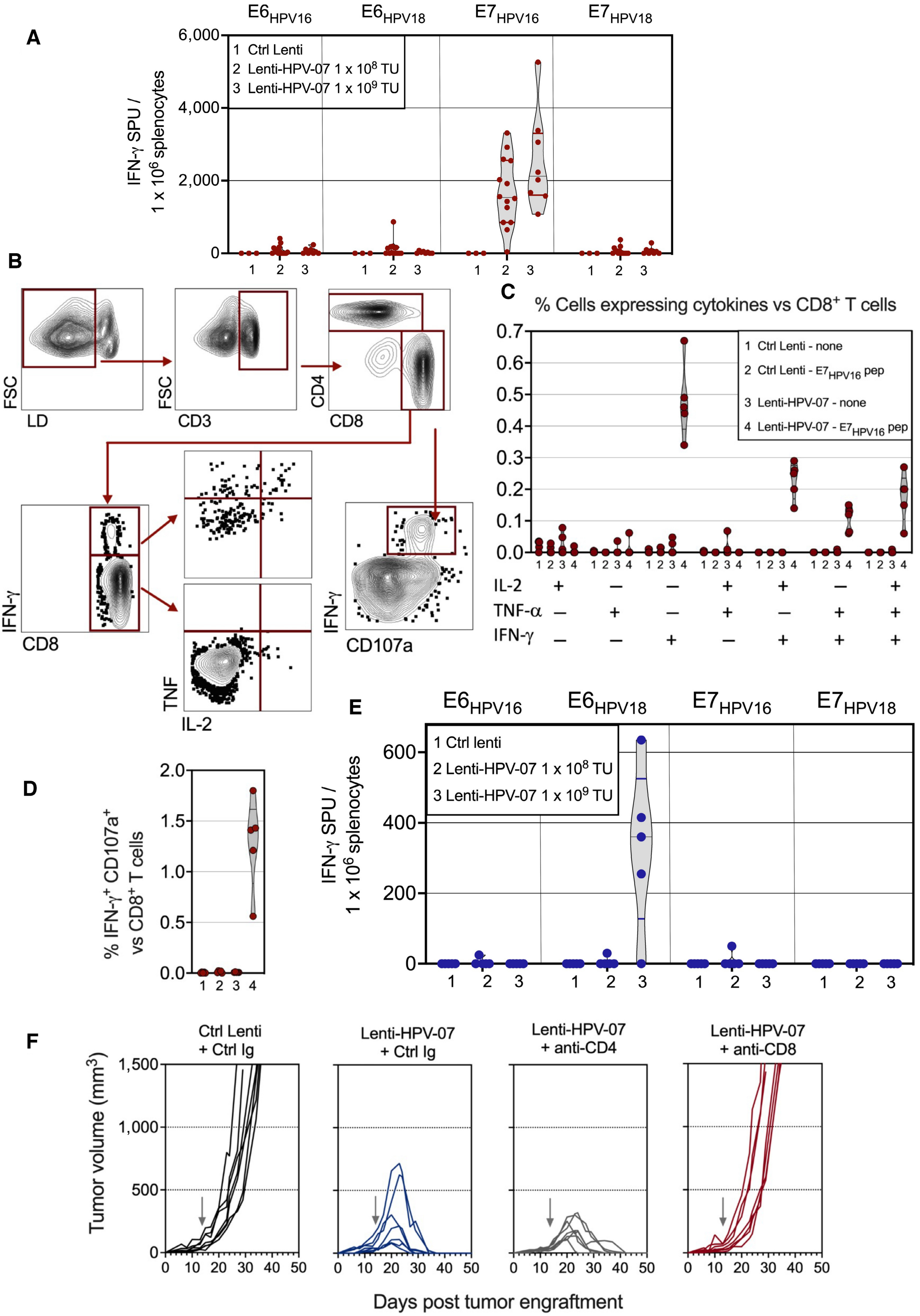
A
E6HPV16
E6HPV18
E7HPV16
E7HPV18
1 Ctrl Lenti
2 Lenti-HPV-07 1 x 10^8 TU
3 Lenti-HPV-07 1 x 10^9 TU
IFN-γ SPU / 1 x 10^6 splenocytes
B
FSC
LD
CD3
CD4
CD8
IFN-γ
TNF
IL-2
CD107a
C
% Cells expressing cytokines vs CD8+ T cells
1 Ctrl Lenti - none
2 Ctrl Lenti - E7HPV16 pep
3 Lenti-HPV-07 - none
4 Lenti-HPV-07 - E7HPV16 pep
IL-2
TNF-α
IFN-γ
D
% IFN-γ+ CD107a+ vs CD8+ T cells
E
1 Ctrl lenti
F
Ctrl Lenti + Ctrl Ig
Lenti-HPV-07 + Ctrl Ig
Lenti-HPV-07 + anti-CD4
Lenti-HPV-07 + anti-CD8
Tumor volume (mm3)
Days post tumor engraftment

Figure 2.

Figure 2. Characterization of Lenti-HPV-07-induced antitumor T-cell effectors.

A C57BL/6 mice received i.m.: (1) 1×10^9 TU of Ctrl Lenti (n = 3), (2) 1×10^8 TU (n = 14) of Lenti-HPV-07, or (3) 1×10^9 TU (n = 8) of Lenti-HPV-07. At day 14 post injection, splenocytes from individual mice were studied by IFN-γ ELISPOT after *in vitro* stimulation with pools of synthetic 15-mers spanning the sequence of $E6_{HPV16}$, $E6_{HPV18}$, $E7_{HPV16}$, or $E7_{HPV18}$.

B–D T-cell cytokine responses of splenocytes from Ctrl Lenti- or Lenti-HPV-07-injected mice (n = 5) were also studied by ICS, with or without stimulation with a mixture of $E7_{HPV16}$:ETTDPDRAHYNIVTF and $E7_{HPV16}$:PDRAHYNIVTFCCKC peptides, both containing the RAHY**N**IVT**F** H-2D^b-restricted T-cell epitope. Bold characters represent H-2D^b anchor residues. (B) Cytometric gating strategy carried out on IL-2-, TNF-α-, and IFN-γ-producing CD8$^+$ T cells. (C) Recapitulative frequencies of each (poly) functional cell subsets and (D) degranulation activity of the IFN-γ-producing CD8$^+$ T cells, assessed by surface CD107a staining.

E HHD-DR1 mice received i.m.: (1) 1×10^9 TU of a Ctrl Lenti (n = 4), (2) 1×10^8 TU (n = 4) Lenti-HPV-07, or (3) 1×10^9 TU (n = 5) of Lenti-HPV-07 and their splenocytes were studied by IFN-γ ELISPOT, as described in (A).

F Mice (n = 7/group) engrafted s.c. with 1×10^6 tumor cells were injected at day 14 with 1×10^9 TU of Ctrl Lenti or Lenti-HPV-07 and then treated with a Ctrl Ig or anti-CD4 or anti-CD8 mAbs, as detailed in Material and Methods. Spaghetti plots of tumor growth in the various group. The gray arrows indicate the time point at which Lenti-HPV-07 was injected.

Data information: The experiments shown are representative of at least two independent experiments.
Source data are available online for this figure.

No HPV-specific IFN-γ T-cell response was detected in C57BL/6 mice bearing tumors and treated with the Ctrl Lenti, indicating that the TC-1 tumor itself did not induce systemic E6/E7-specific CD8$^+$ T cells (Appendix Fig S1).

Profound remodeling of intra-tumoral immune infiltrates in Lenti-HPV-07-vaccinated mice

Characterization of the intra-tumoral infiltrates at day 11 post-vaccination, that is, during the tumor regression phase, showed: (i) a large increase in the percentage of CD45$^+$ hematopoietic cells (Fig 3A), and (ii) a significant increase in the percentages of CD8$^+$ T cells within the CD45$^+$ population, and (iii) a significant decrease in the percentages of CD4$^+$ T cells within the CD45$^+$ population (Fig 3B). The decrease in the CD4$^+$ T-cell proportions most likely resulted from the increased CD8$^+$ T cell proportions (Fig 3B) and/or reduction in the proportions of CD25$^+$ FoxP3$^+$ regulatory T cells (Tregs) within the CD4$^+$ T subset (Appendix Fig S2A). Immunohistochemistry analysis confirmed a preferential expansion and/or recruitment of CD8$^+$—but not CD4$^+$—T cells in the tumors from Lenti-HPV-07-treated mice relative to their Ctrl Lenti-treated counterparts (Figs 3C and EV3). Homogeneous intra-tumoral distribution of CD8$^+$ T cells was observed in Lenti-HPV-07-treated mice, suggesting that the entire tumor mass was accessible to the effector T cells. Within the CD8$^+$ T-cell subset, there was an increase in the percentage of CD44$^+$ CD69$^+$ CD103$^+$ resident memory T cells (Trm) (Fig 3D), a hallmark of effective and long-lasting protective potential (Masopust & Soerens, 2019). There was also an increase in the percentage of killer-cell lectin-like receptor G1 (KLRG1)$^+$ cells observed within the CD44^{dim} CD69$^-$ CD8$^+$ T-cell compartment (Fig 3E and F), characteristic of terminally exhausted T cells, most likely resulting from chronic TCR stimulation and/or checkpoint protein signaling, at this dynamic stage of T-cell mediated tumor regression.

At this time point post-vaccination, the vast majority of CD8$^+$ T cells showed a PD1$^+$ and T cell immunoglobulin domain and mucin domain (Tim)-3$^+$ phenotype (Fig 3G). Most of these cells also expressed the transcription factor T cell factor-1 (TCF-1) (Zhao *et al*, 2022) (Fig 3H). Such "stem-like" T cells are usually associated with the memory phenotype and characterized by their activation status and their survival transcriptional and epigenetic programs (Hiam-Galvez *et al*, 2021), notably in HPV-induced cancers (Eberhardt *et al*, 2021). Principal component analysis (PCA) of the above-mentioned cytometric data showed a clear-cut distinction between the intra-tumoral T cells from Ctrl Lenti- and Lenti-HPV-07-treated mice (Fig 3I).

As mentioned above, we also observed a significant decrease in the frequency of intra-tumoral CD4$^+$ CD25$^+$ FoxP3$^+$ regulatory T cells (Tregs) in Lenti-HPV-07-vaccinated mice (Appendix Fig S2A). In net contrast to the major remodeling of the intra-tumoral T-cell compartment, the frequency of intra-tumoral B220$^+$ B cells was similar in Ctrl Lenti- and Lenti-HPV-07-treated mice (Appendix Fig S2B). There was also a significant increase in the proportions of natural killer (NK) cells in the regressing tumors (Fig 4A). However, an efficient NK-cell depletion by anti-NK1.1 mAb treatment in tumor-bearing and Lenti-HPV-07-treated mice had no effect on the immune control of tumor growth (Fig EV4A–C).

In the Lenti-HPV-07-vaccinated mice, we also observed an increase in the frequency of intra-tumoral CD11b$^+$ Ly6G$^+$ granulocytic cells (Fig 4B). Without a functional assay, it was difficult to determine whether these cells were polymorphonuclear (PMN) or PMN-myeloid-derived suppressor cells (PMN–MDSCs; Douguet *et al*, 2018). Only in Lenti-HPV-07-vaccinated mice was an intra-tumoral cell population with a CD11b$^+$ F4/80^{hi} CD11c$^+$ MHC-II$^+$ phenotype, containing a CD24$^+$ cell subtype, detected (Fig 4C), which may correspond to a previously described monocyte-derived macrophage/cDC2 subset, with migratory and cross-presentation properties, that is enriched in the tumor microenvironment (TME) (Sheng *et al*, 2017). PCA projection plots of the abovementioned innate immune cell characteristics showed a net distinction between the intra-tumoral innate immune cells from Ctrl Lenti- and Lenti-HPV-07-treated mice (Fig 4D). We also observed an increased ratio of total T cells versus total CD11b$^+$ innate cells in the regressing tumors of Lenti-HPV-07-vaccinated mice (Fig 4E). Strong upregulation of PD1 ligand (PD-L1) was detected on TC-1 cells in the regressing tumors of Lenti-HPV-07-treated mice, in contrast to the tumor cells in growing tumors of the Ctrl Lenti-treated controls (Fig 4F).

These results show that a profound remodeling of intra-tumoral immune infiltrates correlated with efficient tumor regression in Lenti-HPV-07-vaccinated mice.

Intra-tumoral inflammatory status of Lenti-HPV-07-treated mice

We further compared the inflammatory status of the TME in Ctrl Lenti- or Lenti-HPV-07-treated mice, by performing qRT–PCR on RNA extracted from the total tumors recovered at day 9 post-

 © 2023 The Authors

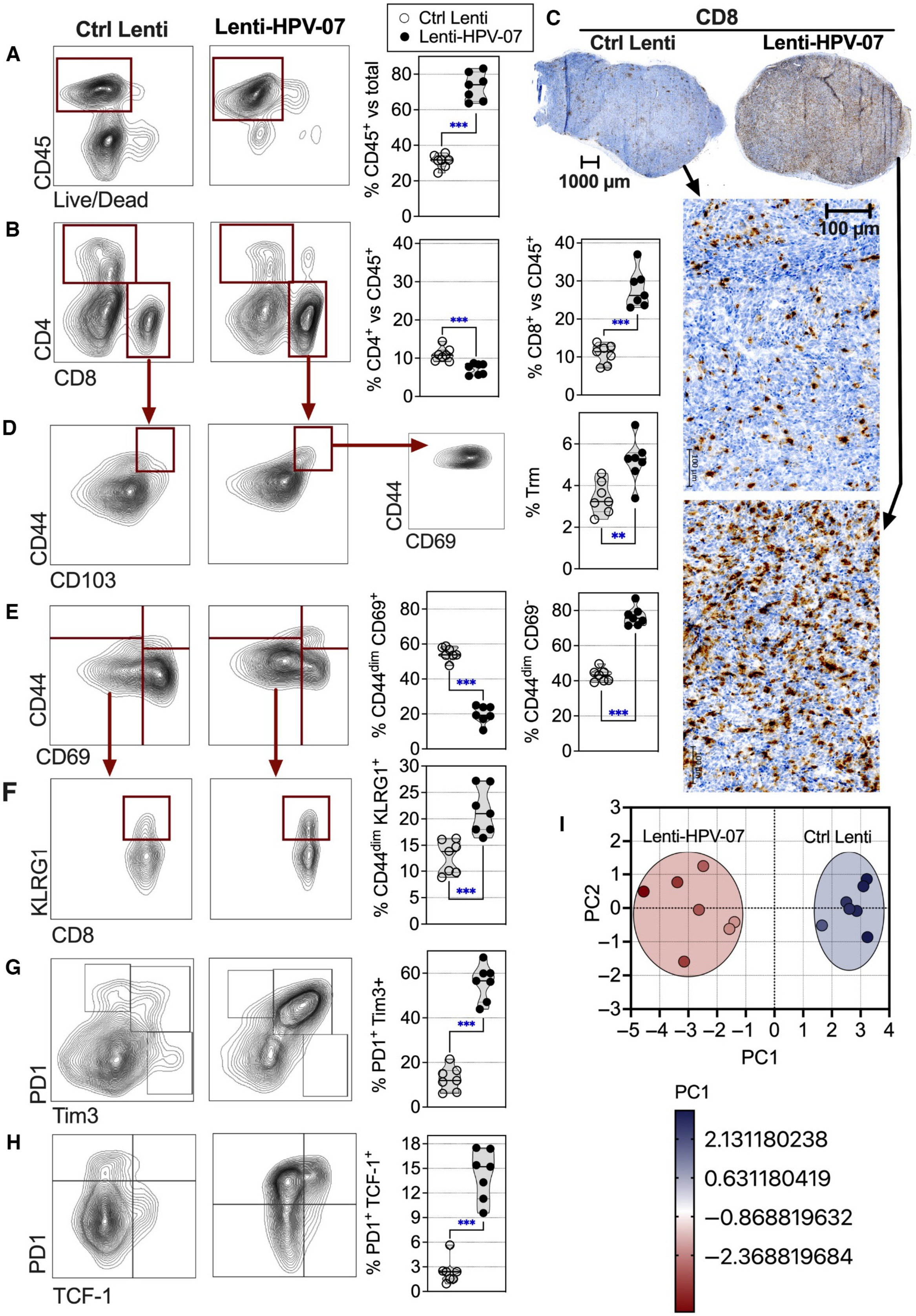
Ctrl Lenti
Lenti-HPV-07
A
B
C
D
E
F
G
H
I
CD45
Live/Dead
% CD45+ vs total
CD4
CD8
% CD4+ vs CD45+
% CD8+ vs CD45+
CD8
1000 µm
100 µm
CD44
CD103
CD69
% Trm
% CD44dim CD69+
% CD44dim CD69-
KLRG1
% CD44dim KLRG1+
PD1
Tim3
% PD1+ Tim3+
TCF-1
% PD1+ TCF-1+
PC1
PC2
2.131180238
0.631180419
−0.868819632
−2.368819684

Figure 3.

◀ **Figure 3. Tumor infiltrating T cells in Lenti-HPV-07-vaccinated mice.**

C57BL/6 mice were engrafted s.c. with 1×10^6 TC-1 cells and injected with 1×10^9 TU Lenti Ctrl or Lenti-HPV-07 ($n = 7$/group). Tumors were studied on day 11 post-vaccination.

A, B Representative dot blots of tumor infiltrating T cells, studied by cytometry.

C Representative tumor infiltrating T cells, studied by anti-CD8 immunohistochemistry.

D–H Representative dot blots of tumor infiltrating T cells, studied by cytometry. For cytometry data, the percentage of each subset was compared between the two groups and statistical significance was determined using two-tailed unpaired *t*-tests (**$P \leq 0.01$, ***$P \leq 0.001$).

I PCA projection plot of the parameters studied in (A, B, D–H). Differential characteristics of intra-tumoral T cells between Ctrl Lenti- and Lenti-HPV-07-treated groups. PC1 of 10 variables, shown in (A, B, D–H) split the samples by treatment. Proportion of variance for PC1 = 87.71%.

Data information: For cytometry data, the percentage of each subset was compared between the two groups and statistical significance was determined using two-tailed unpaired *t*-tests (**$P \leq 0.01$, ***$P \leq 0.001$). The experiments shown are representative of at least two independent experiments.
Source data are available online for this figure.

treatment, that is, at the beginning of tumor regression. In accordance with the intra-tumoral immune cell composition (Figs 3 and 4), the TME from Lenti-HPV-07-vaccinated mice showed a stronger inflammatory signature than that from the growing tumors of Ctrl Lenti-treated mice (Fig 5A). The immunosuppressive transforming growth factor (TGF)-β and IL-10 were also upregulated in the TME from Lenti-HPV-07-vaccinated mice, most likely as part of a typical attempt of the immune system prone to resolve inflammation and limit inflammation-induced tissue damage (Sanjabi *et al*, 2009). The expression of Th1 cytokines, that is, IFN-γ, TNF-α, IL-2, was higher in the TME of Lenti-HPV-07-vaccinated mice (Fig 5A) in a correlative manner (Fig 5B). The expression of the innate cytokines IL-6, IL-12p40, and IL-18 was also significantly and correlatively higher. The expression of CCL3, CCL4, and CCL5 chemokines, also higher, may be major determinants of tumor infiltration by DCs and NKs (Allen *et al*, 2018). The increase in IFN-γ-induced CXCL9 and CXCL10 expression, also observed in the tumors of vaccinated mice, may be associated with intra-tumoral recruitment of activated $CD8^+$ T cells through an interaction via CXCR3. Up-regulation of ICAM-1 may also favor T-cell adhesion and is critical for solid tumor elimination, as recently described (Larson *et al*, 2022). Overexpression of CXCL5 can chemo-attract T cells and neutrophils and contribute to tumor angiogenesis (Zhang *et al*, 2020). The observed increase in the CCL19 expression may promote IFN-γ-dependent anti-tumor defense by inducing the homing of $CCR7^+$ T cells and DCs (Schaerli & Moser, 2005). Myeloid-associated (M-CSF, GM-CSF, and MIF) or fibroblast growth factor (FGF-2, FGF-7), vascularization (VEGF-C, VCAM-1), or extra-cellular matrix components (MMP-1, MMP-9, and collagen, type 1 alpha 1) were little or unaffected by vaccination, suggesting tumor regression driven by Th1 inflammation without other global modifications of the TME (Hong *et al*, 2022).

Efficacy of Lenti-HPV-07 therapy against pulmonary metastases

The lung is the most frequent anatomical site of metastatic spreading of solid tumors, including HPV-induced tumors (van Boerdonk *et al*, 2013). We thus evaluated the capacity of Lenti-HPV-07 to inhibit tumor growth in the pulmonary mucosal site. We generated a TC-1 cell line stably expressing the nano-luciferase reporter gene (nLuc-TC-1) to apply a bioluminescence-based live animal imaging which is one of the most sensitive and quantitative assessment of tumor burden over time and which allows longitudinal tracking of metastases in individual animals. Such an analysis by histological method would have weak sensitivity as histological sections cannot cover the totality of the organs and would have required injection of a much larger number of tumor cells per animal. Cytometric detection would have been a sensitive alternative method but would have required the introduction of a fluorochrome reporter to TC-1 cells, which would have generated the same questions about the possible immunogenicity of the reporter. To the best of our knowledge, there are no data in the literature showing any sign of immunogenicity of the small nLuc (10 kDa) enzyme which behaves as a non-immunogenic protein in our hands. In any case, we compared the extent of metastasis with the same nLuc-TC-1 cell line in groups of mice treated with Ctrl Lenti or Lenti-HPV-07 in which possible immunogenicity of the reporter would be the same and could not be a confounding factor.

After intravenous (i.v.) injection of 1.5×10^4 nLuc-TC-1 cells, C57BL/6 mice readily developed pulmonary metastatic foci, in accordance with previous observations (Ji *et al*, 1998). Five days after tumor injection, mice received a single i.m. injection of 1×10^9 TU of Ctrl Lenti or Lenti-HPV-07 ($n = 12$/group). Longitudinal tumor outgrowth was followed in live animals by bioluminescence imaging (Fig 6A). All Lenti-HPV-07-treated mice were cured at day 22, whereas pulmonary metastatic foci continued to grow in the Ctrl Lenti group. Largely statistically differences were observed between the bioluminescence signal in the two groups on days 18 and 22 (Fig 6B and C).

Lung T-cell status was also assessed in such mice at day 15. An i.v. PE-anti-CD45 mAb injection was given to the mice 3 min before sacrifice to distinguish the lung parenchymal ($CD45_{i.v.-}$) from vascular ($CD45_{i.v.+}$) $CD8^+$ T cells (Anderson *et al*, 2014) by cytometry (Fig 6D). Compared with the naïve control or Lenti Ctrl-treated mice, the Lenti-HPV-07-treated mice had no higher percentages of $CD8^+$ T cells in their lung parenchyma (Fig 6E). However, in the latter group, the frequencies of $PD1^+$ Tim^+ activated $CD8^+$ T cells and those of $CD44^+$ $CD69^+$ $CD103^+$ $CXCR3^+$ Trm among the parenchymal $CD8^+$ T cells were significantly increased (Fig 6F and G). No such differences were observed in the parenchymal $CD4^+$ T cells. At day 28, no more differences were observable between the lung T-cell composition of the Ctrl Lenti- and Lenti-HPV-07-treated groups.

Therefore, in the Lenti-HPV-07-treated mice, the presence of lung-activated and Trm $CD8^+$ T cells was correlated with the eradication of pulmonary metastatic tumors established following hematogenous spread.

Lenti-HPV-07 therapy protects mice bearing large tumors and induces long-term antitumor immune protection

We investigated the capacity of Lenti-HPV-07 to protect mice bearing larger tumors (Fig 7A). By day 18 after s.c. tumor engraftment at the

 © 2023 The Authors

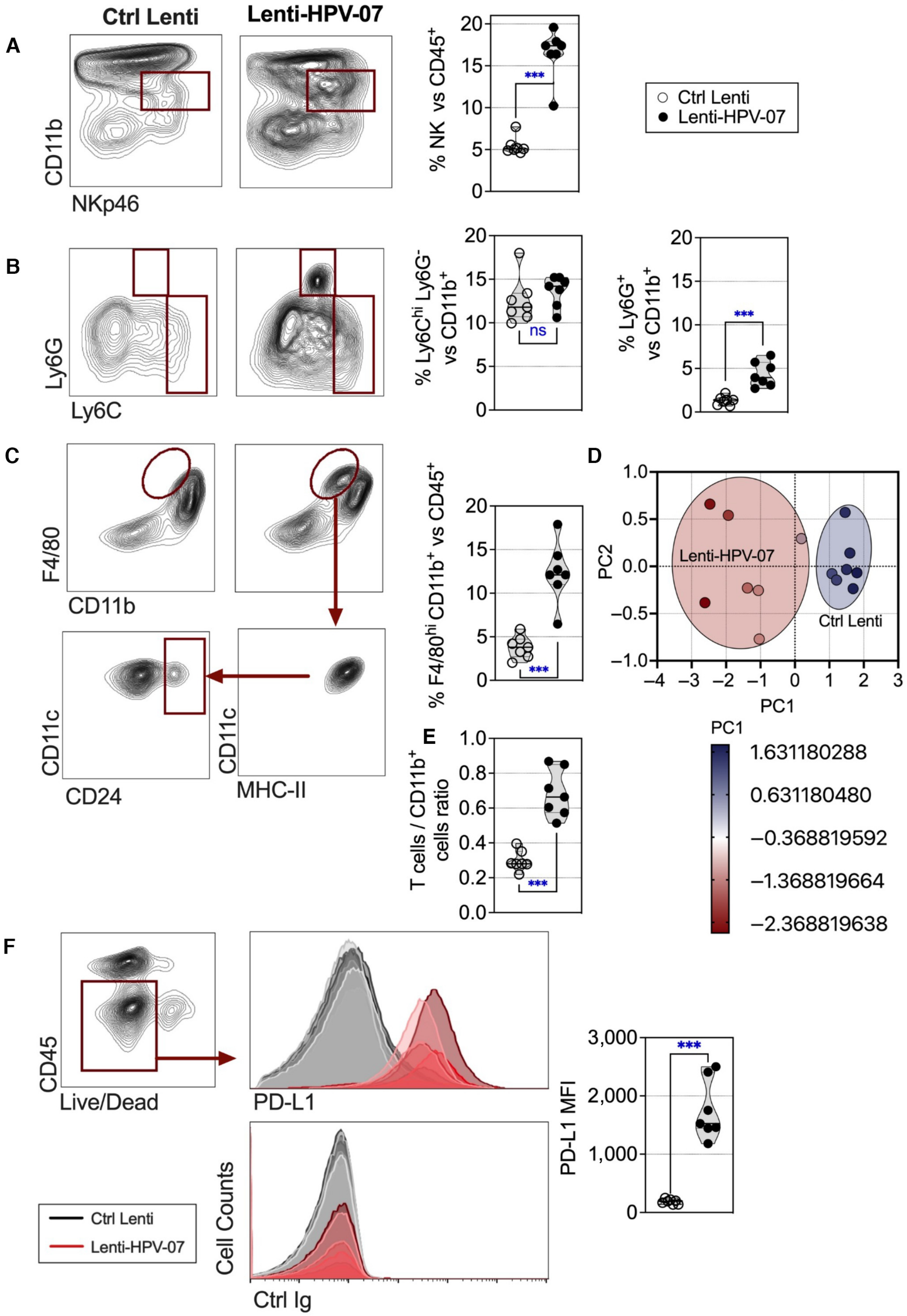
A
Ctrl Lenti
Lenti-HPV-07
CD11b
NKp46
% NK vs CD45+
***
Ctrl Lenti
Lenti-HPV-07
B
Ly6G
Ly6C
% Ly6Chi Ly6G- vs CD11b+
ns
% Ly6G+ vs CD11b+
***
C
F4/80
CD11b
CD11c
CD24
CD11c
MHC-II
% F4/80hi CD11b+ vs CD45+
***
D
Lenti-HPV-07
Ctrl Lenti
PC1
PC2
1.631180288
0.631180480
−0.368819592
−1.368819664
−2.368819638
E
T cells / CD11b+ cells ratio
***
F
CD45
Live/Dead
PD-L1
Cell Counts
Ctrl Ig
Ctrl Lenti
Lenti-HPV-07
PD-L1 MFI
***

Figure 4.

Figure 4. Features of tumor cells and tumor infiltrating innate immune cells in Lenti-HPV-07-vaccinated mice.

A–C The tumor-engrafted and vaccinated mice are those detailed in Fig 3 (n = 7/group). Tumor infiltrating innate immune cells in Lenti Ctrl- or Lenti-HPV-07-injected mice were studied on day 11 post-vaccination by cytometry. Representative blots for various innate cells are shown. The percentage of each subset was compared between the two groups and statistical significance determined using two-tailed unpaired t tests (ns: non-significant, ***$P \leq 0.001$).

D PCA projection plot of the parameters studied in (A–C). Differential characteristics of intra-tumoral innate immune cells between Ctrl Lenti- and Lenti-HPV-07-treated groups. PC1 of three variables, shown in (A–C), split the samples by treatment. Proportion of variance for PC1 = 93.48%.

E Ratio of intra-tumoral T cells vs $CD11b^+$ cells. Statistical significance was determined using two-tailed unpaired t tests (***$P \leq 0.001$).

F Comparative expression of PD-L1 at the surface of FSC^{hi} $CD45^-$ tumor cells, shown as overlayed cytometric histograms for individual mice from the two groups. Statistical significance was determined using two-tailed unpaired t tests (***$P \leq 0.001$).

Data information: The experiments shown are representative of at least two independent experiments.
Source data are available online for this figure.

right flank, tumors had reached ≈ 200 mm^3. All Lenti-HPV-07-vaccinated individuals experienced complete tumor regression within 40 days (Fig 7B), establishing the ability of Lenti-HPV-07 to induce full immune protection also against well-established tumors.

In these settings, we then determined whether the cured mice developed a long-term protective immune memory. To mimic tumor relapse, cured mice were re-challenged s.c. with 1×10^6 tumor cells in the opposite flank at day 120 after the first tumor engraftment, and maintained without any treatment (Fig 7A). A group of naïve mice received the same tumor cell suspension, as a positive control of tumor growth. All naïve control mice developed tumors, whereas all initially cured mice were protected from a tumor re-challenge and were still alive 145 days after the initial tumor engraftment (Fig 7C).

In another experiment, at day 286 after the initial tumor engraftment which corresponded to day 196 after tumor re-challenge, Lenti-HPV-treated and cured mice (n = 5/group) received an i.v. instillation of an adjuvant-free mixture of $E7_{HPV16}$:ETTDPDRAHYNIVTF and $E7_{HPV16}$:PDRAHYNIVTFCCKC peptides, both containing the immunodominant RAHYNIVTF T-cell epitope (Fig 7D). This method has been reported to awaken antigen-specific memory T cells without triggering primary T cells (Schenkel *et al*, 2013, 2014). Naïve controls received the same i.v. $E7_{HPV16}$ peptide treatment. Forty-eight hours after the peptide injection, splenocytes were stimulated *in vitro* with the homologous or a negative control peptide and studied by IFN-γ ICS and surface marker labeling (Fig 7E). The presence of long-lived antigen-specific memory T splenocytes was readily detectable by this approach in 2 of 5 mice (Fig 7E left bottom, F) and, to a lesser extent, in two other mice in the group of cured mice. No $IFN\text{-}\gamma^+$ $CD8^+$ T cells were detected in the negative control group that received the adjuvant-free peptides alone (Fig 7F). The majority of the responding cells in the cured mice were $CD44^+$, $CD62L^-$, $CD127^+$, and $KLRG1^-$, known as memory precursor cells (Ku *et al*, 2021b).

These observations show that a single Lenti-HPV-07 injection promoted antitumor immune memory, which efficiently shaped long-term T-cell responses to face tumor relapse new challenges.

Lenti-HPV-07 vaccination acts synergistically with anti-PD1 immunotherapy

We also investigated the potential synergy between a suboptimal dose of Lenti-HPV-07 vaccination and anti-PD1 therapy. Since the dose of 1×10^9 TU, as used in previous experiments, induced complete tumor eradication in 100% of the mice (Fig 1), to evaluate the possible beneficial effect of the additional anti-PD1 mAb treatment, we used the suboptimal dose of 1×10^8 TU, which is far from being effective alone. We designed two different regimens of anti-PD1 administration. First, we injected anti-PD1 mAb and Lenti-HPV-07 on the same day (Fig EV5). The combinatory therapy appeared to abrogate the beneficial effect of Lenti-HPV-07, as only 1 of 10 mice experienced complete tumor regression with this combinatory treatment, whereas 5 of 10 mice showed tumor eradication with suboptimal vaccination alone (Fig EV5). As Lenti-HPV-07 requires a few days to induce antigen expression and effector T-cell induction, simultaneous administration of anti-PD1 may inhibit the activation of antigen-specific T-cells. Therefore, in a second protocol, we began the anti-PD1 treatment 4 days after the Lenti-HPV-07 injection (Fig 8A). In this protocol, the suboptimal dose of 1×10^8 TU of Lenti-HPV-07, which induced an insufficient antitumor T-cell response, acted synergistically with anti-PD1 therapy to increase the tumor regression rate. Six of 14 mice achieved complete tumor regression and two others showed partial tumor regression in which tumor volume decreased by 67%. Then the tumor relapsed 6–7 days after the end of anti-PD1 treatment, highlighting the need for repeated injections of anti-PD1 until the tumor has completely disappeared. Only 3 of 12 mice treated with the suboptimal dose of Lenti-HPV-07 alone showed partial tumor regression (Fig 8B and C). The progression-free survival time doubled in mice that received the combination of Lenti-HPV07 and anti-PD1 compared to the mice treated with the vaccine alone (Fig 8D). Anti-PD1 alone was effective in only one mouse of 10. Accordingly, survival was significantly increased in the group with combinatory treatment (Fig 8E). Because therapy with anti-PD1 mAb alone had practically no effect on tumor eradication and the impact of combination therapy was higher than that of Lenti-HPV-07 alone (Fig 8B, C and E), the effect of combination therapy was synergistic rather than additive.

In contrast to the high efficacy of the optimal dose of 1×10^9 TU of Lenti-HPV-07 on very large tumors with an average volume of 450 mm^3 (Fig 1D and E), the combination of a suboptimal dose of 1×10^8 TU of Lenti-HPV-07 with anti-PD1 mAb therapy was not effective on very large tumors (Appendix Fig S3).

Therefore, a synergistic anti-tumor effect can be achieved when Lenti-HPV-07 is combined with delayed anti-PD1 checkpoint inhibitory treatment only in the case of small- or medium-sized tumors. The optimal dose of Lenti-HPV-07 alone remains the best therapeutic option for large tumors.

Discussion

We developed an onco-therapeutic anti-HPV vaccine candidate based on an LV platform that induced full eradication of HPV-induced

 © 2023 The Authors

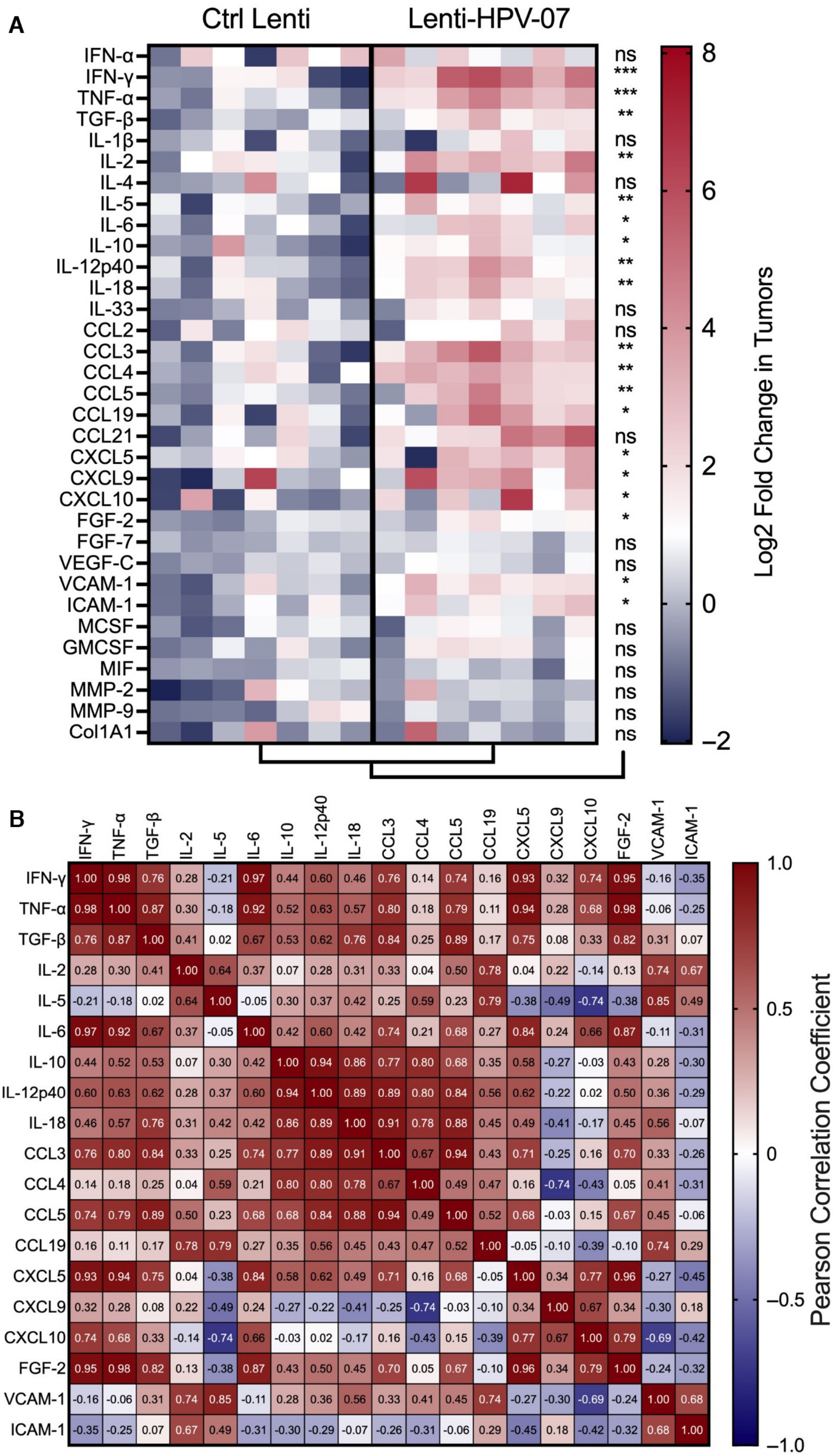
A
Ctrl Lenti
Lenti-HPV-07
IFN-α
IFN-γ
TNF-α
TGF-β
IL-1β
IL-2
IL-4
IL-5
IL-6
IL-10
IL-12p40
IL-18
IL-33
CCL2
CCL3
CCL4
CCL5
CCL19
CCL21
CXCL5
CXCL9
CXCL10
FGF-2
FGF-7
VEGF-C
VCAM-1
ICAM-1
MCSF
GMCSF
MIF
MMP-2
MMP-9
Col1A1
ns
***
***
**
ns
**
ns
**
*
*
**
**
ns
ns
**
**
**
*
ns
*
*
*
*
ns
ns
*
*
ns
ns
ns
ns
ns
ns
Log2 Fold Change in Tumors
8
6
4
2
0
−2
B
IFN-γ
TNF-α
TGF-β
IL-2
IL-5
IL-6
IL-10
IL-12p40
IL-18
CCL3
CCL4
CCL5
CCL19
CXCL5
CXCL9
CXCL10
FGF-2
VCAM-1
ICAM-1
Pearson Correlation Coefficient
1.0
0.5
0
−0.5
−1.0

Figure 5.

Figure 5. Tumor inflammatory status in Lenti-HPV-07-vaccinated mice.

A The heatmap represents the $\log_2$ fold change in mRNA expression in the total tumors of mice ($n = 7$/group) engrafted with TC-1 on day 0, vaccinated with Lenti-HPV-07 on day 13, and removed for qRT-PCR analysis of 33 analytes on day 22. For each sample, the mRNA abundance (C_T value) of the target genes was normalized to that of the endogenous β-globin reference gene and compared to a calibrator value corresponding to the mean of the C_T values determined in the tumors from the Ctrl Lenti group. The fold change in gene expression was further calculated using $2^{-\Delta\Delta C_T}$. Statistical significance was evaluated using the Mann–Whitney tests (ns, not significant, *$P < 0.05$, **$P < 0.01$, ***$P < 0.001$).

B Pearson correlation coefficient of differentially expressed analytes in Lenti-HPV-vaccinated mice. Col1A1, collagen, type 1, alpha 1; FGF, fibroblast growth factor; MMP, matrix metalloproteinase.

Source data are available online for this figure.

tumors in 100% of mice, including those bearing large tumors. A single i.m. injection of Lenti-HPV-07 was sufficient to induce $CD8^+$ T cells at the systemic level and to achieve complete eradication of subcutaneously engrafted tumors in all treated mice. Lenti-HPV-07 treatment also showed 100% efficacy in the complete eradication of pulmonary metastatic foci, established through hematogenous tumor spread, and correlated with detectable activated $CD8^+$ T cells and Trm in the lung parenchyma. This observation indicates that i.m. Lenti-HPV-07 therapy also induces a strong antitumor immunity in a mucosal site such as the lungs. Therapeutic vaccination with Lenti-HPV-07 generated a long-lasting memory response that protected the cured mice from tumor recurrence. Mechanistically, this successful therapeutic vaccination elicited polyfunctional tumor immunity, dependent mainly on $CD8^+$ T-cell effectors, leading to deep TME remodeling. Remarkably, antigen-specific peripheral memory $CD8^+$ T cells were still detectable in the spleen of mice beyond 270 days post Lenti-HPV-07 treatment which corresponded to > 195 days after a tumor re-challenge.

We observed an increased influx of hematopoietic cells and mainly $CD8^+$ T cells into the tumor in Lenti-HPV-07-treated tumor-bearing mice when the decline in tumor size began, and the frequency of protection-associated Trm (Masopust & Soerens, 2019) appeared to be rising. Even though Lenti-HPV-07 treatment led to complete tumor eradication, there was an increase in the frequency of intra-tumoral $CD44^{dim}$ $CD69^-$ $KLRG1^+$ exhausted T cells, which most likely resulted from continuous checkpoint signaling and antigenic stimulation. In accordance with this supposition, most intra-tumoral $CD8^+$ T cells also displayed a $PD1^+$ $Tim\text{-}3^+$ phenotype, a proportion of which was also $TCF\text{-}1^+$ (Zhao *et al*, 2022). In addition to its instrumental contribution to early T-cell development, TCF-1 acts in regulating mature T-cell responses, as it is involved in the self-renewal of antitumor $CD8^+$ T cells. TCF-1 contributes to preserving reinforced responses to checkpoint blockade immunotherapy and is considered to be a potential clinical biomarker for the prognosis of tumor immunotherapy (Hong *et al*, 2022; Zhao *et al*, 2022).

PD-L1 overexpression was detected in the regressing tumors, most probably resulting from local inflammatory signals, including IFN-γ release by intra-tumoral $CD8^+$ T cells or NK cells (Cottrell & Taube, 2018). $PD\text{-}L1^+$ tumor cells can interact with $PD1^+$ antigen-specific $CD8^+$ T cells and decrease their effector functions by interfering with the signaling cascade downstream of PD1 (Pauken *et al*, 2021). When the optimal dose of 1×10^9 TU of Lenti-HPV-07 was administered to tumor-bearing mice, this homeostatic mechanism adopted by the tumoral cells was evidently insufficient to counteract the protective ability of Lenti-HPV-07-mediated T-cell immunity. It is possible that T cells induced by Lenti-HPV-07 are mainly of high affinity and thus unaffected or barely affected by the PD-L1-mediated inhibitory signals delivered by tumor cells. Indeed, it has been demonstrated that PD1 signaling preferentially suppresses low-affinity T cells (Liang *et al*, 2017; Shimizu *et al*, 2021). Importantly, we observed a synergistic effect for combined anti-PD1 therapy and a suboptimal dose of Lenti-HPV-07 vaccine, the latter mimicking situations in which T-cell immunity could be insufficient to be fully eradicative, which could result from: (i) large tumor size, (ii) weak tumor accessibility to effector cells, (iii) low E6/E7 antigen presentation, or (iv) partial immune depression and/or T-cell exhaustion. The synergistic effect of the combination of anti-PD1 and Lenti-HPV-07 is also consistent with the reported improvement in tumor eradication potential of LV-based melanoma immunotherapy by PD1/PDL1 blockade (Albershardt *et al*, 2020). In accordance with previous observations, this synergistic beneficial effect was observed only when anti-PD1 therapy was started a few days after Lenti-HPV-07 administration (Curran & Glisson, 2019). This observation is also in agreement with previous results demonstrating a positive role for PD-1 in T-cell effector functions and decreased survival of $CD8^+$ T cells in the absence of PD-1 (Odorizzi *et al*, 2015). Therefore, in a prospective clinical setting, should HPV-induced solid tumors prove refractory or partially resistant to Lenti-HPV-07 therapy, the use of a PD1/PD-L1 blocking immune therapy could be rationally designed to favor T-cell reinvigoration. Of note, HPV16 effective therapeutic vaccine based on synthetic long peptides did not synergize with anti-PD1 treatment in TC1 tumor-bearing mice (van Montfoort *et al*, 2018), highlighting another beneficial aspect of Lenti-HPV-07.

High proportions of NK cells were also found inside the regressing tumors of Lenti-HPV-07-vaccinated mice. NK cells can cytolyze malignant cells that potentially lose their MHC-I expression under the selection pressure of a strong $CD8^+$ T-cell response (Seillet *et al*, 2021) and provide an additional source of intra-tumoral IFN-γ, XCL1, and CCL5 chemo-attractants and Fms-related tyrosine kinase 3 ligand G (FLT3LG), which stimulates DC differentiation/expansion (Demaria *et al*, 2019). In the present study, the depletion of NK cells revealed no role for these cells in regressing the growth of TC-1 cells, which constitutively express MHC-I molecules. However, it cannot be ruled out that in a clinical setting where tumor cells are more prone to lose their MHC-I expression, such NK recruitment could contribute to anti-tumor immunity.

In accordance with TME polarization in Lenti-HPV-07-vaccinated mice, we observed a reduction in the proportion of Tregs, which is usually associated with a good prognosis. It is also noteworthy that an intra-tumoral $CD11b^+$ $CD11c^+$ $MHC\text{-}II^+$ $F4/80^{hi}$ monocyte-derived macrophage/cDC2 subset was detected only in Lenti-HPV-07-vaccinated mice, as also observed in several murine tumoral

© 2023 The Authors

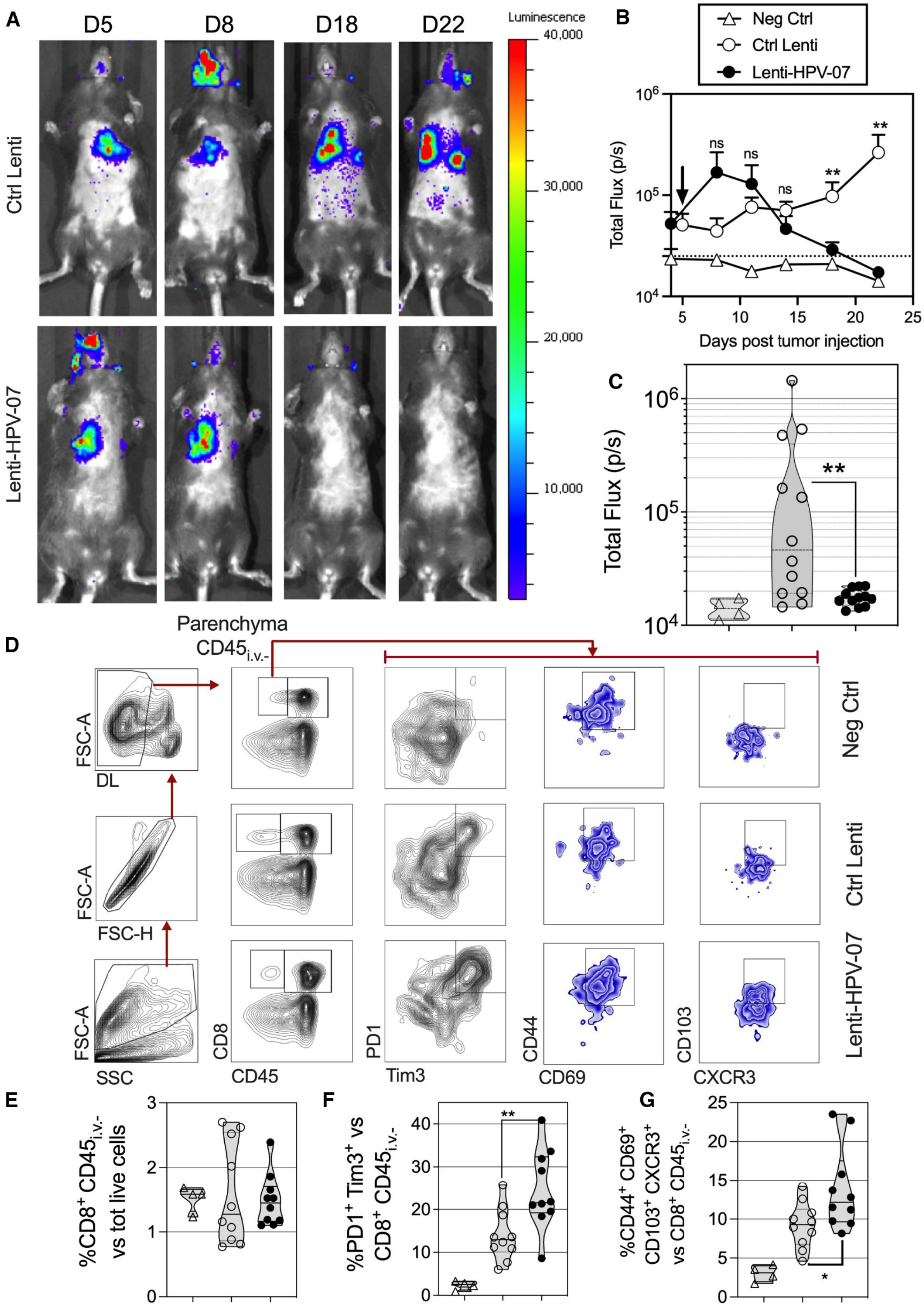
A
D5
D8
D18
D22
Luminescence
40,000
30,000
20,000
10,000
Ctrl Lenti
Lenti-HPV-07
B
Neg Ctrl
Ctrl Lenti
Lenti-HPV-07
Total Flux (p/s)
Days post tumor injection
ns
**
C
Total Flux (p/s)
**
D
Parenchyma
CD45i.v.-
FSC-A
DL
FSC-H
SSC
CD8
CD45
PD1
Tim3
CD44
CD69
CD103
CXCR3
Neg Ctrl
Ctrl Lenti
Lenti-HPV-07
E
%CD8+ CD45i.v.- vs tot live cells
F
%PD1+ Tim3+ vs CD8+ CD45i.v.-
G
%CD44+ CD69+ CD103+ CXCR3+ vs CD8+ CD45i.v.-
*

Figure 6.

Figure 6. Effect of Lenti-HPV-07 immunotherapy on pulmonary metastatic foci.

A C57BL/6 mice were injected i.v. with 1.5×10^5 nLuc-TC-1 cells. On day 5, mice received a single i.m. injection of 1×10^9 TU of Ctrl Lenti or Lenti-HPV-07 ($n = 11$/group). The development of pulmonary metastatic foci was monitored by bioluminescence imaging. The luminescence values of the regions where signals were detectable, that is, thoracic region, were evaluated as the total flux of photons/s (p/s) right after furimazine Z108 substrate administration. The baseline signal was obtained from mice injected with neither nLuc-TC-1 cells nor LVs but injected with the same amounts of furimazine. Representative images obtained from nLuc-TC-1-injected and Ctrl Lenti- or Lenti-HPV-07-treated mice at various time points post-tumor engraftment.

B Follow-up of total flux over time ($n = 11$/group), shown as the mean ± SEM of biological replicates. The black arrow indicates the time point at which Ctrl Lenti or Lenti-HPV-07 were injected. Dotted line represents the basal total flux. Statistical significance was determined using two-tailed unpaired *t*-tests (ns, not significant, $^{**}P < 0.01$).

C The total flux for individual mice of the three experimental groups on day 22 post-tumor engraftment. Statistical significance was determined using two-tailed unpaired *t* tests (ns, not significant, $^{**}P < 0.01$).

D At day 15 post-nLuc-TC-1 i.v. instillation, groups of untreated mice (Neg Ctrl) ($n = 4$) or Lenti Ctrl- ($n = 10$) or Lenti-HPV-07- ($n = 10$) treated and tumor bearing mice received an i.v. injection of PE-anti-CD45 mAb, 3 min before sacrifice. This *in vivo* staining allows cytometrical distinction of lung parenchymal ($CD45_{i.v.-}$) from vascular ($CD45_{i.v.+}$) hematopoietic cells. Live $CD8^+$ $CD45_{i.v.-}$ T cells were gated and analyzed for their expression of various markers. A representative blot for each group is shown.

E–G Percentages of each subset are compared between the groups. Statistical significance was evaluated using the Mann–Whitney tests (ns, not significant, $^{*}P < 0.05$, $^{**}P < 0.01$).

Data information: The experiments shown are representative of at least two independent experiments.
Source data are available online for this figure.

models. The migratory and cross-presentation properties of this population have been well documented (Sheng *et al*, 2017). We also detected higher expression of the pro-inflammatory T-cell cytokines, IL-6, IL-12p40, and IL-18, as well as several chemo-attractants in the TME of the Lenti-HPV-07-vaccinated mice, which was associated with improved outcomes of solid tumors.

Therapeutic vaccination with an adenoviral vaccine expressing E6 and E7 from HPV16 and 18 cured only 10% of animals bearing small TC-1 tumors of 30–60 mm^3 (Hoffmann *et al*, 2010). Another pre-clinical result of TC-1 immunotherapy with an adenoviral vector encoding three HPV antigens, including E6 and E7, reported only a partial reduction in tumor size and a partial increase in the survival rate following a prime-boost regimen (Khan *et al*, 2017). Various studies using *Listeria monocytogenes*-based vaccine vectors for the immunotherapy of HPV-induced cancers (Lm-LLO-E7) have shown widely varying efficacy, with a range of 20–75% of complete cure in mice bearing small TC-1 tumors (Shrimali *et al*, 2017). The detoxified adenylate cyclase of Bordetella pertussis harboring E7 lost its therapeutic effect against large TC-1 tumors and required association with CpG adjuvant and anti-Treg cyclophosphamide treatment to achieve tumor eradication, only occurring in 58% of individuals (Berraondo *et al*, 2007). A DNA vaccine based on the fusion of ubiquitin to HPV E6 and E7 required three injections in a prophylactic setting to avoid TC-1 cell proliferation. In a therapeutic setting, even if only 5.10^4 TC-1 cells were engrafted, three injections of this DNA vaccine led to only partial tumor regression (Chandra *et al*, 2017). In a recent study using mRNA technology, including its auto-amplifying version which prolongs antigen expression in the vaccinated mice, the immunotherapeutic potential of this technology could only be demonstrated against TC-1 tumors, either not yet established or of very small size, with early relapses in almost 50% of treated mice (Ramos da Silva *et al*, 2023). The previously described effective immunotherapies against HPV-induced tumors in preclinical studies (Chu *et al*, 2000; Grasso *et al*, 2013; Domingos-Pereira *et al*, 2019; Arribillaga *et al*, 2020; Ramos da Silva *et al*, 2023) included notably one based on an LV encoding a single HPV antigen (Grasso *et al*, 2013). Compared to this preliminary study and with a view to setting up a clinical trial, Lenti-HPV-07 encodes a poly-antigen targeting the E6 and E7 antigens of both HPV16 and HPV18 genotypes which are most frequently represented among HPV genotypes associated with cancers. Also compared to this previous study, we have thoroughly characterized the phenotype and function of the generated T cells and performed a thorough analysis of the tumor microenvironment which demonstrated a deep remodulation towards an inflammatory state, well beyond a simple activation of specific $CD8^+$ T cells. We have also shown the significant effect of a single i.m. injection of Lenti-HPV-07 against lung metastases, as well as the potential synergy of this therapy with immune checkpoint inhibitors. Therefore, Lenti-HPV-07 therapy outperforms the previously described immune therapy in the standard preclinical HPV-induced cancer models.

One might wonder whether the protein expression of the selected HPV antigens in the host cells transduced by Lenti-HPV-07, Lenti-HPV-08, Lenti-HPV-09, or Lenti-HPV-10 is comparable. It is technically challenging to set up a Western Blot to detect and quantitate the expression level of each of the four HPV antigens in host cells transduced with each of the Lenti-HPVs. In fact, there is no guarantee that the selected antigenic segments in the context of the poly-antigens are recognizable by mAbs which should be specific to sequential—and not conformational—B cell epitopes. However, the comparable T-cell immunogenicity of the four Lenti-HPVs reflects well the comparable antigen availabilities in host cells transduced by each of these Lenti-HPV vectors.

Based on data from the literature, the immunogenicity of $E7_{HPV16}$ in H-2^b mice is most likely responsible for the overall anti-TC-1 tumor response in the C57BL/6 mice (Grasso *et al*, 2013). This antigen alone encoded by viral vectors or other vaccination strategies is the one that achieves the protection against HPV-induced TC-1 tumors. The interest in the inclusion of a poly-antigen in Lenti-HPV-07 was not to broaden the immunogenicity of the vaccine in the C57BL/6 model. The response of C57BL/6 mice to $E7_{HPV16}$ in this model validates the proof of concept. The interest of adding more antigens $E6_{HPV16}$, $E6_{HPV18}$, and $E7_{HPV18}$ antigens was essentially to broaden the antigen/epitope repertoire with a short-term perspective of achieving a clinical trial in patients, with polymorphic HLA, infected by HPV16 or HPV18. It is also noteworthy that patients

© 2023 The Authors

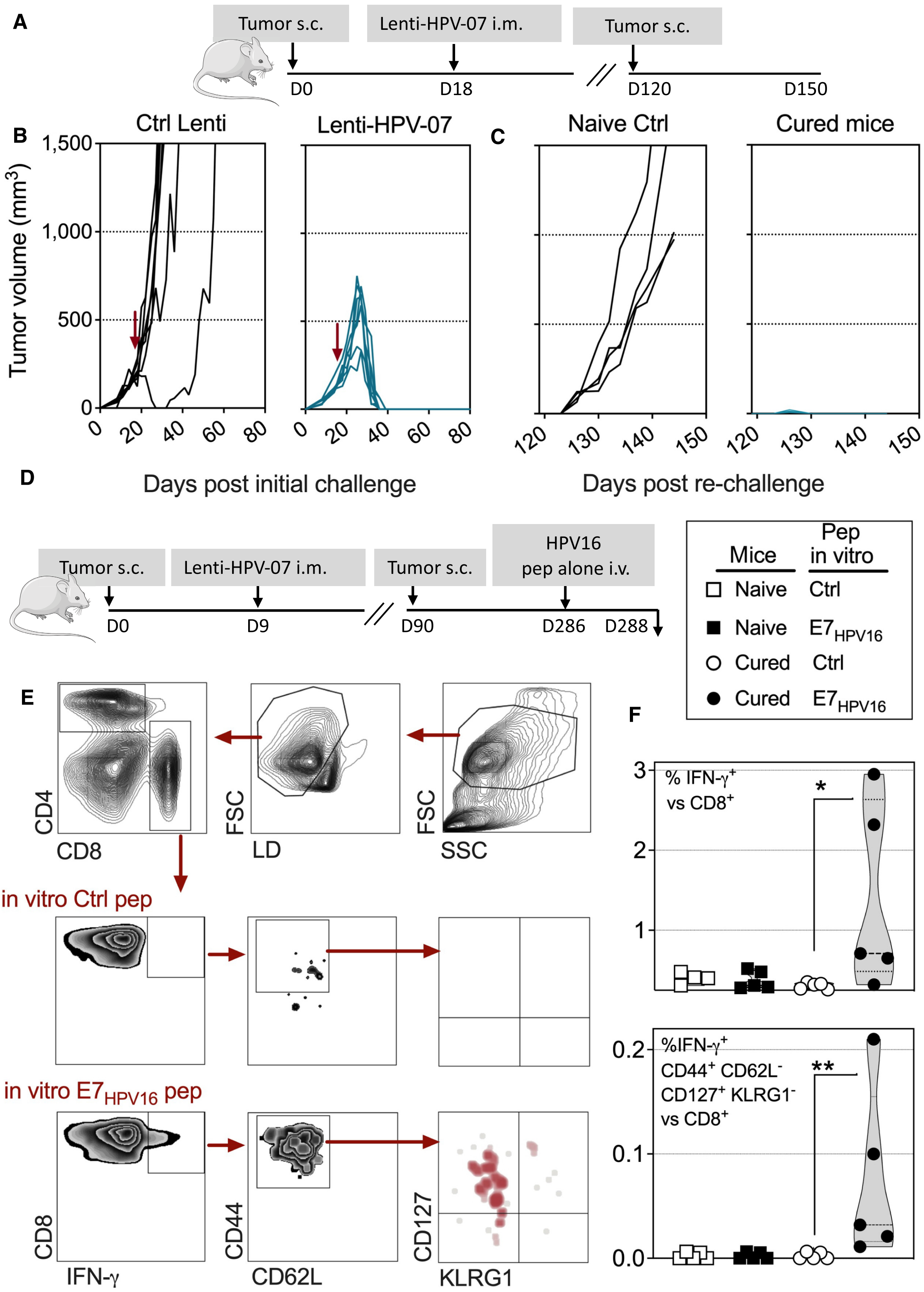
A
Tumor s.c.
Lenti-HPV-07 i.m.
Tumor s.c.
D0
D18
D120
D150
B
Ctrl Lenti
Lenti-HPV-07
Tumor volume (mm3)
1,500
1,000
500
0
0 20 40 60 80
Days post initial challenge
C
Naive Ctrl
Cured mice
120 130 140 150
Days post re-challenge
D
Tumor s.c.
Lenti-HPV-07 i.m.
Tumor s.c.
HPV16
pep alone i.v.
D0
D9
D90
D286
D288
Mice
Pep in vitro
Naive Ctrl
Naive E7HPV16
Cured Ctrl
Cured E7HPV16
E
CD4
CD8
FSC
LD
FSC
SSC
in vitro Ctrl pep
in vitro E7HPV16 pep
CD8
IFN-γ
CD44
CD62L
CD127
KLRG1
F
% IFN-γ+ vs CD8+
*
3
2
1
%IFN-γ+ CD44+ CD62L- CD127+ KLRG1- vs CD8+
**
0.2
0.1
0.0

Figure 7.

Figure 7. Long-lasting antitumor T-cell memory induced by a single Lenti-HPV-07 vaccination.

A Timeline of s.c. tumor engraftment and Lenti-HPV-07 vaccination. C57BL/6 mice ($n = 7$) were engrafted on the flank with 1×10^6 tumor cells. On day 18 post-engraftment, when the tumor volume reached an average of 200 mm^3, mice were randomized. Lenti-HPV-07 vaccination was performed on day 18. Tumor-bearing negative control animals received Ctrl Lenti.

B Spaghetti plots of tumor growth. The red arrows indicate the time point at which Ctrl Lenti or Lenti-HPV-07 were injected.

C On day 120, cured mice were engrafted s.c. with 1×10^6 tumor cells on the opposite flank and left untreated to mimic tumor relapse. Age-matched naïve control mice ($n = 4$) showed the growth capacity of the engrafted tumor cells used in the relapse part of the experiment.

D–F In an independent experiment, Lenti-HPV-treated and cured mice which have been re-challenged ($n = 5$/group), received at day 286 post tumor engraftment an i.v. injection of an adjuvant-free mixture of $E7_{HPV16}$:ETTDPDRAHYNIVTF and $E7_{HPV16}$:PDRAHYNIVTFCCKC peptides (25 µg of each/mouse). Naïve control mice received the same i.v. treatment. At 48 h post injection, splenocytes were stimulated *in vitro* with homologous or negative control peptides during 6 h prior to IFN-γ ICS and surface labeling. (E) Gating strategy and representative blots. (F) Percentages of IFN-γ^+ versus total $CD8^+$ T cells (top) and those of IFN-γ^+ $CD44^+$ $CD62L^-$ $CD127^+$ $KLRG1^-$ (memory precursor cells) (bottom) versus total $CD8^+$ T cells. Statistical significance was determined by the Mann–Whitney *t*-test (*$P < 0.05$, **$P < 0.01$).

Data information: The experiments shown are representative of at least two independent experiments.
Source data are available online for this figure.

with HPV16 and HPV18 co-infection have been reported (Jesus *et al*, 2018).

Most murine HPV tumor models rely on s.c. engraftment. Hence, physiological HPV infections occur in the internal tissues and mucosal epithelia, that is, vaginal, anal, or oropharyngeal mucosa. Mucosal sites have specific environments, their cell composition is distinct from that in lymphoid organs and peripheral blood, and they may be poorly accessible to T cells induced following systemic immunization. It is possible that local immunotherapy of HPV-induced cancers would be more effective and more likely to trigger Trm. Due to their high safety because of their non-replicative, non-cytopathic, and non-inflammatory properties, LVs are suitable for mucosal vaccination and can induce local effector and Trm immunity at mucosal sites. We have successfully used LV in pre-clinical settings for the complete and total prevention of the respiratory and central nervous systems from SARS–CoV-2 infection (Ku *et al*, 2021a,c,d) and for the prevention of the respiratory system from *Mycobacterium tuberculosis* infection (Lopez *et al*, 2022). The benefit of mucosal antitumor $CD8^+$ T-cell induction by intranasal vaccination at the anatomical sites of HPV-induced malignancy has been demonstrated in orthotopic head and neck murine cancer (Sandoval *et al*, 2013). Beneficial effects of the induction of HPV-specific T-cell immunity in the cervico-vaginal mucosa following intravaginal vaccination have also been reported in mice (Echchannaoui *et al*, 2008; Zottnick *et al*, 2020). When translating the Lenti-HPV-07 immuno-oncotherapy into the clinic, more than one injection of Lenti-HPV-07 may be necessary to reach optimal vaccine efficacy depending on the patient's immunological and chemo-/radio-therapeutic history. Booster injections via the nasal, vaginal, and rectal routes could then be a feasible and mechanistically justifiable alternative. It is noteworthy that the lack of HPV antigen positioning effect on immunogenicity, observed with different Lenti-HPV encoding various antigen permutations, suggests that there will be no impact on the functional T-cell immune response in a human population where various epitopes will need to be presented by HLA molecules of multiple alleles.

Limitations of the study

The transplantable TC-1 model is the standard to evaluate the efficacy of HPV vaccine candidates (Zottnick *et al*, 2020), is used by almost all expert laboratories, and allows the comparison of results generated in different studies. There are other more aggressive models, such as low MHC-I expressing TC-1 P3 (A15) cells (Cheng *et al*, 2003), not tested in the present study. However, we showed the high efficacy of a single injection of Lenti-HPV-07 in the alternative aggressive large TC-1 tumor model, that is, mice bearing tumors with an average volume of 450 mm^3. TC-1 cells may express higher levels of the E6 and E7 antigens than HPV-induced human tumors. Therefore, we cannot exclude that the complete control of TC-1 by Lenti-HPV-07 therapy may only partially recapitulate the characteristics of HPV-induced human tumors. Several therapeutic HPV vaccine candidates have reached clinical trials based on the proof of concept established in this model (Smalley Rumfield *et al*, 2020). Consequently, these preclinical results may have partial predictive value for efficacy in human HPV-induced cancers. However, given the encouraging results obtained with Lenti-HPV, the present results prompt an evaluation of the efficacy of treatment with Lenti-HPV-07 in patients.

Figure 8. Anti-PD1 treatment synergizes with suboptimal dose of Lenti-HPV-07.

A Timeline of tumor s.c. engraftment and combinatory Lenti-HPV-07 and anti-PD1 mAb treatment. C57BL/6 mice ($n = 10$–14/group) were engrafted on the flank with 1×10^6 tumor cells. On day 13 post-engraftment, when the tumor volume reached an average of 120–140 mm^3, mice were treated with the suboptimal dose of 1×10^8 TU of Ctrl Lenti or Lenti-HPV-07. Mice were then treated 2–3 times a week with anti-PD1 mAb for a total of six injections from days 17 to 31.

B Spaghetti plots of tumor growth. The gray arrows indicate the time point at which Ctrl Lenti or Lenti-HPV-07 were injected.

C Percentages of mice, without, with partial or complete antitumor response.

D Progression-free survival (PFS) time of mice bearing TC-1 tumors treated with suboptimal dose of Lenti-HPV07 and anti-PD-1. PFS described the duration of response from the day that tumor reaches maximum size until the day that tumor exceeds maximum size again in responsive mice ($n = 10$–14/group). Statistical significance was determined using two-tailed unpaired *t* tests (*$P < 0.05$).

E Survival curves of animals ($n = 10$–14/group), followed for 85 days. Statistical significance was determined using Log-rank Mantel-Cox tests, (*$P = 0.0159$, **$P = 0.071$). Mice were sacrificed when the size of the tumors reached 1,500 mm^3, in accordance with the defined humane endpoints.

Data information: The experiments shown are representative of at least two independent experiments.
Source data are available online for this figure.

 © 2023 The Authors

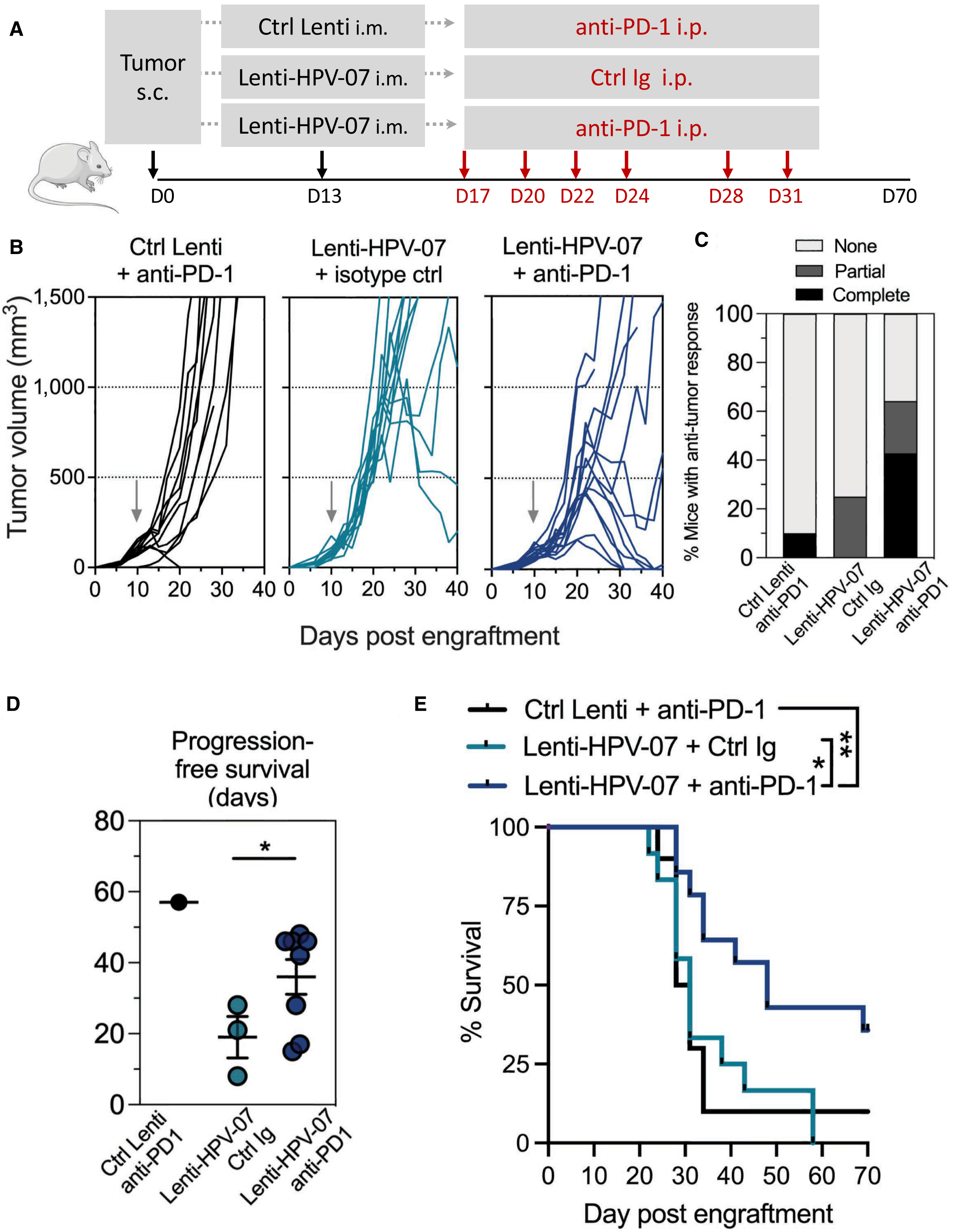
A
Tumor s.c.
Ctrl Lenti i.m.
Lenti-HPV-07 i.m.
Lenti-HPV-07 i.m.
anti-PD-1 i.p.
Ctrl Ig i.p.
anti-PD-1 i.p.
D0
D13
D17
D20
D22
D24
D28
D31
D70
B
Ctrl Lenti + anti-PD-1
Lenti-HPV-07 + isotype ctrl
Lenti-HPV-07 + anti-PD-1
Tumor volume (mm3)
Days post engraftment
C
None
Partial
Complete
% Mice with anti-tumor response
Ctrl Lenti anti-PD1
Lenti-HPV-07 Ctrl Ig
Lenti-HPV-07 anti-PD1
D
Progression-free survival (days)
Ctrl Lenti anti-PD1
Lenti-HPV-07 Ctrl Ig
Lenti-HPV-07 anti-PD1
E
Ctrl Lenti + anti-PD-1
Lenti-HPV-07 + Ctrl Ig
Lenti-HPV-07 + anti-PD-1
% Survival
Day post engraftment

Figure 8.

A phase 1/IIa clinical trial is presently in preparation to evaluate the efficacy of Lenti-HPV-07 in HPV16/18^{+} cervical and head and neck cancers.

Materials and Methods

Construction and production of Lenti-HPV

Codon-optimized sequences of the designed E6-E7 polyantigens were synthesized and inserted into the pFlap lentiviral plasmid between the BamHI and XhoI sites, located between the human 2-microglobulin promoter and the mutated *atg* starting codon of the woodchuck post-transcriptional regulatory element (WPRE) sequence (Appendix Fig S4). The envelope plasmid encodes VSV-G under the cytomegalovirus promoter, and the packaging plasmid contains *gag*, *pol*, *tat*, and *rev* genes. The integrase resulting from the packaging plasmid carries a missense amino acid in its catalytic triad, that is, the D64V mutation, which prevents the integration of viral DNA into the host chromosome (Ku *et al*, 2021d).

Mice

Six- to 12-week-old female C57BL/6JRj mice (Janvier, Le Genest Saint Isle, France) were housed in ventilated cages under specific pathogen-free conditions at the Institut Pasteur animal facilities. All the procedures were performed in accordance with the European and French guidelines (Directive 86/609/CEE and Decree 87–848 of October 19, 1987) after approval of the protocol by the Institut Pasteur Safety, Animal Care and Use Committee delivered by the local ethics committee (CETEA #DAP180049, CETEA #DAP190130 and Ministry of High Education and Research APAFIS#16381-2018080217194542 v1, APAFIS# 20981-20190606164112731.

Tumor and treatments

TC-1 cells (ATCC, CRL-2785) are C57BL/6 lung cells immortalized by stable co-transfection of HPV16 E6 and E7 and HRas carrying the G12V activating mutation, which makes them tumorigenic. TC-1 cells were authenticated by qRT–PCR specific to genes encoding HPV16 E6 and E7 and tested negative for mycoplasma contamination by use of MycoAler Plus Kit (Lonza). TC-1 cells were cultured in RPMI 1640 supplemented with 10% FBS, penicillin (100 U/ml), and streptomycin (100 μg/ml) at 37°C in 5% CO_2 and used at a low passage number. Mice were engrafted s.c. with 1×10^6 TC-1 cells on the right flank at day 0. Nine to 18 days later, when the tumor size reached 80–120 or 200 mm^3, mice were randomized and injected i.m. with a single injection of Lenti-HPV contained in 50 μl. Anti-PD1 (clone RMP1-14, Bioxcell) or control Ig (clone 2A3, Bioxcell) was injected i.p. at 200 μg/mouse, 2 or 3 times a week, for a total of six injections. Anti-CD4 (clone GK1.5, in the house), anti-CD8 (clone H35.17.2, in the house), or control Ig was injected i.p. at 250 μg/mouse the day before the vaccination and then every other day until day 15 post-vaccination. Anti-NK1.1 (clone PK136, Bioxcell), or control Ig, was injected i.p. at 300 μg/mouse the day before the vaccination and then every other day until day 15 post-vaccination. Tumors were measured 2 or 3 times a week using a digital caliper and the tumor volume was calculated using the formula $V = L \times W^2/2$, where V is the volume, L the length (the longest diameter), and W the width (the shortest diameter). Mice were sacrificed when tumors reached 1,500 mm^3 in volume or became ulcerated or when the mice became moribund.

T-cell assays

As recently detailed elsewhere (Ku *et al*, 2021c) T splenocyte responses were assessed by IFN-γ ELISPOT after *in vitro* stimulation with E6 or E7 peptide pools or individual peptides. ICS assay was performed on splenocytes as recently detailed (Lopez *et al*, 2022).

Cytometric analysis of tumor immune infiltrates

Tumors were treated with the Mouse Tumor Dissociation kit (Miltenyi). Cell suspensions were then filtered through 70 μm-pore filters, treated with Red Blood Cell lysis buffer (Sigma), and then washed and centrifuged at 1,200 rpm for 5 min. The recovered cells were stained as follows. (i) To detect DC and macrophages, Near IR Live/Dead (Invitrogen), FcγII/III receptor blocking anti-CD16/CD32 (clone 2.4G2, BD Biosciences), BV605-anti-CD45 (clone 30-F11, BD Biosciences), PE-anti-CD11b (clone M1/70, eBioscience), PE-Cy7-anti-CD11c (clone N418, eBioscience), PerCP-Cy5.5-anti-MHC-II (clone M5/114, BD Biosciences), FITC-anti-CD24 (clone M1/69, BD Biosciences), and BV421-anti-F4/80 (clone T45-2342, BD Biosciences) were used. (ii) To detect B cells, NK cells, and PMNs, Near IR LD (Invitrogen), FcγII/III receptor blocking anti-CD16/CD32 (clone 2.4G2, BD Biosciences), BV605-anti-CD45 (clone 30-F11, BD Biosciences), FITC-anti-B220 (clone RA3-6B2, eBioscience) APC-anti-CD11b (clone N418, BD Biosciences), PE-Cy7-anti-CD11c (clone N418, eBioscience), PE-anti-Ly6G (clone 1A8, Biolegend), BV421-anti-NKp46 (clone 29A1.4, Biolegend), and PerCP-Cy5.5-anti-Ly6C (clone HK1.4, eBioscience) were used. (iii) To detect T cells and their activation profile, Near IR Live/Dead (Invitrogen), FcγII/III receptor blocking anti-CD16/CD32 (clone 2.4G2, BD Biosciences), BV605-anti-CD45 (clone 30-F11, BD Biosciences), APC-anti-CD8 (clone 53-6.7, eBioscience), eF450-anti-CD4 (clone RM4-5, eBioscience), BV711-anti-CD103 (clone 2E7, Biolegend), PE-Cy7-anti-CD69 (clone H1.2F3, BD Biosciences), FITC-anti-CD44 (clone IM7, Biolegend), and PerCP-eF710-anti-KLRG1 (clone 2F1, eBioscience) were used. Samples were incubated with appropriate mAb mixtures for 25 min at 4°C, washed with PBS + 3% FBS (FACS buffer), and fixed with Cytofix (BD Biosciences) for 20 min at 4°C. (iv) To identify T-cell subsets and PD-L1 expression in tumors, Near IR Live/Dead (Invitrogen), FcγII/III receptor blocking anti-CD16/CD32 (clone 2.4G2, BD Biosciences), BV605-anti-CD45 (clone 30-F11, BD Biosciences), BV711-anti-CD8 (clone 53-6.7, BD Biosciences), PerCP-Cy5.5-anti-CD4 (clone RM4-5, eBioscience), FITC-anti-PD1 (clone J43, eBioscience), PE-Cy7-anti-Tim-3 (clone RMT3-23, eBioscience), and APC-anti-PD-L1 (clone 10F.9G2 Biolegend) were used. Cells were incubated with appropriate mixtures for 25 min at 4°C and washed with FACS buffer. Cells were then washed in FACS buffer and permeabilized using the Cytofix/Cytoperm kit (BD Bioscience). Cells were again washed twice with 1X PermWash (Cytofix/Cytoperm kit) and incubated with AF405-anti-TCF1 (clone 812545, R&D systems) or control Ig for 30 min at 4°C. Cells were then washed once in 1× PermWash and once in FACS buffer and fixed with Cytofix (BD Biosciences) for 20 min at 4°C.

© 2023 The Authors

Intra-tumoral cells were assessed for Treg using the Treg Detection Kit (Miltenyi). Sample data were acquired in an Attune NxT cytometer (Invitrogen) and the data was analyzed using FlowJo software (Treestar, OR, USA).

Immunohistochemistry

Tumor samples, fixed for 48 h in 10% neutral-buffered formalin, were embedded in the Optimal Cutting Temperature compound (Tissue-Tek Sakura Cat# 4583). Immunohistochemistry was performed on 7-μm-thick cryostat sections on Leica Bond RX using rabbit anti-mouse CD4 (Cell signaling, D7D2Z, #25229) or CD8 (Cell signaling, D8A8Y, #85336) antibody and biotinylated goat anti-rabbit Ig secondary antibody (E0432, Dako, Agilent, France). Slides were then scanned using an Axioscan Z1 Zeiss slide scanner and the images were analyzed using Zen 2.6 software.

qRT–PCR

The qRT–PCR quantification of inflammatory mediators or other cancer-related analytes in the tumors from Ctrl Lenti- or Lenti-HPV-07-vaccinated mice was performed using the primer pairs listed in Appendix Table S1 and as recently detailed (Ku *et al*, 2021c) using the comparative $\Delta\Delta C_T$ method. Total RNA was extracted using the RNeasy kit (Qiagen). Genomic DNA was eliminated using RNase-Free DNAse (Qiagen) and the samples were immediately stored at −80°C. The RNA quality was checked using a Bioanalyzer 2100 (Agilent Technologies). RNA samples were quantitated using a NanoDrop (Thermo Scientific NanoDrop). The RNA Integrity Number (RIN) was 8.0–10.0. For each sample, the mRNA abundance (C_T value) of the target genes was normalized to the endogenous β-globin reference gene and compared to a calibrator value, corresponding here to the mean of C_T values determined in the tumors from the Ctrl Lenti-vaccinated mice. The fold change in gene expression was further calculated using $2^{-\Delta\Delta C_T}$.

In vivo animal imaging

The TC-1 parental cell line was stably transduced with an integrative LV encoding the *Oplophorus gracilirostris*-derived nanoluciferase reporter and puromycin N-acetyl-transferase (for selection) under the UBC ubiquitin promoter. After selection on puromycin, cells were subcloned to obtain the "nLuc-TC-1" cell line. Six-week-old C57BL/6JRj mice, purchased from Janvier Laboratory, were injected i.v. with 1.5×10^5 nLuc-TC-1 cells. On day 5, mice were injected i.m. with a single dose of 1×10^9 TU Lenti-HPV-07/mouse or a Ctrl Lenti. Bioluminescence imaging on live animals was performed using the IVIS Imaging System (IVIS Spectrum, Perkin Elmer) coupled to a charged-couple device camera. Prior to bioluminescence imaging, mice were anesthetized with 2% isoflurane in oxygen and maintained in control flow of 1.5% isoflurane in oxygen through a nose cone during imaging. The Z108 substrate furimazine was dissolved at 2 mg/ml in acidic ethanol. Furimazine was further diluted in sterile PBS to the desired concentration prior to injection. Mice bellies and torsos were shaved to enhance the signal-to-noise ratio. Furimazine was injected i.v. at 0.4 mg/kg. Mice were then immediately placed in the imaging chamber and imaged. Sequential images were captured under the auto-exposure settings with a maximum exposure time of 2 min. Images from each experimental set were analyzed using Living Image Software (Ver. 2.50.1 Xenogen). Measurements from regions of interest were selected and the luminescence values evaluated as the total flux (photon/s [p/s]). The baseline signals were obtained from untreated mice, that is, injected with neither nLuc-TC-1 cells nor lentiviral-based vaccines.

Statistical analyses

Statistical significance was determined using the two-tailed unpaired *t*-test. When indicated, the data were subjected to PCA and Pearson correlation coefficient analyses. The statistical significance of the percent survival was determined using the Log-rank Mantel-Cox test. All the statistical analyses were performed using Prism GraphPad v9. Mice were randomized on the day of immunization. Mice were allocated to cages so that each cage contained individuals representing each treatment.

The paper explained

Problem
Human papillomavirus causes almost all cervical and numerous oropharyngeal and anogenital cancers. The currently available HPV vaccines mainly trigger anti-HPV neutralizing antibodies and have no effect against HPV-induced tumors. By use of the non-integrative LV vaccinal platform, we developed an anti-HPV immuno-oncotherapy, that is, Lenti-HPV-07. The latter encodes Early E6 and E7 immunogens of HPV16 and 18 genotypes.

Results
In a preclinical animal model, a single intramuscular administration of Lenti-HPV-07 into mice bearing small or large HPV-induced tumors induces robust and long-term memory and effector $CD8^+$ T-cell, profound remodeling of the tumor microenvironment to an inflammatory status, full tumor eradication and full clearance of metastases in 100% of the animals. A single Lenti-HPV-07 administration prevented HPV-induced tumor relapse.

Impact
Lentiviral vectors are efficient at inducing immunity, are non-inflammatory, and have an excellent safety record as demonstrated in a previous HIV therapeutic clinical trial. Lenti-HPV-07 immuno-oncotherapy is efficient in the preclinical model and can synergize with other immunotherapies like anti-checkpoint inhibitory treatments. Lenti-HPV-07 therapy emerges as a promising immuno-oncotherapy against established HPV-mediated tumors. A phase I/II clinical trial is currently in preparation.

Data availability

The published article includes all datasets generated and analyzed during this study. All plasmids and LV generated in this study will be available under an MTA for research use, given a pending patent directed to Lenti-HPV vaccination vectors. Further information and requests for resources and reagents should be directed to and will be fulfilled by the corresponding author Laleh Majlessi (laleh.majlessi@pasteur.fr). Cytometric raw data are available at:

© 2023 The Authors

Figure 2B and D. AccessionS-BSST1100: https://www.ebi.ac.uk/biostudies/studies/S-BSST1100?key = ffd75bef-d367-43a9-9cd7-7d938ee5e0bc.
Figure 3A, B and D–F. AccessionS-BSST1101: https://www.ebi.ac.uk/biostudies/studies/S-BSST1101?key = fb475118-ba96-45cd-b06d-3aaed8e3d81d.
Figure 3G and H. AccessionS-BSST1102: https://www.ebi.ac.uk/biostudies/studies/S-BSST1102?key = a849638e-4e87-4034-bc3a-50bafab93967.
Figure 4A and B. AccessionS-BSST1103: https://www.ebi.ac.uk/biostudies/studies/S-BSST1103?key = 3998e228-90e0-4b1d-b327-08cd95251b44.
Figure 4C. AccessionS-BSST1104: https://www.ebi.ac.uk/biostudies/studies/S-BSST1104?key = fbceaa34-7251-402e-a6aa-aeacb7492d99.
Figure 6D. AccessionS-BSST1107: https://www.ebi.ac.uk/biostudies/studies/S-BSST1107?key = d4f25081-5a09-4fc7-94c7-d35858298c35.
Figure 7. AccessionS-BSST1108: https://www.ebi.ac.uk/biostudies/studies/S-BSST1108?key = cbe661ed-b971-474b-b73d-97714cf2e94a.

Expanded View for this article is available online.

Acknowledgments

This work was supported by TheraVectys and Institut Pasteur. The authors are grateful to Dr. Yves Janin (Institut Pasteur) for the gift of the substrate furimazine (Z108) and Johan Bedel for excellent technical assistance in preparing histological sections.

Author contributions

Laëtitia Douguet: Conceptualization; data curation; formal analysis; methodology. **Ingrid Fert:** Conceptualization; data curation; formal analysis; methodology. **Jodie Lopez:** Data curation; formal analysis; methodology. **Benjamin Vesin:** Data curation; formal analysis; methodology. **Fabien Le Chevalier:** Data curation; formal analysis; methodology. **Fanny Moncoq:** Data curation; formal analysis; methodology. **Pierre Authié:** Data curation; formal analysis; methodology. **Trang-My Nguyen:** Data curation. **Amandine Noirat:** Data curation; formal analysis; methodology. **Fabien Névo:** Data curation; formal analysis; methodology. **Catherine Blanc:** Data curation; formal analysis; methodology. **Maryline Bourgine:** Methodology. **David Hardy:** Data curation; formal analysis; methodology. **François Anna:** Conceptualization. **Laleh Majlessi:** Conceptualization; formal analysis; supervision; validation; investigation; writing – original draft; project administration; writing – review and editing. **Pierre Charneau:** Conceptualization; resources; supervision; funding acquisition; validation; investigation; project administration.

Disclosure and competing interests statement

LD, IF, BV, FLC, FM, PA, T-MN, AN, FN, FA, LM, and PC declare competing financial interests related to the publication of this study. PC is the founder and CSO of TheraVectys. LD, IF, BV, FLC, FM, PA, T-MN, AN, FN, and FA are employees of TheraVectys. LM has a consultancy activity for TheraVectys. LD, IF, FM, AN, FA, LM, and PC are inventors of a pending patent directed to the potential of Lenti-HPV-07 vaccination against HPV-induced cancers. Other authors declare no competing interests.

For more information

https://theravectys.site/.

References

Adotevi O, Mollier K, Neuveut C, Dosset M, Ravel P, Fridman WH, Tartour E, Charneau P, Wain-Hobson S, Langlade-Demoyen P (2010) Targeting human telomerase reverse transcriptase with recombinant lentivector is highly effective to stimulate antitumor CD8 T-cell immunity *in vivo*. *Blood* 115: 3025–3032

Albershardt TC, Parsons AJ, Reeves RS, Flynn PA, Campbell DJ, Ter Meulen J, Berglund P (2020) Therapeutic efficacy of PD1/PDL1 blockade in B16 melanoma is greatly enhanced by immunization with dendritic cell-targeting lentiviral vector and protein vaccine. *Vaccine* 38: 3369–3377

Allen F, Bobanga ID, Rauhe P, Barkauskas D, Teich N, Tong C, Myers J, Huang AY (2018) CCL3 augments tumor rejection and enhances CD8(+) T cell infiltration through NK and CD103(+) dendritic cell recruitment via IFNgamma. *Onco Targets Ther* 7: e1393598

Altmann DM, Douek DC, Frater AJ, Hetherington CM, Inoko H, Elliott JI (1995) The T cell response of HLA-DR transgenic mice to human myelin basic protein and other antigens in the presence and absence of human CD4. *J Exp Med* 181: 867–875

Anderson KG, Mayer-Barber K, Sung H, Beura L, James BR, Taylor JJ, Qunaj L, Griffith TS, Vezys V, Barber DL *et al* (2014) Intravascular staining for discrimination of vascular and tissue leukocytes. *Nat Protoc* 9: 209–222

Anna F, Lopez J, Moncoq F, Blanc C, Authie P, Noirat A, Fert I, Souque P, Nevo F, Pawlik A *et al* (2022) A lentiviral vector expressing a dendritic cell-targeting multimer induces mucosal anti-mycobacterial CD4(+) T-cell immunity. *Mucosal Immunol* 15: 1389–1404

Arribillaga L, Echeverria I, Belsue V, Gomez T, Lozano T, Casares N, Villanueva L, Domingos-Pereira S, Romero PJ, Nardelli-Haefliger D *et al* (2020) Bivalent therapeutic vaccine against HPV16/18 genotypes consisting of a fusion protein between the extra domain A from human fibronectin and HPV16/18 E7 viral antigens. *J Immunother Cancer* 8: e000704

Bellacchio E, Paggi MG (2013) Understanding the targeting of the RB family proteins by viral oncoproteins to defeat their oncogenic machinery. *J Cell Physiol* 228: 285–291

Berraondo P, Nouze C, Preville X, Ladant D, Leclerc C (2007) Eradication of large tumors in mice by a tritherapy targeting the innate, adaptive, and regulatory components of the immune system. *Cancer Res* 67: 8847–8855

van Boerdonk RA, Daniels JM, Bloemena E, Krijgsman O, Steenbergen RD, Brakenhoff RH, Grunberg K, Ylstra B, Meijer CJ, Smit EF *et al* (2013) High-risk human papillomavirus-positive lung cancer: molecular evidence for a pattern of pulmonary metastasis. *J Thorac Oncol* 8: 711–718

Chandra J, Dutton JL, Li B, Woo WP, Xu Y, Tolley LK, Yong M, Wells JW, R Leggatt G, Finlayson N *et al* (2017) DNA vaccine encoding HPV16 oncogenes E6 and E7 induces potent cell-mediated and humoral immunity which protects in tumor challenge and drives E7-expressing skin graft rejection. *J Immunother* 40: 62–70

Cheng WF, Hung CF, Lin KY, Ling M, Juang J, He L, Lin CT, Wu TC (2003) CD8+ T cells, NK cells and IFN-gamma are important for control of tumor with downregulated MHC class I expression by DNA vaccination. *Gene Ther* 10: 1311–1320

Chu NR, Wu HB, Wu TC, Boux LJ, Mizzen LA, Siegel MI (2000) Immunotherapy of a human papillomavirus type 16 E7-expressing tumor by administration of fusion protein comprised of *Mycobacterium bovis* BCG Hsp65 and HPV16 E7. *Cell Stress Chaperones* 5: 401–405

Cottrell TR, Taube JM (2018) PD-L1 and emerging biomarkers in immune checkpoint blockade therapy. *Cancer J* 24: 41–46

Curran MA, Glisson BS (2019) New hope for therapeutic cancer vaccines in the era of immune checkpoint modulation. *Annu Rev Med* 70: 409–424

 © 2023 The Authors

Demaria O, Cornen S, Daeron M, Morel Y, Medzhitov R, Vivier E (2019) Harnessing innate immunity in cancer therapy. *Nature* 574: 45–56

Domingos-Pereira S, Galliverti G, Hanahan D, Nardelli-Haefliger D (2019) Carboplatin/paclitaxel, E7-vaccination and intravaginal CpG as tri-therapy towards efficient regression of genital HPV16 tumors. *J Immunother Cancer* 7: 122

Doorbar J, Quint W, Banks L, Bravo IG, Stoler M, Broker TR, Stanley MA (2012) The biology and life-cycle of human papillomaviruses. *Vaccine* 30: F55–F70

Douguet L, Bod L, Labarthe L, Lengagne R, Kato M, Couillin I, Prevost-Blondel A (2018) Inflammation drives nitric oxide synthase 2 expression by gammadelta T cells and affects the balance between melanoma and vitiligo associated melanoma. *Onco Targets Ther* 7: e1484979

Eberhardt CS, Kissick HT, Patel MR, Cardenas MA, Prokhnevska N, Obeng RC, Nasti TH, Griffith CC, Im SJ, Wang X *et al* (2021) Functional HPV-specific PD-1(+) stem-like CD8 T cells in head and neck cancer. *Nature* 597: 279–284

Echchannaoui H, Bianchi M, Baud D, Bobst M, Stehle JC, Nardelli-Haefliger D (2008) Intravaginal immunization of mice with recombinant *Salmonella enterica* serovar Typhimurium expressing human papillomavirus type 16 antigens as a potential route of vaccination against cervical cancer. *Infect Immun* 76: 1940–1951

Feltkamp MC, Smits HL, Vierboom MP, Minnaar RP, de Jongh BM, Drijfhout JW, ter Schegget J, Melief CJ, Kast WM (1993) Vaccination with cytotoxic T lymphocyte epitope-containing peptide protects against a tumor induced by human papillomavirus type 16-transformed cells. *Eur J Immunol* 23: 2242–2249

Garcia-Nicolas O, Ricklin ME, Liniger M, Vielle NJ, Python S, Souque P, Charneau P, Summerfield A (2017) A Japanese encephalitis virus vaccine inducing antibodies strongly enhancing *in vitro* infection is protective in pigs. *Viruses* 9: 124

Grasso F, Negri DR, Mochi S, Rossi A, Cesolini A, Giovannelli A, Chiantore MV, Leone P, Giorgi C, Cara A (2013) Successful therapeutic vaccination with integrase defective lentiviral vector expressing nononcogenic human papillomavirus E7 protein. *Int J Cancer* 132: 335–344

Hampson IN (2022) Effects of the prophylactic HPV vaccines on HPV type prevalence and cervical pathology. *Viruses* 14: 757

Hiam-Galvez KJ, Allen BM, Spitzer MH (2021) Systemic immunity in cancer. *Nat Rev Cancer* 21: 345–359

Hoffmann C, Stanke J, Kaufmann AM, Loddenkemper C, Schneider A, Cichon G (2010) Combining T-cell vaccination and application of agonistic anti-GITR mAb (DTA-1) induces complete eradication of HPV oncogene expressing tumors in mice. *J Immunother* 33: 136–145

Hong W, Yang B, He Q, Wang J, Weng Q (2022) New insights of CCR7 signaling in dendritic cell migration and inflammatory diseases. *Front Pharmacol* 13: 841687

Hussain SS, Lundine D, Leeman JE, Higginson DS (2021) Genomic signatures in HPV-associated tumors. *Viruses* 13: 1998

Iglesias MC, Frenkiel MP, Mollier K, Souque P, Despres P, Charneau P (2006) A single immunization with a minute dose of a lentiviral vector-based vaccine is highly effective at eliciting protective humoral immunity against West Nile virus. *J Gene Med* 8: 265–274

Jesus SP, Costa A, Barcellos RB, Medeiros RM, Silva C, Rossetti ML (2018) A high prevalence of human papillomavirus 16 and 18 co-infections in cervical biopsies from southern Brazil. *Braz J Microbiol* 49: 220–223

Ji H, Chang EY, Lin KY, Kurman RJ, Pardoll DM, Wu TC (1998) Antigen-specific immunotherapy for murine lung metastatic tumors expressing human papillomavirus type 16 E7 oncoprotein. *Int J Cancer* 78: 41–45

Khan S, Oosterhuis K, Wunderlich K, Bunnik EM, Bhaggoe M, Boedhoe S, Karia S, Steenbergen RDM, Bosch L, Serroyen J *et al* (2017) Development of a replication-deficient adenoviral vector-based vaccine candidate for the interception of HPV16- and HPV18-induced infections and disease. *Int J Cancer* 141: 393–404

Kobayashi K, Hisamatsu K, Suzui N, Hara A, Tomita H, Miyazaki T (2018) A review of HPV-related head and neck cancer. *J Clin Med* 7: 241

Ku MW, Anna F, Souque P, Petres S, Prot M, Simon-Loriere E, Charneau P, Bourgine M (2020) A single dose of NILV-based vaccine provides rapid and durable protection against Zika virus. *Mol Ther* 28: 1772–1782

Ku MW, Authie P, Bourgine M, Anna F, Noirat A, Moncoq F, Vesin B, Nevo F, Lopez J, Souque P *et al* (2021a) Brain cross-protection against SARS-CoV-2 variants by a lentiviral vaccine in new transgenic mice. *EMBO Mol Med* 13: e14459

Ku MW, Authie P, Nevo F, Souque P, Bourgine M, Romano M, Charneau P, Majlessi L (2021b) Lentiviral vector induces high-quality memory T cells via dendritic cells transduction. *Commun Biol* 4: 713

Ku MW, Bourgine M, Authie P, Lopez J, Nemirov K, Moncoq F, Noirat A, Vesin B, Nevo F, Blanc C *et al* (2021c) Intranasal vaccination with a lentiviral vector protects against SARS-CoV-2 in preclinical animal models. *Cell Host Microbe* 29: 236–249.e6

Ku MW, Charneau P, Majlessi L (2021d) Use of lentiviral vectors in vaccination. *Expert Rev Vaccines* 20: 1571–1586

Larson RC, Kann MC, Bailey SR, Haradhvala NJ, Llopis PM, Bouffard AA, Scarfo I, Leick MB, Grauwet K, Berger TR *et al* (2022) CAR T cell killing requires the IFNgammaR pathway in solid but not liquid tumours. *Nature* 604: 563–570

Lechner M, Liu J, Masterson L, Fenton TR (2022) HPV-associated oropharyngeal cancer: epidemiology, molecular biology and clinical management. *Nat Rev Clin Oncol* 19: 306–327

Liang Z, Tian Y, Cai W, Weng Z, Li Y, Zhang H, Bao Y, Li Y (2017) High-affinity human PD-L1 variants attenuate the suppression of T cell activation. *Oncotarget* 8: 88360–88375

Lopez J, Anna F, Authie P, Pawlik A, Ku MW, Blanc C, Souque P, Moncoq F, Noirat A, Hardy D *et al* (2022) A lentiviral vector encoding fusion of light invariant chain and mycobacterial antigens induces protective CD4(+) T cell immunity. *Cell Rep* 40: 111142

Madsen L, Labrecque N, Engberg J, Dierich A, Svejgaard A, Benoist C, Mathis D, Fugger L (1999) Mice lacking all conventional MHC class II genes. *Proc Natl Acad Sci USA* 96: 10338–10343

Masopust D, Soerens AG (2019) Tissue-resident T cells and other resident leukocytes. *Annu Rev Immunol* 37: 521–546

Mills AA (2017) The chromodomain helicase DNA-binding chromatin remodelers: family traits that protect from and promote cancer. *Cold Spring Harb Perspect Med* 7: a026450

van Montfoort N, Borst L, Korrer MJ, Sluijter M, Marijt KA, Santegoets SJ, van Ham VJ, Ehsan I, Charoentong P, Andre P *et al* (2018) NKG2A blockade potentiates CD8 T cell immunity induced by cancer vaccines. *Cell* 175: 1744–1755.e15

Nemirov K, Bourgine M, Anna F, Wei Y, Charneau P, Majlessi L (2023) Lentiviral vectors as a vaccine platform against infectious diseases. *Pharmaceutics* 15: 846

Odorizzi PM, Pauken KE, Paley MA, Sharpe A, Wherry EJ (2015) Genetic absence of PD-1 promotes accumulation of terminally differentiated exhausted CD8+ T cells. *J Exp Med* 212: 1125–1137

Pajot A, Michel ML, Fazilleau N, Pancre V, Auriault C, Ojcius DM, Lemonnier FA, Lone YC (2004) A mouse model of human adaptive immune functions:

© 2023 The Authors

HLA-A2.1-/HLA-DR1-transgenic H-2 class I–/class II-knockout mice. *Eur J Immunol* 34: 3060–3069

Pascolo S, Bervas N, Ure JM, Smith AG, Lemonnier FA, Perarnau B (1997) HLA-A2.1-restricted education and cytolytic activity of CD8(+) T lymphocytes from beta2 microglobulin (beta2m) HLA-A2.1 monochain transgenic H-2Db beta2m double knockout mice. *J Exp Med* 185: 2043–2051

Pauken KE, Torchia JA, Chaudhri A, Sharpe AH, Freeman GJ (2021) Emerging concepts in PD-1 checkpoint biology. *Semin Immunol* 52: 101480

Pollack SM, Lu H, Gnjatic S, Somaiah N, O'Malley RB, Jones RL, Hsu FJ, Ter Meulen J (2017) First-in-human treatment with a dendritic cell-targeting lentiviral vector-expressing NY-ESO-1, LV305, induces deep, durable response in refractory metastatic synovial sarcoma patient. *J Immunother* 40: 302–306

Ramos da Silva J, Bitencourt Rodrigues K, Formoso Pelegrin G, Silva Sales N, Muramatsu H, de Oliveira Silva M, Porchia B, Moreno ACR, Aps L, Venceslau-Carvalho AA *et al* (2023) Single immunizations of self-amplifying or non-replicating mRNA-LNP vaccines control HPV-associated tumors in mice. *Sci Transl Med* 15: eabn3464

Romero G, von Zastrow M, Friedman PA (2011) Role of PDZ proteins in regulating trafficking, signaling, and function of GPCRs: means, motif, and opportunity. *Adv Pharmacol* 62: 279–314

Rosalik K, Tarney C, Han J (2021) Human papilloma virus vaccination. *Viruses* 13: 1091

Sandoval F, Terme M, Nizard M, Badoual C, Bureau MF, Freyburger L, Clement O, Marcheteau E, Gey A, Fraisse G *et al* (2013) Mucosal imprinting of vaccine-induced CD8(+) T cells is crucial to inhibit the growth of mucosal tumors. *Sci Transl Med* 5: 172ra120

Sanjabi S, Zenewicz LA, Kamanaka M, Flavell RA (2009) Anti-inflammatory and pro-inflammatory roles of TGF-beta, IL-10, and IL-22 in immunity and autoimmunity. *Curr Opin Pharmacol* 9: 447–453

de Sanjose S, Serrano B, Tous S, Alejo M, Lloveras B, Quiros B, Clavero O, Vidal A, Ferrandiz-Pulido C, Pavon MA *et al* (2018) Burden of Human Papillomavirus (HPV)-related cancers attributable to HPVs 6/11/16/18/31/33/45/52 and 58. *JNCI Cancer Spectr* 2: pky045

Schaerli P, Moser B (2005) Chemokines: control of primary and memory T-cell traffic. *Immunol Res* 31: 57–74

Schenkel JM, Fraser KA, Vezys V, Masopust D (2013) Sensing and alarm function of resident memory CD8(+) T cells. *Nat Immunol* 14: 509–513

Schenkel JM, Fraser KA, Beura LK, Pauken KE, Vezys V, Masopust D (2014) T cell memory. Resident memory CD8 T cells trigger protective innate and adaptive immune responses. *Science* 346: 98–101

Seillet C, Brossay L, Vivier E (2021) Natural killers or ILC1s? That is the question. *Curr Opin Immunol* 68: 48–53

Sheng J, Chen Q, Soncin I, Ng SL, Karjalainen K, Ruedl C (2017) A discrete subset of monocyte-derived cells among typical conventional type 2 dendritic cells can efficiently cross-present. *Cell Rep* 21: 1203–1214

Shimizu K, Sugiura D, Okazaki IM, Maruhashi T, Takemoto T, Okazaki T (2021) PD-1 preferentially inhibits the activation of low-affinity T cells. *Proc Natl Acad Sci USA* 118: e2107141118

Shrimali R, Ahmad S, Berrong Z, Okoev G, Matevosyan A, Razavi GSE, Petit R, Gupta S, Mkrtichyan M, Khleif SN (2017) Agonist anti-GITR antibody significantly enhances the therapeutic efficacy of Listeria monocytogenes-based immunotherapy. *J Immunother Cancer* 5: 64

Smalley Rumfield C, Roller N, Pellom ST, Schlom J, Jochems C (2020) Therapeutic vaccines for HPV-associated malignancies. *Immunotargets Ther* 9: 167–200

Somaiah N, Block MS, Kim JW, Shapiro GI, Do KT, Hwu P, Eder JP, Jones RL, Lu H, Ter Meulen JH *et al* (2019) First-in-class, first-in-human study evaluating LV305, a dendritic-cell tropic lentiviral vector, in sarcoma and other solid tumors expressing NY-ESO-1. *Clin Cancer Res* 25: 5808–5817

Steinbach A, Riemer AB (2018) Immune evasion mechanisms of human papillomavirus: an update. *Int J Cancer* 142: 224–229

TheraVectys-Clinical-Trial (2019) Safety, tolerability and immunogenicity induced by the THV01 treatment in patients infected with HIV-1 clade B and treated with highly active antiretroviral therapy (HAART). https://wwwclinicaltrialsregistereu/ctr-search/search?query=2011-006260-522011-006260-52

Vesin B, Lopez J, Noirat A, Authie P, Fert I, Le Chevalier F, Moncoq F, Nemirov K, Blanc C, Planchais C *et al* (2022) An intranasal lentiviral booster reinforces the waning mRNA vaccine-induced SARS-CoV-2 immunity that it targets to lung mucosa. *Mol Ther* 30: 2984–2997

Wieking BG, Vermeer DW, Spanos WC, Lee KM, Vermeer P, Lee WT, Xu Y, Gabitzsch ES, Balcaitis S, Balint JP Jr *et al* (2012) A non-oncogenic HPV 16 E6/E7 vaccine enhances treatment of HPV expressing tumors. *Cancer Gene Ther* 19: 667–674

de Wispelaere M, Ricklin M, Souque P, Frenkiel MP, Paulous S, Garcia-Nicolas O, Summerfield A, Charneau P, Despres P (2015) A lentiviral vector expressing japanese encephalitis virus-like particles elicits broad neutralizing antibody response in pigs. *PLoS Negl Trop Dis* 9: e0004081

Zhang W, Wang H, Sun M, Deng X, Wu X, Ma Y, Li M, Shuoa SM, You Q, Miao L (2020) CXCL5/CXCR2 axis in tumor microenvironment as potential diagnostic biomarker and therapeutic target. *Cancer Commun (Lond)* 40: 69–80

Zhao X, Shan Q, Xue HH (2022) TCF1 in T cell immunity: a broadened frontier. *Nat Rev Immunol* 22: 147–157

Zottnick S, Voss AL, Riemer AB (2020) Inducing immunity where it matters: orthotopic HPV tumor models and therapeutic vaccinations. *Front Immunol* 11: 1750

zur Hausen H (2002) Papillomaviruses and cancer: from basic studies to clinical application. *Nat Rev Cancer* 2: 342–350

License: This is an open access article under the terms of the Creative Commons Attribution License, which permits use, distribution and reproduction in any medium, provided the original work is properly cited.

Expanded View Figures

E6$_{HPV\ 16}$ Native

~~MHQKRTAM~~FQDPQERPRKLPQLCTELQTTIHDIILECVYCKQQLLRREVYDFAFRD**L**CIVYR~~DG~~NPYAVCDKCLKFYSKI
SEYRHYCYSLYGTTLEQQYNKPLCDLLIRCINCQKPL~~CPEEKQRHLDKKQ~~RFHNIRGRWTGRCMSCCR~~SSRTRRETQL~~

E6$_{HPV\ 16}$ in Lenti-HPV

FQDPQERPRKLPQLCTELQTTIHDIILECVYCKQQLLRREVYDFAFRD**G**CIVYRNPYAVCDKCLKFYSKISEYRHYCYSLYG
TTLEQQYNKPLCDLLIRCINCQKPLRFHNIRGRWTGRCMSCCR

E7$_{HPV\ 16}$ Native

~~M~~**H**GDTPTLHEYMLDLQPETTD~~LYCYEQLNDSSEEEDEIDGPAGQAE~~PDRAHYNIVTFCCKCDSTLR**L**CVQSTHVDIRT
LEDLLMGTLGIVCPI~~CSQKP~~

E7$_{HPV\ 16}$ in Lenti-HPV

PGDTPTLHEYMLDLQPETTDPDRAHYNIVTFCCKCDSTLR**R**CVQSTHVDIRTLEDLLMGTLGIVCPI

E6$_{HPV\ 18}$ Native

~~MARFEDPTRR~~PYKLPDLCTELNTSLQDIEITCVYCKTVLELTEVFEFAFKD**L**FVVYRDSIPHAACHK~~CIDFYSRIRELRHYS~~
~~DSVYGDT~~LEKLTNTGLYNLLIRCLRCQK~~PLNP~~AEKLRHLNEKRRFHNIAG~~HYRGQCHSCCNRARQERLQRRRETQV~~

E6$_{HPV\ 18}$ in Lenti-HPV

PYKLPDLCTELNTSLQDIEITCVYCKTVLELTEVFEFAFKD**G**FVVYRDSIPHAACHKLEKLTNTGLYNLLIRCLRCQKAEKLR
HLNEKRRFHNIAG

E7$_{HPV\ 18}$ Native

~~MHGP~~KATLQDIVLHLEPQNEIPVD~~LLCHEQLSD~~SEEENDEIDGVNHQHLPARRAEPQRHTMLCMCCKCEARIKLVVES
SADDLRAFQQLFLNTLSFVCPW~~CASQQ~~

E7$_{HPV\ 18}$ in Lenti-HPV

KATLQDIVLHLEPQNEIPVDSEEENDEIDGVNHQHLPARRAEPQRHTMLCMCCKCEARIKLVVESSADDLRAFQQLFL
NTLSFVCPW

Figure EV1. Protein sequence of each HPV antigen encoded by Lenti-HPV vectors.

Protein sequence of each HPV antigen, the native sequence and that included in the Lenti-HPV vectors. The segments underlined in the native sequences are those deleted in the Lenti-HPV antigen design. The a.a. substitutions are indicated in bold characters on the sequences included in Lenti-HPV. The native E6$_{HPV\ 16}$ sequence has been deleted for the segments 1–8, 63–64, 118–130, 149–158 and substituted at the position 57 of the native sequence (L57G). L57 is part of the binding site to the p53 tumor suppressor protein and activation of telomerase. Note that the deleted 149–158 segment encompasses the PDZ-interacting domain (156–158). The native E7$_{HPV\ 16}$ sequence has been deleted for the a.a. at the position 1 and a.a. segments 22–46, 94–98 and substituted at the positions 2 (H2P), that is, at the binding site to the Rb tumor suppressor protein, and 67 (L67R), that is, at the binding site to CHD4, of the native sequence. The native E6$_{HPV\ 18}$ sequence has been deleted for the a.a. segments 1–10, 68–89, 111–114, 133–158 and substituted at the position 52 of the native sequence (L52R), part of the binding site to the p53 tumor suppressor protein. Note that the deleted 133–158 segment encompasses the PDZ-interacting domain (156–158). The native E7$_{HPV\ 18}$ sequence has been deleted for the a.a. segments 1–4, 25–33, and 101–105.

© 2023 The Authors

Figure EV2. T-cell immunogenicity of various Lenti-HPV against each selected HPV antigen and efficacy of mAb-mediated T subset depletion for functional analysis.

A Comparable immunogenicity of various Lenti-HPV. C57BL/6 mice (*n* = 5/group) were immunized i.m. at day 0 with Ctrl Lenti or each of the Lenti-HPV. On day 14, T splenocyte responses were assessed by IFN-γ ELISPOT after *in vitro* stimulation with a pool of 15-mer peptides spanning the sequence of $E6_{HPV16}$, $E6_{HPV18}$, $E7_{HPV16}$ or $E7_{HPV18}$, as detailed in Fig 2A. Comparable T-cell immunogenicity of the four Lenti-HPVs showed that the position of the antigen in the four poly-antigens encoded by these vectors has no effect on their immunogenicity.

B Efficacy of anti-CD4 or anti-CD8 depletion *in vivo*. Tumor-free C57BL/6 mice (*n* = 2/group) received two i.p. injections of 250 μg anti-CD4 (clone GK1.5), anti-CD8 (clone H35.17.2) or a control Ig isotype 3 days apart. On day 4 after the second injection, the spleens from individual mice were assessed by anti-CD3, anti-CD4, and anti-CD8 mAb staining and cytometry analysis to evaluate the efficacy of T-subset depletion. The results from two individual mice are shown. Note that the mAbs used for depletion and cytometric studies were different to allow the distinction between epitope masking and genuine T-subset depletion.

Source data are available online for this figure.

 © 2023 The Authors

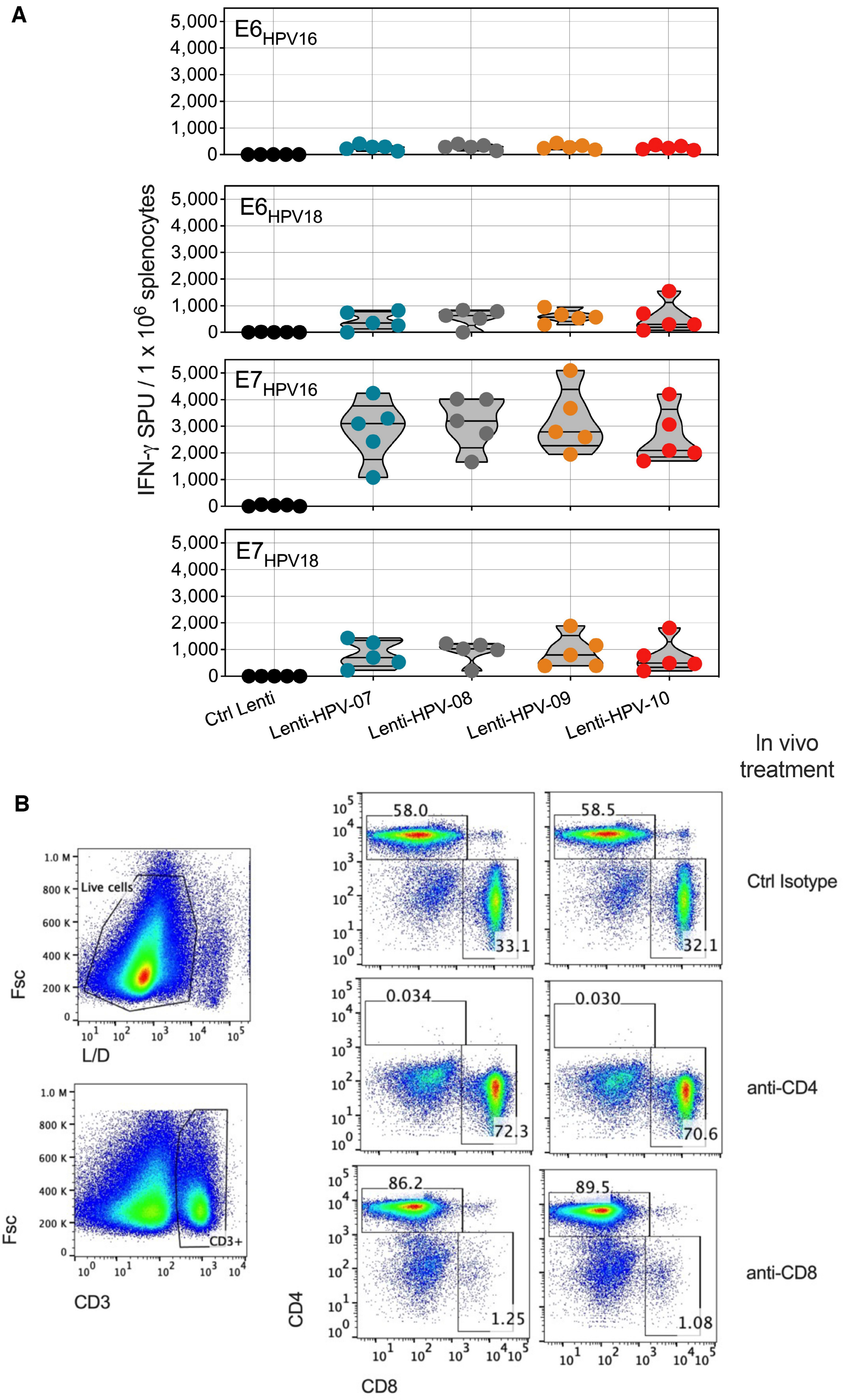
A
E6HPV16
E6HPV18
E7HPV16
E7HPV18
IFN-γ SPU / 1 x 10^6 splenocytes
5,000
4,000
3,000
2,000
1,000
0
Ctrl Lenti
Lenti-HPV-07
Lenti-HPV-08
Lenti-HPV-09
Lenti-HPV-10
B
Live cells
Fsc
L/D
CD3+
CD3
In vivo treatment
58.0
33.1
58.5
32.1
Ctrl Isotype
0.034
72.3
0.030
70.6
anti-CD4
86.2
1.25
89.5
1.08
anti-CD8
CD4
CD8

Figure EV2.

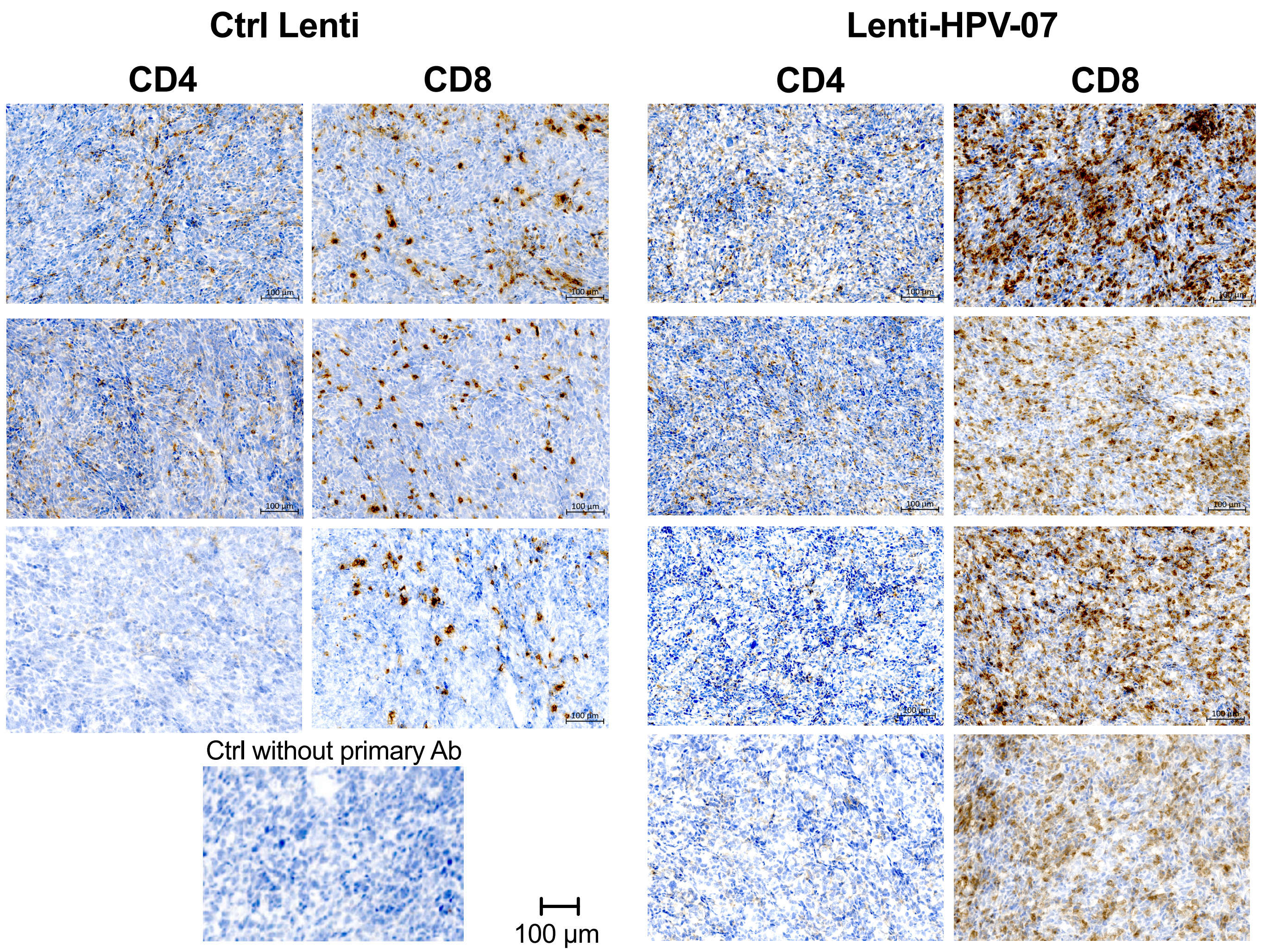

Figure EV3. Comparative immunohistochemistry analysis of tumor infiltrating T cells in Ctrl Lenti- or Lenti-HPV-07-treated, tumor-bearing mice.

C57BL/6 mice were engrafted s.c. with 1×10^6 tumor cells and injected with 1×10^9 TU of Lenti Ctrl or Lenti-HPV-07 ($n = 4$–5). Tumor-infiltrating T cells were studied by CD4 or CD8 immunohistochemistry on day 11 post-vaccination. Tumors from individual mice are shown.

Source data are available online for this figure.

Figure EV4. No role for NK cells in the tumor regression of TC-1 cells induced by Lenti-HPV-07 therapy.

A Timeline of the tumor engraftment, Lenti-HPV-07 treatment and anti-NK1.1 mAb injection. C57BL/6 mice ($n = 7$/group) were engrafted s.c. on the flank with 1×10^6 tumor cells. On day 14 post-engraftment, mice were injected with 1×10^9 TU of Ctrl Lenti or Lenti-HPV-07. From day 13 to 30 mice were treated with a control Ig or anti-NK1.1 mAb by 9 successive i.p. injection of 300 µg/mouse/injection.

B Efficacy of anti-NK1.1 depletion *in vivo* was verified on the splenoctyes of one mouse per group assessed by anti-CD11b and anti-NKp46 mAb staining and cytometry analysis. Note that the mAbs used for NK cell depletion and cytometric studies target distinct markers to ensure genuine NK cell depletion and not epitope masking.

C Spaghettis plots of tumor growth in the mice.

Source data are available online for this figure.

 © 2023 The Authors

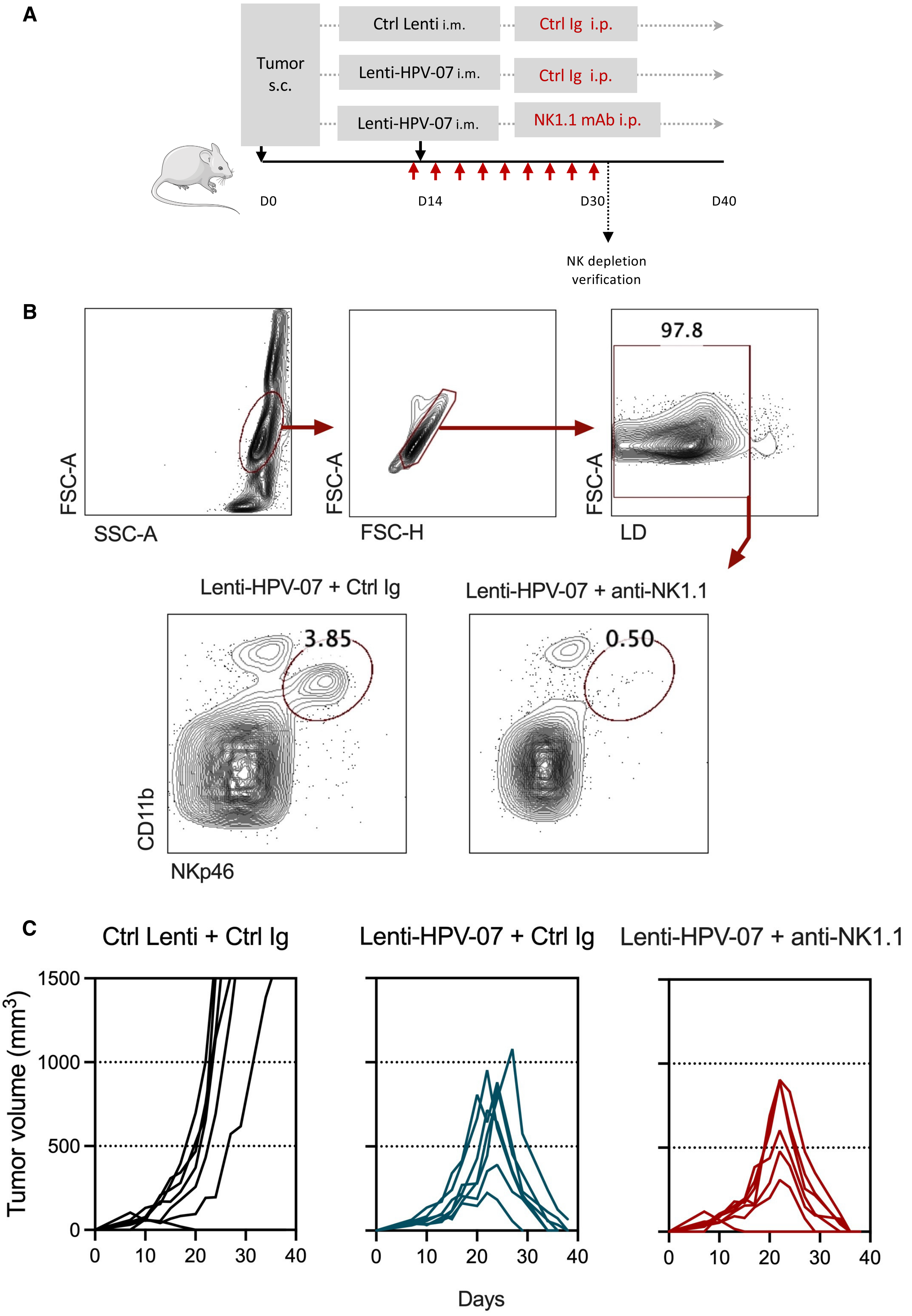
A
Tumor s.c.
Ctrl Lenti i.m.
Ctrl Ig i.p.
Lenti-HPV-07 i.m.
Ctrl Ig i.p.
Lenti-HPV-07 i.m.
NK1.1 mAb i.p.
D0
D14
D30
D40
NK depletion verification
B
97.8
FSC-A
SSC-A
FSC-H
LD
Lenti-HPV-07 + Ctrl Ig
Lenti-HPV-07 + anti-NK1.1
3.85
0.50
CD11b
NKp46
C
Ctrl Lenti + Ctrl Ig
Lenti-HPV-07 + Ctrl Ig
Lenti-HPV-07 + anti-NK1.1
Tumor volume (mm3)
Days

Figure EV4.

Figure EV5. Failure of combinatory anti-PD1 treatment and suboptimal vaccination with Lenti-HPV-07 when the two treatments are given simultaneously.

A Timeline of the tumor engraftment and combinatory treatment with Lenti-HPV-07 vaccination and anti-PD1 injections. C57BL/6 mice (n = 10/group) were engrafted s.c. on the flank with 1×10^6 tumor cells. On day 10 post-engraftment, when the tumor volume reached an average of 100–120 mm^3, mice were injected with a suboptimal dose of 1×10^8 TU Ctrl Lenti or Lenti-HPV-07. Mice were treated with anti-PD1 mAb on the same day and on day 13.

B Spaghettis plots of tumor growth in the mice. The gray arrows indicate the time point at which the vaccine was injected.

C Survival curves of the animals (n = 10/group) shown in (B) followed for 130 days. Statistical significance was determined by Log-rank Mantel-Cox tests (ns, not significant, $**P \leq 0.01$, $***P \leq 0.001$). Note that there is a net tendency to decline in the survival of mice treated with Lenti-HPV-07 + anti-PD1 compared to the mice treated with Lenti-HPV-07 alone, even though this difference did not reach statistical significance. Mice were sacrificed when the size of the tumors reached 1,500 mm^3, in accordance with the defined humane endpoints.

Source data are available online for this figure.

 © 2023 The Authors

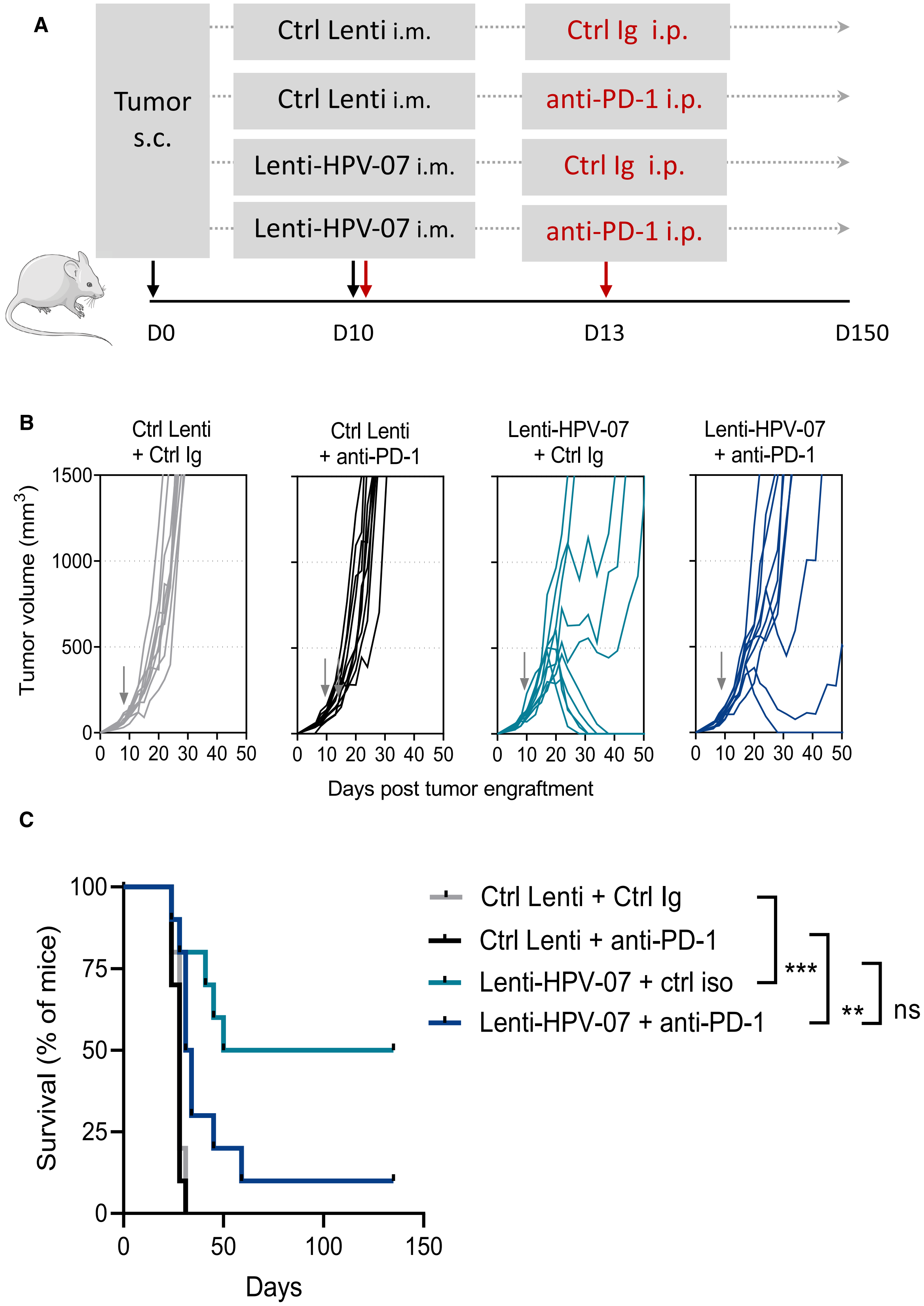
A
Tumor s.c.
Ctrl Lenti i.m.
Ctrl Lenti i.m.
Lenti-HPV-07 i.m.
Lenti-HPV-07 i.m.
Ctrl Ig i.p.
anti-PD-1 i.p.
Ctrl Ig i.p.
anti-PD-1 i.p.
D0
D10
D13
D150
B
Ctrl Lenti + Ctrl Ig
Ctrl Lenti + anti-PD-1
Lenti-HPV-07 + Ctrl Ig
Lenti-HPV-07 + anti-PD-1
Tumor volume (mm3)
Days post tumor engraftment
C
Survival (% of mice)
Days
Ctrl Lenti + Ctrl Ig
Ctrl Lenti + anti-PD-1
Lenti-HPV-07 + ctrl iso
Lenti-HPV-07 + anti-PD-1
***
**
ns

Figure EV5.

Appendix

Supplemental Data

Full Eradication of Pre-clinical Human PapillomaVirus-Induced Tumors by a Lentiviral Vaccine

Table of Contents

Appendix Figure S1. HPV-specific T-splenocyte responses in tumor-free or tumor-bearing mice.

Appendix Figure S2. Decreased proportions of intra-tumoral Treg and unchanged proportions of intra-tumoral B cells in Lenti-HPV-07-vaccinated mice.

Appendix Figure S3. Anti-PD1 treatment does not synergize with suboptimal dose of Lenti-HPV-07 on very large tumors.

Appendix Figure S4. Map of the plasmid encoding various HPV-07 poly-antigenic fusion proteins.

Appendix Table S1. Sequences of primers used in qRT-PCR.

Amino acid sequences of the detoxified HPV antigens.

Amino acid sequences of HPV poly-antigens.

Nucleic sequences of HPV poly-antigens.

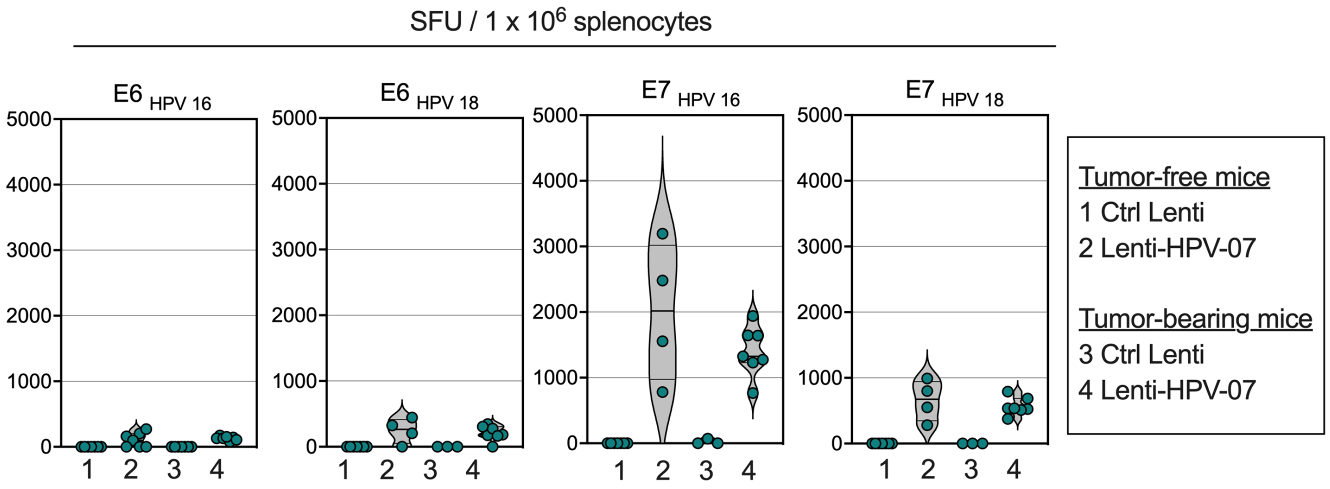

Appendix Figure S1. HPV-specific T-splenocyte responses in tumor-free or tumor-bearing mice. Tumor-free or tumor engrafted C57BL/6 mice (*n* = 3-7/group) were immunized i.m. with Ctrl Lenti or Lenti-HPV-07. On day 14 post-vaccination, T-splenocyte responses were assessed by IFN-γ ELISPOT, as detailed in Figure 2A.

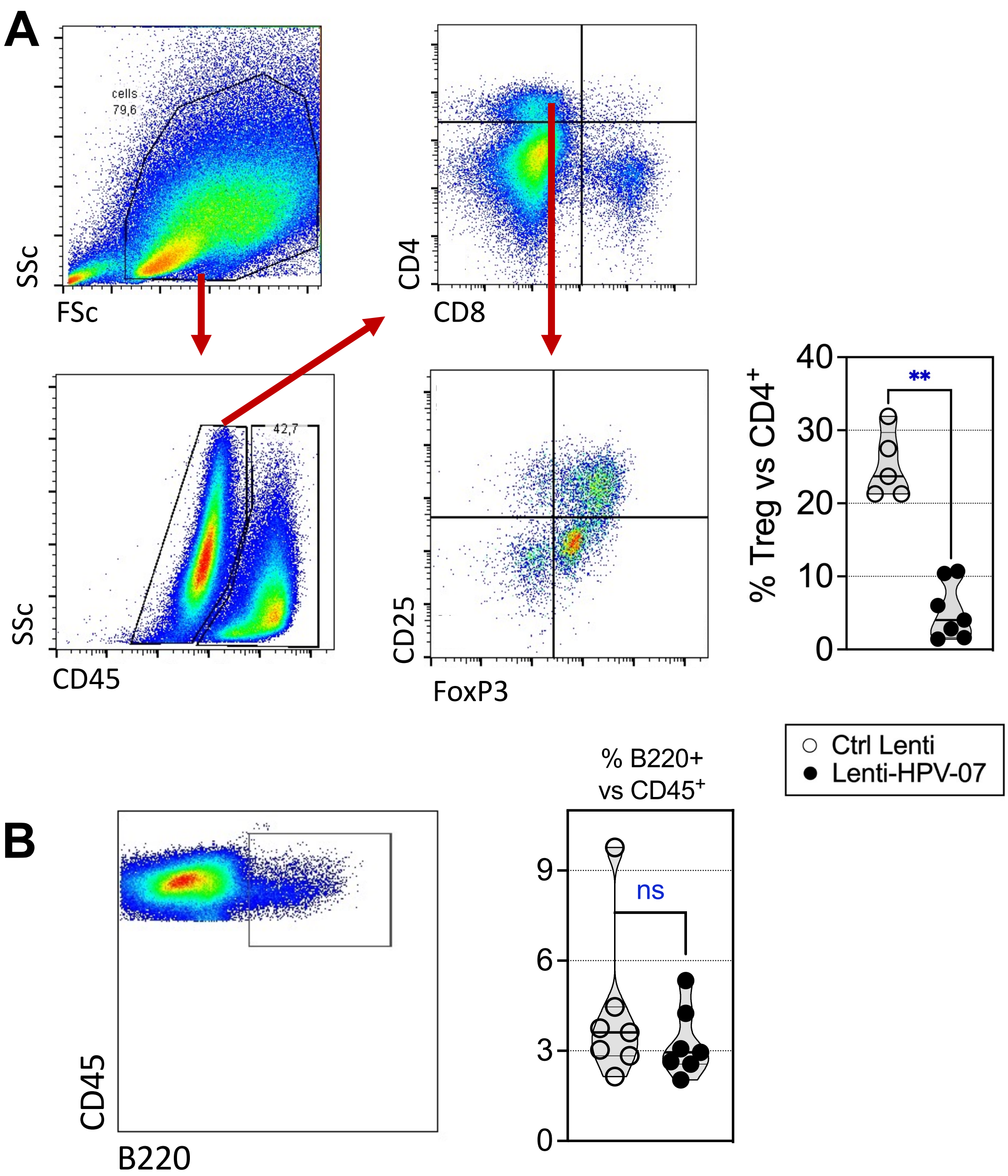

Appendix Figure S2. Decreased proportions of intra-tumoral Treg and unchanged proportions of intra-tumoral B cells in Lenti-HPV-07-vaccinated mice.

(A) Treg cytometric gating strategy and comparative proportions of intra-tumoral Treg populations in Lenti Ctrl- or Lenti-HPV-07-treated C57BL/6 mice studied on day 11 post-vaccination.

(B) Representative dot blot and recapitulative percentages of each subset compared between the two groups. Statistical significance was determined using two-tailed unpaired t tests (ns: not significant, $^{**}p \leq 0.01$).

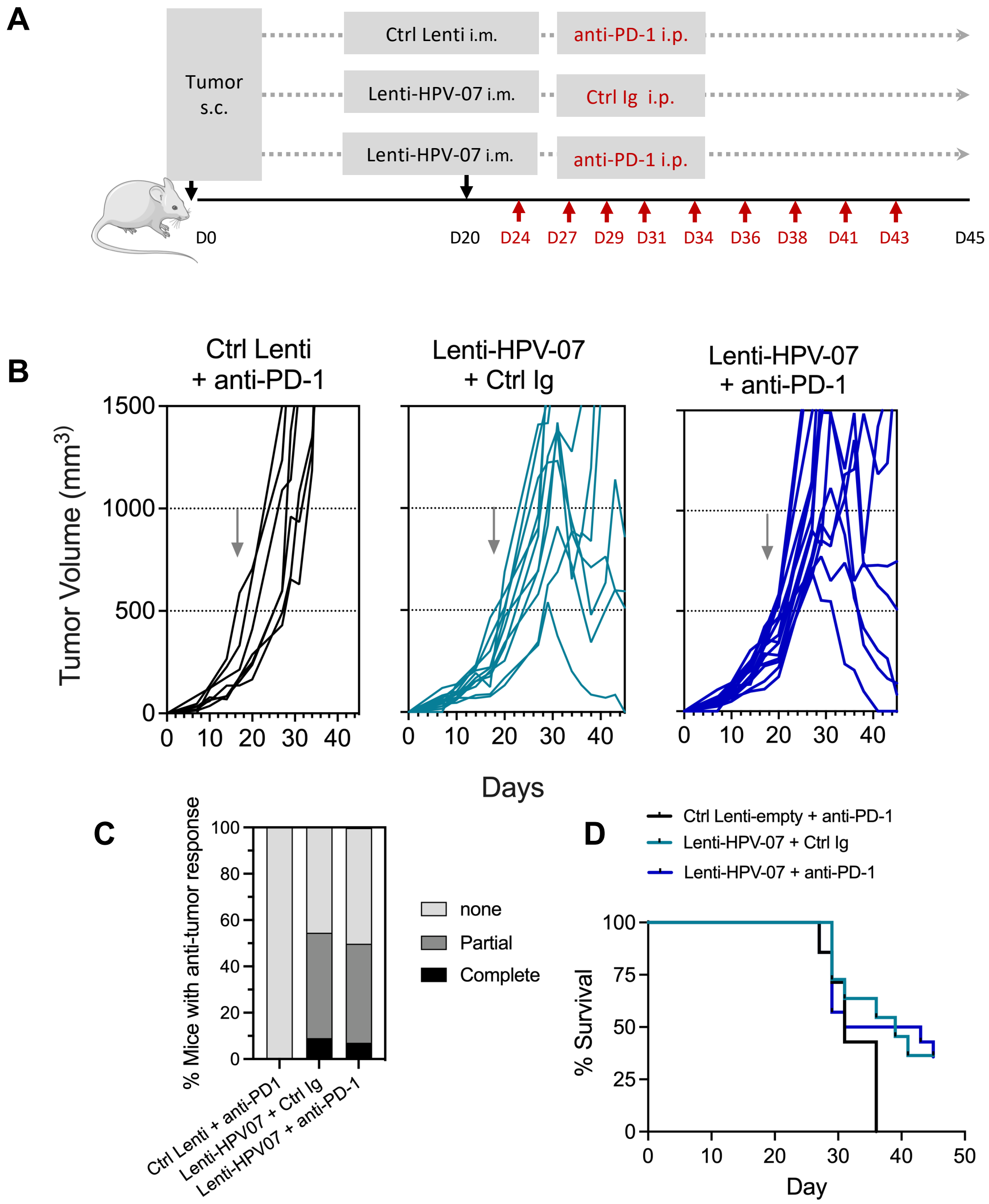

Appendix Figure S3. Anti-PD1 treatment does not synergize with suboptimal dose of Lenti-HPV-07 on very large tumors.

A. Timeline of tumor s.c. engraftment and combinatory Lenti-HPV-07 and anti-PD1 mAb treatment. C57BL/6 mice (n = 7 to 14/group) were engrafted on the flank with 1×10^6 tumor cells. On day 20 post-engraftment, when the tumor volume reached an average of 390 mm^3, mice were treated with the suboptimal dose of 1×10^8 TU of Ctrl Lenti or Lenti-HPV-07. Mice were then treated 2 to 3 times a week with anti-PD1 mAb for a total of nine injections from day 24 to 43.

B. Spaghetti plots of tumor growth. The gray arrows indicate the time point at which Ctrl Lenti or Lenti-HPV-07 were injected.

C. Percentages of mice, without, with partial or complete antitumor response.

D. Survival curves of animals, followed for 45 days.

Mice were sacrificed when the size of the tumors reached 1500 mm^3, in accordance with the defined humane endpoints.

A

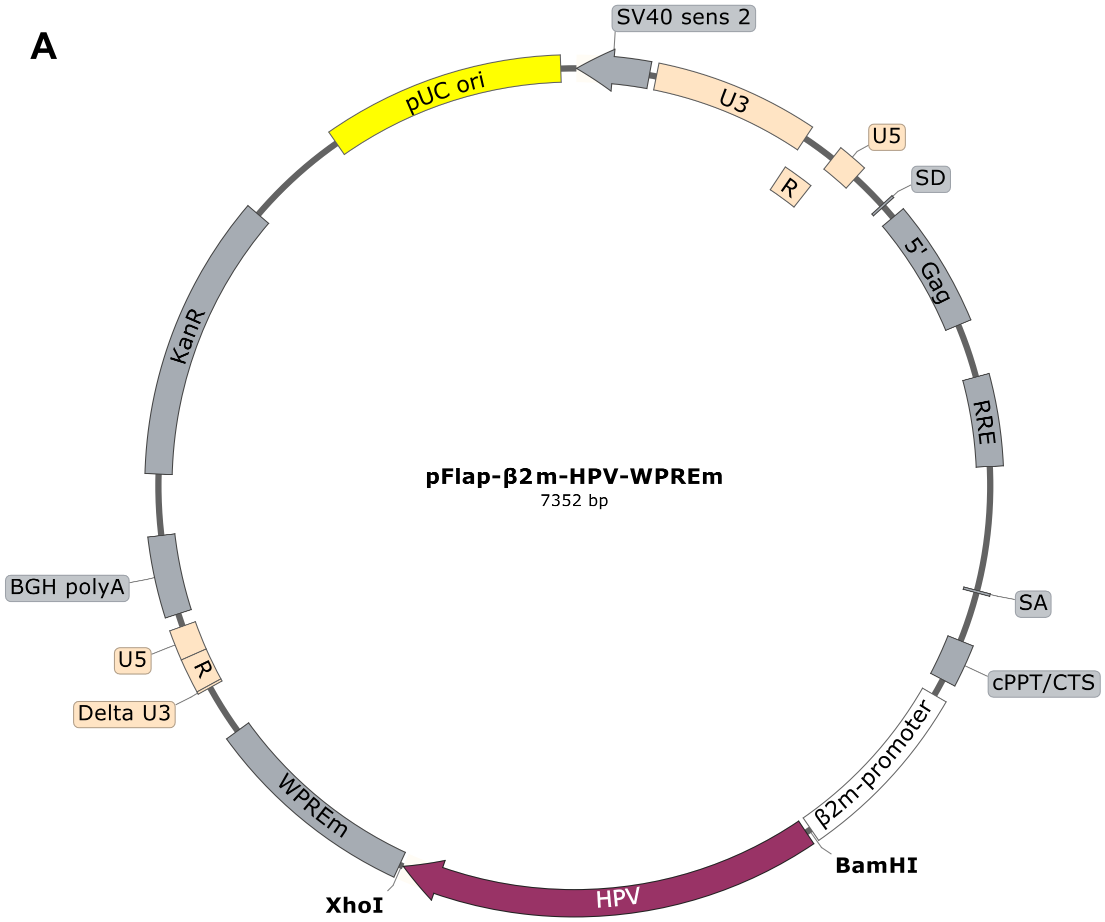

B

$E7_{HPV16}E6_{HPV16}E7_{HPV18}E6_{HPV18}$

PGDTPTLHEYMLDLQPETTDPDRAHYNIVTFCCKCDSTLR**R**CVQSTHV
DIRTLEDLLMGTLGIVCPIasqaFQDPQERPRKLPQLCTELQTTIHDIILE
CVYCKQQLLRREVYDFAFRD**G**CIVYRNPYAVCDKCLKFYSKISEYRHYCY
SLYGTTLEQQYNKPLCDLLIRCINCQKPLRFHNIRGRWTGRCMSCCRsa
gpgpKATLQDIVLHLEPQNEIPVDSEEENDEIDGVNHQHLPARRAEPQ
RHTMLCMCCKCEARIKLVVESSADDLRAFQQLFLNTLSFVCPWvgepg
rtiPYKLPDLCTELNTSLQDIEITCVYCKTVLELTEVFEFAFKD**G**FVVYRDSI
PHAACHKLEKLTNTGLYNLLIRCLRCQKAEKLRHLNEKRRFHNIAG

Appendix Figure S4. Map of the plasmid encoding various HPV-07 poly-antigenic fusion proteins.

(A) The codon-optimized cDNA sequences, encoding various poly-antigens, indicated in Figure 1 were inserted under the β2m promoter in a pFLAP backbone plasmid.

(B) Protein sequence of the HPV-07 antigen, containing 395 aa. The underlined lower-case characters indicate linker sequences between each HPV antigen.

Appendix Table S1. Sequences of primers used in qRT-PCR.

Gene	cDNA accession number	Forward	Reverse	Amplicon (bp)
β2m	NM_009735.3	ATGGGAAGCCGAACATACTG	CAGTCTCAGTGGGGGTGAAT	177
IFNα	NM_010502.2	GGATGTGACCTTCCTCAGACTC	ACCTTCTCCTGCGGGAATCCAA	131
IFNγ	NM_008337.4	TCAAGTGGCATAGATGTGGAAGAA	TGGCTCTGCAGGATTTTCATG	92
TNFα	NM_013693	CATCTTCTCAAAATTCGAGTGACAA	TGGGAGTAGACAAGGTACAACCC	175
TGFβ	NM_011577.2	TGACGTCACTGGAGTTGTACGG	GGTTCATGTCATGGATGGTGC	170
IL-1β	NM_008361.4	TGCCACCTTTTGACAGTGATG	AAGGTCCACGGGAAAGACAC	220
IL-2	NM_008366.3	CCTGAGCAGGATGGAGAATTACA	TCCAGAACATGCCGCAGAG	141
IL-4	NM_021283.2	GTACCAGGAGCCATATCCACG	ACGAGCTCACTCTCTGTGGT	154
IL-5	NM_010558.1	GATGAGGCTTCCTGTCCCTACT	TGACAGGTTTTGGAATAGCATTTCC	130
IL-6	NM_031168.2	CTGCAAGTGCATCATCGTTGTTC	TACCACTTCACAAGTCGGAGGC	116
IL-10	NM_010548.2	CAGTGGAGCAGGTGAAGAGT	GTCCAGCAGACTCAATACACACT	157
IL-12p40	NM_001303244.1	GGAAGCACGGCAGCAGAATA	AACTTGAGGGAGAAGTAGGAATGG	180
IL-18	NM_008360.2	GACAGCCTGTGTTCGAGGATATG	TGTTCTTACAGGAGAGGGTAGAC	159
IL-33	NM_001164724.2	CTACTGCATGAGACTCCGTTCTG	AGAATCCCGTGGATAGGCAGAG	136
CCL2	NM_011333.3	AGGTCCCTGTCATGCTTCTG	TCTGGACCCATTCCTTCTTG	249
CCL3	NM_011337.2	CCTCTGTCAC CTGCTCAACA	GATGAATTGGCGTGGAATCT	163
CCL4	NM_013652.2	TGCAAACCTAACCCCGAGC	TCTGTCTGCCTCTTTTGGTCA	242
CCL5	NM_013653.3	GTGCCCACGTCAAGGAGTAT	GGGAAGCTATACAGGGTCA	185
CCL19	NM_011888.4	CTGCCTCAGATTATCTGCCAT	AGGTAGCGGAAGGCTTTCAC	177
CCL21	NM_002989.4	AAGGCAGTGATGGAGGGG	CGGGGTAAGAACAGGATTG	136
CXCL5	NM_009141.3	GCGTTGTGTTTGCTTAACCG	GAACACTGGCCGTTCTTTCC	242
CXCL9	NM_008599.4	AAAATT TCATCACGCCCTTG	TCTCCAGCTTGGTGAGGTCT	207
CXCL10	NM_021274.2	TGCCGTCATTTTCTGCCTCA	AGGCTCGCAGGGATGATTTC	150
FGF-2	NM_008006.2	AAGCGGCTCTACTGCAAGAA	TGTAACACACTTAGAAGCCAGCA	207
FGF-7	NM_008008.4	CGTGGCAGTTGGAATTGTGG	AGGCAACGAACATTTCCCCT	194
VEGF-C	NM_009506.2	GCTGATGTCTGTCCTGTACCC	AGAAGGTGTTTGTGGCTGCT	239
VCAM-1	NM_011693.3	GACCATGGAGCCTGTCAGTT	CAAACACTTGACCGTGACCG	175
ICAM-1	NM_010493.3	AAGCTGTTTGAGCTGAGCGA	GAGGTCCTTGCCTACTTGCT	165
MCSF	NM_001113529.1	CCTTCTTCGACATGGCTGGG	ATCATCCAGCTGTTCCTGGTC	201
GMCSF	NM_009969.4	GTAGAGGCCATCAAAGAAGCCC	GGTAACTTGTGTTTCACAGTCCG	249
MIF	NM_010798.3	GACTTTTAGCGGCACGAACG	AAGAACAGCGGTGCAGGTAA	238
MMP-2	NM_008610.3	GATAACCTGGATGCCGTCGT	TGGTGTGCAGCGATGAAGAT	188
MMP-9	NM_013599.5	TCTAGGCCCAGAGGTAACCC	TGGAAACTCACACGCCAGAA	240
Col1A1	NM_007742.4	CATGAGCCGAAGCTAACCCC	AGCATACCTCGGGTTTCCAC	209

Amino acid sequences of the detoxified HPV antigens

E6-HPV16

FQDPQERPRKLPQLCTELQTTIHDIILECVYCKQQLLRREVYDFAFRDGCIVYRNPYAVCDKCLKFYSKISEYRHYCYSLYGTTLEQ QYNKPLCDLLIRCINCQKPLRFHNIRGRWTGRCMSCCR

E6-HPV18

PYKLPDLCTELNTSLQDIEITCVYCKTVLELTEVFEFAFKDGFVVYRDSIPHAACHKLEKLTNTGLYNLLIRCLRCQKAEKLRHLNEK RRFHNIAG

E7-HPV16

PGDTPTLHEYMLDLQPETTDPDRAHYNIVTFCCKCDSTLRRCVQSTHVDIRTLEDLLMGTLGIVCPI

E7-HPV18

KATLQDIVLHLEPQNEIPVDSEEENDEIDGVNHQHLPARRAEPQRHTMLCMCCKCEARIKLVVESSADDLRAFQQLFLNTLSFV CPW

Amino acid sequences of HPV poly-antigens

HPV-07

E7-HPV16-E6-HPV16-E7-HPV18-E6-HPV18

MPGDTPTLHEYMLDLQPETTDPDRAHYNIVTFCCKCDSTLRRCVQSTHVDIRTLEDLLMGTLGIVCPIasqaFQDPQERPRKLPQ LCTELQTTIHDIILECVYCKQQLLRREVYDFAFRDGCIVYRNPYAVCDKCLKFYSKISEYRHYCYSLYGTTLEQQYNKPLCDLLIRCIN CQKPLRFHNIRGRWTGRCMSCCRsagpgpKATLQDIVLHLEPQNEIPVDSEEENDEIDGVNHQHLPARRAEPQRHTMLCMCC KCEARIKLVVESSADDLRAFQQLFLNTLSFVCPWvgepgrtiPYKLPDLCTELNTSLQDIEITCVYCKTVLELTEVFEFAFKDGFVVYR DSIPHAACHKLEKLTNTGLYNLLIRCLRCQKAEKLRHLNEKRRFHNIAGh

HPV-08

E6-HPV16-E6-HPV18-E7-HPV16-E7-HPV18

MFQDPQERPRKLPQLCTELQTTIHDIILECVYCKQQLLRREVYDFAFRDGCIVYRNPYAVCDKCLKFYSKISEYRHYCYSLYGTTLE QQYNKPLCDLLIRCINCQKPLRFHNIRGRWTGRCMSCCRgpddPYKLPDLCTELNTSLQDIEITCVYCKTVLELTEVFEFAFKDGF VVYRDSIPHAACHKLEKLTNTGLYNLLIRCLRCQKAEKLRHLNEKRRFHNIAGPGDTPTLHEYMLDLQPETTDPDRAHYNIVTFC CKCDSTLRRCVQSTHVDIRTLEDLLMGTLGIVCPIgpddKATLQDIVLHLEPQNEIPVDSEEENDEIDGVNHQHLPARRAEPQRH TMLCMCCKCEARIKLVVESSADDLRAFQQLFLNTLSFVCPWa

HPV-09

E6-HPV18-E6-HPV16-E7-HPV18-E7-HPV16

MrrPYKLPDLCTELNTSLQDIEITCVYCKTVLELTEVFEFAFKDGFVVYRDSIPHAACHKLEKLTNTGLYNLLIRCLRCQKAEKLRHL NEKRRFHNIAGhFQDPQERPRKLPQLCTELQTTIHDIILECVYCKQQLLRREVYDFAFRDGCIVYRNPYAVCDKCLKFYSKISEYRH YCYSLYGTTLEQQYNKPLCDLLIRCINCQKPLRFHNIRGRWTGRCMSCCRsagpgpKATLQDIVLHLEPQNEIPVDSEEENDEIDG VNHQHLPARRAEPQRHTMLCMCCKCEARIKLVVESSADDLRAFQQLFLNTLSFVCPWagPGDTPTLHEYMLDLQPETTDPDR AHYNIVTFCCKCDSTLRRCVQSTHVDIRTLEDLLMGTLGIVCPIa

HPV-10

E7-HPV18-E6-HPV18-E7-HPV16-E6-HPV16

MgpKATLQDIVLHLEPQNEIPVDSEEENDEIDGVNHQHLPARRAEPQRHTMLCMCCKCEARIKLVVESSADDLRAFQQLFLNT LSFVCPgepgrtiPYKLPDLCTELNTSLQDIEITCVYCKTVLELTEVFEFAFKDGFVVYRDSIPHAACHKLEKLTNTGLYNLLIRCLRCQ KAEKLRHLNEKRRFHNIAGPGDTPTLHEYMLDLQPETTDPDRAHYNIVTFCCKCDSTLRRCVQSTHVDIRTLEDLLMGTLGIVCP IasqaFQDPQERPRKLPQLCTELQTTIHDIILECVYCKQQLLRREVYDFAFRDGCIVYRNPYAVCDKCLKFYSKISEYRHYCYSLYGT TLEQQYNKPLCDLLIRCINCQKPLRFHNIRGRWTGRCMSCCRs

Nucleic sequences of HPV poly-antigens

ggatcc : restriction enzyme BamHI
GCCACC : Kozak *sequence*
ATG : initiation codon
TGATAA : Double STOP codon
ctcgag : restriction enzyme XhoI

Codon optimized **HPV-07** (E7-HPV16-E6-HPV16-E7-HPV18-E6-HPV18) sequence :

ggatccGCCACCATGCCCGGAGACACCCCCACCCTGCACGAATACATGCTGGACCTGCAGCCCGAAACCACCGACCCCGAC
CGCGCTCACTACAACATCGTTACATTCTGTTGTAAATGCGACTCCACCCTGAGAAGATGCGTGCAGTCCACCCACGTGGAC
ATCAGGACCCTGGAGGACCTCCTCATGGGAACCCTGGGTATCGTCTGCCCCATCgcctcccaggctTTTCAGGACCCCCAGGA
AAGGCCCAGGAAGTTGCCCCAGCTCTGCACCGAACTGCAGACCACCATTCATGACATCATCCTCGAATGCGTGTACTGCA
AGCAGCAGCTCCTGAGGAGGGAGGTGTACGATTTCGCCTTCAGAGACGGCTGTATCGTCTACAGGAACCCCTATGCCGTC
TGCGACAAATGCCTGAAGTTTTATTCCAAGATCTCCGAGTACAGGCACTATTGCTACAGCCTGTATGGACCACCCTGGAG
CAGCAGTACAACAAGCCCCTGTGCGACCTCCTGATCAGGTGCATCAACTGCCAGAAGCCCCTGAGGTTCCACAACATCCG
CGGCAGGTGGACCGGAAGGTGCATGTCCTGCTGCAGGtccgccggccccggacctAAAGCCACCCTCCAGGACATCGTTCTCCA
CCTGGAGCCCCAGAACGAGATCCCCGTGGACTCAGAAGAGGAGAACGACGAGATCGACGGCGTCAACCACCAGCACCTG
CCCGCTCGCAGAGCCGAACCCCAGAGACACACCATGCTCTGCATGTGCTGCAAATGCGAAGCCCGGATTAAGTTGGTGGT
GGAAAGCAGCGCCGACGATCTGAGGGCCTTCCAGCAGCTCTTCCTCAACACCCTGTCCTTCGTGTGCCCCTGGgtgggcgag
cccggtagaaccatcCCCTACAAGCTGCCCGATCTGTGCACAGAGCTGAACACCTCCCTGCAGGACATCGAGATCACCTGCGTC
TACTGCAAGACCGTGCTGGAACTGACCGAGGTGTTCGAATTCGCCTTCAAGGACGGCTTCGTGGTGTACAGGGACAGCAT
TCCCCACGCCGCCTGCCATAAGCTGGAGAAACTGACCAACACCGGACTGTATAACCTGCTGATCAGGTGTCTGAGGTGCC
AGAAGGCAGAGAAACTGAGACATCTGAACGAGAAAAGGAGGTTCCACAATATTGCCGGGcacTGATAActcgag

Codon optimized **HPV-08** (E6-HPV16-E6-HPV18-E7-HPV16-E7-HPV18) sequence :

ggatccGCCACCATGTTCCAGGACCCCCAGGAGAGGCCCCGGAAGTTGCCCCAGCTGTGCACCGAGCTGCAGACCACCATC
CACGACATCATCCTCGAATGCGTGTACTGCAAGCAGCAGCTGCTGAGGAGGGAGGTGTATGACTTTGCCTTCAGAGACG
GATGCATTGTCTACAGGAACCCCTACGCCGTGTGCGACAAATGCCTGAAGTTCTACTCCAAGATCAGCGAGTACAGGCAC
TACTGCTACTCCCTGTACGGCACCACCCTCGAACAGCAGTACAACAAACCCCTGTGCGACCTCCTGATTAGGTGCATCAAC
TGCCAGAAGCCCCTCAGGTTCCACAACATCCGCGGCCGCTGGACCGGCCGATGCATGTCTTGCTGCAGGggccccgacgacC
CCTACAAGCTCCCCGACCTGTGCACCGAACTCAACACCTCCCTGCAGGACATCGAGATCACCTGCGTGTATTGCAAGACCG
TGCTGGAGCTGACCGAGGTTTTCGAATTTGCCTTTAAGGACGGCTTCGTCGTGTATAGGGACTCCATCCCCCACGCCGCCT
GCCATAAGCTGGAGAAGCTCACCAACACCGGACTGTATAATCTGCTGATCAGGTGCCTCAGGTGCCAGAAGGCAGAAAA
GCTGAGGCATCTCAACGAGAAGCGCCGGTTCCACAATATTGCCGGCCCCGGAGACACCCCCACACTCCATGAGTACATGC
TCGACCTGCAGCCCGAAACCACCGACCCCGACAGAGCCCACTACAACATCGTGACCTTCTGCTGCAAGTGCGACTCCACCC
TGAGAAGATGCGTGCAGTCCACCCACGTGGACATCCGCACACTCGAAGACCTGCTGATGGGAACCCTGGGCATCGTGTG
CCCCATCggccccgatgacAAGGCCACCTTGCAGGACATCGTGCTGCACCTGGAACCACAGAACGAGATCCCCGTCGACTCCG
AAGAAGAAAACGACGAAATCGACGGAGTGAATCACCAGCACCTGCCCGCCAGAAGGGCCGAGCCTCAGAGACACACCA
TGCTCTGCATGTGCTGCAAATGCGAAGCCAGGATTAAGCTGGTGGTGGAGAGCAGCGCCGACGACCTGAGGGCCTTCCA
GCAGCTCTTCCTGAACACACTGTCCTTCGTGTGCCCCTGGgccTGATAActcgag

Codon optimized **HPV-09** (E6-HPV18-E6-HPV16-E7-HPV18-E7-HPV16) sequence :

ggatccGCCACCATGaggcggCCCTACAAGCTGCCCGACCTGTGCACCGAGCTGAACACCTCCCTGCAGGACATCGAGATCAC
CTGCGTGTACTGCAAGACCGTGCTGGAGCTGACCGAGGTGTTCGAATTCGCATTCAAGGACGGATTCGTCGTGTATAGG
GACAGCATTCCACACGCCGCCTGCCACAAGCTGGAGAAATTGACTAACACCGGACTGTATAATCTGCTGATCCGGTGCCT
GAGGTGTCAGAAGGCCGAGAAGCTGAGGCATCTGAACGAGAAAAGGAGATTCCACAATATCGCCGGAcacTTCCAGGAC
CCCCAGGAGAGGCCCAGGAAACTGCCCCAGTTGTGCACCGAGCTCCAGACAACCATCCACGACATCATCCTGGAGTGCGT
GTACTGTAAGCAGCAGTTGCTGAGGAGAGAGGTGTATGACTTCGCCTTCAGAGACGGATGCATTGTCTATAGGAACCCCT
ACGCCGTGTGCGACAAGTGCCTGAAGTTCTACTCCAAGATCAGTGAGTACAGGCATTACTGCTACAGCCTGTATGGAACC

ACACTGGAACAGCAGTACAACAAGCCCCTGTGCGACCTCCTGATTAGGTGCATCAACTGCCAGAAGCCCCTCAGGTTCCA
CAACATCCGGGGCAGGTGGACCGGAAGGTGCATGTCCTGCTGCAGGtccgccggccccggacctAAAGCCACCCTCCAGGACA
TCGTGCTGCACCTGGAGCCCCAGAACGAGATCCCCGTCGACTCAGAGGAGGAGAACGACGAAATTGACGGCGTCAACCA
CCAGCACCTGCCCGCTCGCAGAGCCGAACCCCAGAGACACACCATGCTCTGCATGTGCTGCAAATGCGAGGCCCGGATTA
AGCTGGTGGTGGAGAGCTCCGCCGACGATCTGAGAGCCTTCCAGCAGCTCTTCCTGAACACCCTGTCCTTCGTGTGCCCCT
GGgccggtCCCGGTGACACACCTACCCTGCACGAGTACATGCTCGATCTGCAGCCCGAGACCACCGACCCCGATCGCGCAC
ACTACAACATCGTGACCTTCTGCTGCAAATGTGACAGCACCCTGAGACGGTGCGTCCAGTCCACCCACGTTGACATCCGCA
CCCTCGAAGACCTGCTCATGGGAACCCTGGGCATCGTGTGCCCCATCgccTGATAActcgag

Codon optimized **HPV-10** (E7-HPV18-E6-HPV18-E7-HPV16-E6-HPV16) sequence ：

ggatccGCCACCATGggccctAAGGCCACCCTGCAGGACATCGTGCTGCACTTGGAGCCCCAGAACGAGATCCCCGTGGACA
GCGAGGAGGAGAACGACGAAATCGACGGCGTGAACCACCAGCACCTGCCCGCAAGAAGGGCCGAACCCCAGAGGCACA
CCATGCTCTGCATGTGCTGCAAATGCGAGGCCAGGATCAAGCTGGTGGTGGAAAGCAGCGCCGACGATCTGAGGGCATT
CCAGCAGCTGTTCCTGAACACCCTCTCCTTCGTGTGCCCTggggaacccggcaggaccatcCCCTATAAACTGCCCGACCTCTGCA
CCGAGCTGAACACCTCCCTGCAGGACATTGAGATCACCTGCGTCTACTGCAAAACCGTCCTGGAACTGACCGAGGTGTTC
GAGTTCGCCTTCAAAGACGGCTTCGTCGTGTACAGGGACAGCATCCCCCACGCCGCCTGCCATAAGCTGGAGAAACTGAC
CAACACCGGCCTGTACAACCTGCTGATCCGGTGCCTGAGATGTCAGAAGGCCGAGAAACTGAGGCACCTCAACGAGAAA
AGGAGATTCCACAATATTGCCGGGCCCGGCGACACCCCAACCCTGCACGAATACATGCTCGACCTGCAGCCCGAAACCAC
CGACCCCGACAGAGCCCACTACAACATCGTGACCTTCTGCTGCAAGTGCGACTCCACCCTGAGAAGATGCGTGCAGTCCA
CCCACGTGGACATCCGCACACTCGAAGACCTGCTGATGGGAACCCTGGGCATCGTGTGCCCCATCgcttcccaggccTTTCAG
GACCCCCAGGAACGGCCAAGAAAGCTGCCCCAGCTCTGCACCGAACTGCAGACCACCATCCACGACATCATCCTGGAATG
CGTCTACTGTAAGCAGCAGTTGCTGAGGAGGGAGGTGTATGATTTCGCCTTCAGAGACGGCTGCATCGTCTACAGGAACC
CCTACGCCGTGTGCGACAAATGCCTGAAGTTCTACTCCAAGATCTCCGAATACAGACACTATTGCTACAGCCTGTACGGCA
CCACCCTCGAACAGCAGTACAACAAACCCCTGTGCGACCTCCTGATCAGGTGCATCAACTGCCAGAAGCCCCTCCGGTTCC
ACAACATCCGAGGAAGATGGACCGGCCGGTGCATGTCCTGCTGCAGGtccTGATAActcgag
