# Supplementary material for: Full eradication of pre‐clinical human papilloma virus‐induced tumors by a lentiviral vaccine
Source: EMBO Mol Med. 2023 Sep 7;15(10):e17723. doi: 10.15252/emmm.202317723 (PMC10565635; doi:10.15252/emmm.202317723)
Supplement: Supplementary file 12 [file EMMM-15-e17723-s005.pdf]

EMBO Molecular Medicine

Full Eradication of Pre-clinical Human PapillomaVirus-Induced Tumors by a Lentiviral Vaccine

Laëtitia Douguet, Ingrid Fert, Jodie Lopez, Benjamin Vesin, Fabien Le Chevalier, Fanny Moncoq, Pierre Authié, Trang-My Nguyen, Amandine Noirat, Fabien Névo, Catherine Blanc, Maryline Bourgine, David Hardy, Francois ANNA, Pierre Charneau, and Laleh Majlessi
DOI: 10.15252/emmm.202317723

Corresponding author(s): Laleh Majlessi (laleh.majlessi@pasteur.fr)

Review Timeline:

Submission Date:	20th Mar 23
Editorial Decision:	26th Apr 23
Revision Received:	17th Jul 23
Editorial Decision:	14th Aug 23
Revision Received:	22nd Aug 23
Accepted:	23rd Aug 23

Editor: Zeljko Durdevic

Transaction Report:

(Note: With the exception of the correction of typographical or spelling errors that could be a source of ambiguity, letters and reports are not edited. Depending on transfer agreements, referee reports obtained elsewhere may or may not be included in this compilation. Referee reports are anonymous unless the Referee chooses to sign their reports.)

26th Apr 2023

Dear Dr. Majlessi,

Thank you for the submission of your manuscript to EMBO Molecular Medicine. We have now received feedback from the two reviewers who agreed to evaluate your manuscript. As you will see from the reports below, both referees recognize potential interest of the study but also raise serious concerns that should be addressed in a major revision of the current manuscript. If you would like to discuss further the points raised by the referees, I am available to do so via email or video. Let me know if you are interested in this option.

Further consideration of a revision that addresses reviewers' concerns in full will entail a second round of review. EMBO Molecular Medicine encourages a single round of revision only and therefore, acceptance or rejection of the manuscript will depend on the completeness of your responses included in the next, final version of the manuscript. For this reason, and to save you from any frustrations in the end, I would strongly advise against returning an incomplete revision.

We would welcome the submission of a revised version within three months for further consideration. Please let us know if you require longer to complete the revision.

I look forward to receiving your revised manuscript.

Yours sincerely,

Zeljko Durdevic

Zeljko Durdevic
Editor
EMBO Molecular Medicine

*****

When submitting your revised manuscript, please carefully review the instructions that follow below. We perform an initial quality control of all revised manuscripts before re-review; failure to include requested items will delay the evaluation of your revision.

We require:

1) A .docx formatted version of the manuscript text (including legends for main figures, EV figures and tables). Please make sure that the changes are highlighted to be clearly visible.

2) Individual production quality figure files as .eps, .tif, .jpg (one file per figure). For guidance, download the 'Figure Guide PDF': (https://www.embopress.org/page/journal/17574684/authorguide#figureformat).

3) A .docx formatted letter INCLUDING the reviewers' reports and your detailed point-by-point responses to their comments. As part of the EMBO Press transparent editorial process, the point-by-point response is part of the Review Process File (RPF), which will be published alongside your paper.

4) A complete author checklist, which you can download from our author guidelines (https://www.embopress.org/page/journal/17574684/authorguide#submissionofrevisions). Please insert information in the checklist that is also reflected in the manuscript. The completed author checklist will also be part of the RPF.

5) Please note that all corresponding authors are required to supply an ORCID ID for their name upon submission of a revised manuscript.

6) It is mandatory to include a 'Data Availability' section after the Materials and Methods. Before submitting your revision, primary

datasets produced in this study need to be deposited in an appropriate public database, and the accession numbers and database listed under 'Data Availability'. Please remember to provide a reviewer password if the datasets are not yet public (see https://www.embopress.org/page/journal/17574684/authorguide#dataavailability).

In case you have no data that requires deposition in a public database, please state so in this section. Note that the Data Availability Section is restricted to new primary data that are part of this study.

7) For data quantification: please specify the name of the statistical test used to generate error bars and P values, the number (n) of independent experiments (specify technical or biological replicates) underlying each data point and the test used to calculate p-values in each figure legend. The figure legends should contain a basic description of n, P and the test applied. Graphs must include a description of the bars and the error bars (s.d., s.e.m.). See also 'Figure Legend' guidelines: https://www.embopress.org/page/journal/17574684/authorguide#figureformat

8) At EMBO Press we ask authors to provide source data for the main manuscript figures. Our source data coordinator will contact you to discuss which figure panels we would need source data for and will also provide you with helpful tips on how to upload and organize the files.

9) Our journal encourages inclusion of *data citations in the reference list* to directly cite datasets that were re-used and obtained from public databases. Data citations in the article text are distinct from normal bibliographical citations and should directly link to the database records from which the data can be accessed. In the main text, data citations are formatted as follows: "Data ref: Smith et al, 2001" or "Data ref: NCBI Sequence Read Archive PRJNA342805, 2017". In the Reference list, data citations must be labeled with "[DATASET]". A data reference must provide the database name, accession number/identifiers and a resolvable link to the landing page from which the data can be accessed at the end of the reference. Further instructions are available at .

10) We replaced Supplementary Information with Expanded View (EV) Figures and Tables that are collapsible/expandable online. A maximum of 5 EV Figures can be typeset. EV Figures should be cited as 'Figure EV1, Figure EV2" etc... in the text and their respective legends should be included in the main text after the legends of regular figures.

- For the figures that you do NOT wish to display as Expanded View figures, they should be bundled together with their legends in a single PDF file called *Appendix*, which should start with a short Table of Content. Appendix figures should be referred to in the main text as: "Appendix Figure S1, Appendix Figure S2" etc.

- Additional Tables/Datasets should be labeled and referred to as Table EV1, Dataset EV1, etc. Legends have to be provided in a separate tab in case of .xls files. Alternatively, the legend can be supplied as a separate text file (README) and zipped together with the Table/Dataset file.

See detailed instructions here:

.

11) The paper explained: EMBO Molecular Medicine articles are accompanied by a summary of the articles to emphasize the major findings in the paper and their medical implications for the non-specialist reader. Please provide a draft summary of your article highlighting

- the medical issue you are addressing,

- the results obtained and

- their clinical impact.

This may be edited to ensure that readers understand the significance and context of the research. Please refer to any of our published articles for an example.

12) For more information: There is space at the end of each article to list relevant web links for further consultation by our readers. Could you identify some relevant ones and provide such information as well? Some examples are patient associations, relevant databases, OMIM/proteins/genes links, author's websites, etc...

13) Author contributions: You will be asked to provide CRediT (Contributor Role Taxonomy) terms in the submission system. These replace a narrative author contribution section in the manuscript.

14) A Conflict of Interest statement should be provided in the main text.

15) Every published paper now includes a 'Synopsis' to further enhance discoverability. Synopses are displayed on the journal webpage and are freely accessible to all readers. They include a short stand first (maximum of 300 characters, including space) as well as 2-5 one-sentences bullet points that summarizes the paper. Please write the bullet points to summarize the key NEW findings. They should be designed to be complementary to the abstract - i.e. not repeat the same text. We encourage inclusion of key acronyms and quantitative information (maximum of 30 words / bullet point). Please use the passive voice. Please attach these in a separate file or send them by email, we will incorporate them accordingly.

Please also suggest a striking image or visual abstract to illustrate your article as a PNG file 550 px wide x 300-600 px high.

EMBO Molecular Medicine has a "scooping protection" policy, whereby similar findings that are published by others during review or revision are not a criterion for rejection. Should you decide to submit a revised version, I do ask that you get in touch after three months if you have not completed it, to update us on the status.

Please note: When submitting your revision you will be prompted to enter your funding and payment information. This will allow Wiley to send you a quote for the article processing charge (APC) in case of acceptance. This quote takes into account any reduction or fee waivers that you may be eligible for. Authors do not need to pay any fees before their manuscript is accepted and transferred to the publisher.

EMBO Press participates in many Publish and Read agreements that allow authors to publish Open Access with reduced/no publication charges. Check your eligibility: https://authorservices.wiley.com/author-resources/Journal-Authors/open-access/affiliation-policies-payments/index.html

***** Reviewer's comments *****

Referee #1 (Comments on Novelty/Model System for Author):

In this manuscript, Douguet et al studies the therapeutic efficacy of a non-integrative lentiviral vector encoding E6 and E7 oncoproteins of HPV16 and 18 genotypes (Lenti-HPV-07). They demonstrate that a single i.m. administration of the virus induced full eradication of HPV-induced tumors in 100% of mice. The authors have characterized in depth the phenotype and function of the generated T cells and found a profound remodelling of the tumor microenvirnment towards an inflammatory state, with an important increase in CD8 T cell infiltration. The therapeutic vaccine significantly reduced lung metastases and induced a long lasting immune response. A synergistic effect with anti-PD1 therapy is also claimed. The article is well written and organized, and the results support the conclusions of the work. There are some issues that should be addressed before its acceptance for publication.

Authors should avoid expressions indicating a superiority that has not been completely verified, such as those expressed in lines 377 "The only immunotherapy described as effective in eradicating ....." or line 421: "However, given the superiority of the results obtained with Lenti-HPV...". There are previous works on therapeutic vaccinations using E7 containing fusion proteins with Hsp65 (Chu NR, et al. Cell Stress Chaperones. 2000. PMID: 11189443), EDA fusion proteins (Arribillaga et al, J Immunother Cancer. 2020 PMID: 32581060), peptides (Domingos-Pereira J Immunother Cancer 2019 PMID: 31060612), or non replicating mRNA-LNP vaccines (Ramos da Silva, Sci Transl Med. 2023. PMID: 36867683) among many others, showing therapeutic efficiencies reaching 100% cures in similar tumor models. In the same line, as it is discussed in the "limitations of the study section", preclinical models do not adequately recapitulate or anticipate the results obtained in clinical trials.
In this study, the tumor model used is based on the administration of TC1 cells (s.c. or i.v in some settings). There are other more aggressive models, such as the one based on the administration of TC-1 P3 (A15) cells, with low expression of MHC-I, or the orthotopic tumor models (used in some of the above indicated publications) that could also allow a more in-depth analysis of the efficacy of the vaccine. This fact should also be mentioned among the limitations of the study.

Referee #1 (Remarks for Author):

In this manuscript, Douguet et al studies the therapeutic efficacy of a non-integrative lentiviral vector encoding E6 and E7 oncoproteins of HPV16 and 18 genotypes (Lenti-HPV-07). They demonstrate that a single i.m. administration of the virus induced full eradication of HPV-induced tumors in 100% of mice. The authors have characterized in depth the phenotype and function of the generated T cells and found a profound remodelling of the tumor microenvirnment towards an inflammatory state, with an important increase in CD8 T cell infiltration. The therapeutic vaccine significantly reduced lung metastases and induced a long lasting immune response. A synergistic effect with anti-PD1 therapy is also claimed. The article is well written and organized, and the results support the conclusions of the work. There are some issues that should be addressed before its acceptance for publication.

1. The first is a formal aspect. Authors should avoid expressions indicating a superiority that has not been completely verified, such as those expressed in lines 377 "The only immunotherapy described as effective in eradicating ....." or line 421: "However, given the superiority of the results obtained with Lenti-HPV...". There are previous works on therapeutic vaccinations using E7

containing fusion proteins with Hsp65 (Chu NR, et al. Cell Stress Chaperones. 2000. PMID: 11189443), EDA fusion proteins (Arribillaga et al, J Immunother Cancer. 2020 PMID: 32581060), peptides (Domingos-Pereira J Immunother Cancer 2019 PMID: 31060612), or non replicating mRNA-LNP vaccines (Ramos da Silva, Sci Transl Med. 2023. PMID: 36867683) among many others, showing therapeutic efficiencies reaching 100% cures in similar tumor models. In the same line, as it is discussed in the "limitations of the study section", preclinical models do not adequately recapitulate or anticipate the results obtained in clinical trials.
In this study, the tumor model used is based on the administration of TC1 cells (s.c. or i.v in some settings). There are other more aggressive models, such as the one based on the administration of TC-1 P3 (A15) cells, with low expression of MHC-I, or the orthotopic tumor models (used in some of the above indicated publications) that could also allow a more in-depth analysis of the efficacy of the vaccine. This fact should also be mentioned among the limitations of the study.
2. Figure 3B. It is interesting to see the significant reduction in the numbers of CD4 T cells. Do you have an explanation for this finding? What about the Treg/CD4 ratio?
3. There is also a remarkable increase in the number of NK cells (Fig 4A). What is the therapeutic role of these cells in the model?
4. Figure 5A. The TME from Lenti-HPV-07-vaccinated mice showed stronger inflammatory signature than that observed for Ctrl Lenti-treatment. However there is also an upregulation of immunosuppressive cytokines such as IL10 or TGF-b. The authors could also comment this finding.
5. It is interesting to see that simultaneous administration of the vaccine and anti-PD1 has a detrimental effect. Please, revise figure S7 (In the text (line 284) it is indicated that 5 out of 10 mice treated with Lenti HPV-07 survived (50%), while in the figure it seems that this percentage is around 40%). Is this decline significant? This could be in agreement with previous works claiming a positive role for PD-1 expression (Odorizzi, P. M., Pauken, K. E., Paley, M. A., Sharpe, A. & Wherry, E. J. Genetic absence of PD-1 promotes accumulation of terminally differentiated exhausted CD8+ T cells. J Exp Med 212, 1125-1137 (2015)) PMID: 26034050
6. In the same line, the authors talk about synergy for the combination of the vaccine with anti-PD1 when it is administered later. Please, consider a statistical analysis to support the use of the term "synergy".
As a summary, the article is interesting and is in line with previous LV-based vaccination studies (Grasso et al 2013). Important work has been carried out on the characterization of the tumor microenvironment, which is of great value.

Referee #2 (Comments on Novelty/Model System for Author):

Well characterized tumor model.

Referee #2 (Remarks for Author):

Review of a full eradication of pre-clinal Human Papillomavirus virus induced tumor by lentiviral vaccine.

In this report, the authors describe a novel lentiviral based vaccine targeting the E6 and E7 proteins of HPV 16 & 18. They provide a description of the lentiviral vaccines they have constructed and demonstrate profound anti-tumor effects from implanted tumors that are subcutaneous.

These experiments are done in BLK/6 animals with subcutaneously injected models of HPV associated tumors. These data are impressive as a tumor regression is documented, with 100% of tumors treated with Lenti-HPV-07.

The analysis of these tumors includes analysis of the antigens specific T-cell responses. Most significantly is the figure 2a in which the lenti-HPV07 immune response appear to be prominently targeting E7 HPV16. The supplemental data suggests that the other vectors also induce similar levels of E7 HPV16 specific T cell responses.

The lenti HPV07 appears to express E7HPV16 as its primary sequence followed by E6HPV16, E7HPV18, and E6HPV18 . The other vectors have different configurations E7HPV16, E6HPV16, E7HPV18, and E6HPV18 and these other vectors appear to have less profound impact on the tumors that were implanted.

Major concern is the need for a more detailed analysis of the T cell responses to the vaccine is needed and correlation with the antitumor effects.

For example, what is the E6-HPV16, E6-HPV18, E7-HPV16, and E7HPV18 T cell response to the candidate vaccines Lenti-HPV-007, Lenti-HPV-008, Lenti-HPV-009, Lenti-HPV-0010?

Why is there a high T cell response to E7-HPV16, but a much reduced response to E6-HPV18, and E7HPV18 as shown in the supplemental data.

What is the protein expression of the E6-HPV16, E6-HPV18, E7-HPV16, and E7HPV18 T in the candidate vaccines Lenti-HPV-007, Lenti-HPV-008, Lenti-HPV-009, Lenti-HPV-0010?

Is the protein expression correlated to immune response?

Based on the higher response to E7-HPV16 relative to the E6 and E7 HPV18 response have biologic consequences? For example, does vaccine with E7-HPV16 alone provide protective immunity? Does vaccine without E7-HPV16 provide protective immunity?

The remaining portions of the manuscript report on characterization of the immune response from the potential effector cells and the combination of vaccines plus in the checkpoint blockade demonstrating a superior anti-tumor effects which is expected.

The addition of the immune checkpoint improves the response rate and the survival. It is surprising that the profound antitumor response shown in Figure 1 could not be reproduced in Figure 8 with a sub-optimal vaccine does. It would be useful to show that increasing tumor burden, either a higher injection or more advanced disease, could be cured with optimized vaccine and immune checkpoint blockade.

Finally, the metastatic model is confounded by the addition of the luciferase gene and selection markers which can induce immune responses and contribute to the antitumor response. This should be addressed with a model of metastasis without these additional antigens.

Point to point answers to reviewers' comments

***** Reviewer's comments *****

Reviewer #1 (Remarks for Author):

In this manuscript, Douguet et al studies the therapeutic efficacy of a non-integrative lentiviral vector encoding E6 and E7 oncoproteins of HPV16 and 18 genotypes (Lenti-HPV-07). They demonstrate that a single i.m. administration of the virus induced full eradication of HPV-induced tumors in 100% of mice. The authors have characterized in depth the phenotype and function of the generated T cells and found a profound remodeling of the tumor microenvironment towards an inflammatory state, with an important increase in CD8 T cell infiltration. The therapeutic vaccine significantly reduced lung metastases and induced a long lasting immune response. A synergistic effect with anti-PD1 therapy is also claimed. The article is well written and organized, and the results support the conclusions of the work. There are some issues that should be addressed before its acceptance for publication.

We are grateful to the Reviewer #1. Her/his remarks and questions contributed to widely improve the manuscript. We also performed additional in vivo experiments to better address the questions raised.

1. The first is a formal aspect. Authors should avoid expressions indicating a superiority that has not been completely verified, such as those expressed in lines 377 "The only immunotherapy described as effective in eradicating ....." or line 421: "However, given the superiority of the results obtained with Lenti-HPV...". There are previous works on therapeutic vaccinations using E7 containing fusion proteins with Hsp65 (Chu NR, et al. Cell Stress Chaperones. 2000. PMID: 11189443), EDA fusion proteins (Arribillaga et al, J Immunother Cancer. 2020 PMID: 32581060), peptides (Domingos-Pereira J Immunother Cancer 2019 PMID: 31060612), or non replicating mRNA-LNP vaccines (Ramos da Silva, Sci Transl Med. 2023. PMID: 36867683) among many others, showing therapeutic efficiencies reaching 100% cures in similar tumor models.

Discussion, Page 16, Lines: 443-446: We reworded this sentence and cited the references indicated by the Reviewer #1:

"Effective immunotherapies previously described against HPV-induced tumors in preclinical studies (Arribillaga *et al*, 2020; Chu *et al*, 2000; Domingos-Pereira *et al*, 2019; Grasso *et al.*, 2013; Ramos da Silva *et al*, 2023) include notably one that is based on an LV encoding a single HPV antigen (Grasso *et al.*, 2013). …"

In the same line, as it is discussed in the "limitations of the study section", preclinical models do not adequately recapitulate or anticipate the results obtained in clinical trials.

In this study, the tumor model used is based on the administration of TC1 cells (s.c. or i.v in some settings). There are other more aggressive models, such as the one based on the administration of TC-1 P3 (A15) cells, with low expression of MHC-I, or the orthotopic tumor models (used in some of the above indicated publications) that could also allow a more in-depth analysis of the efficacy of the vaccine. This fact should also be mentioned among the limitations of the study.

To answer this question by experimental results, we evaluated the efficacy of a single injection of Lenti-HPV-07 in mice bearing large TC-1 tumors:

Results, Pages 6-7, Lines: 134-142: "To evaluate the Lenti-HPV-07 efficacy in a more aggressive model, we treated mice bearing tumors with an average volume of 450 mm^3 with a single i.m. injection of 1×10^9 TU of Lenti-HPV-07 or Ctrl Lenti (Fig 1D-E). Six of 7 Lenti-HPV-07-treated mice were able to fully eradicate such large tumors or to induce significant decrease in tumor growth (Fig 1D). One mouse with the largest tumor in the Lenti-HPV-07 group had to be killed at day 28 post tumor implantation because of ethic consideration, yet we cannot exclude that this individual would have been able to control the tumor growth. Accordingly, the survival of Lenti-HPV-07-treated mice was significantly increased compared to

the Ctrl Lenti group (Fig 1E). Therefore, Lenti-HPV-07 induced full tumor regression in all animals that were ethically able to be maintained alive because of the large tumor size."

The legend to the Fig 1 has been adapted accordingly.

Discussion, Limitations of the study section: Page 18, Lines: 501-531: "There are other more aggressive models, such as low MHC-I expressing TC-1 P3 (A15) cells (Cheng *et al*, 2003), not tested in the present study. However, we showed the high efficacy of a single injection of Lenti-HPV-07 in the alternative aggressive large TC-1 tumor model, i.e., mice bearing tumors with an average volume of 450 mm^3."

2. Figure 3B. It is interesting to see the significant reduction in the numbers of CD4 T cells. Do you have an explanation for this finding? What about the Treg/CD4 ratio?

Results, Page 8, Lines: 189-196: We reworded this sentence and better cited the relevant figures so that they are clearer and more explanatory.

"Characterization of the intra-tumoral infiltrates at day 11 post-vaccination, i.e., during the tumor regression phase, showed: (i) a large increase in the percentage of $CD45^+$ hematopoietic cells (Fig 3A), and (ii) a significant increase in the percentages of $CD8^+$ T cells within the $CD45^+$ population, and (iii) a significant decrease in the percentages of $CD4^+$ T cells within the $CD45^+$ population (Fig 3B). The decrease in the $CD4^+$ T-cell proportions most likely resulted from the increased $CD8^+$ T cell proportions (Fig 3B) and/or reduction in the proportions of $CD25^+$ $FoxP3^+$ regulatory T cells (Tregs) within the $CD4^+$ T subset (Appendix Fig S3A)."

The percentages of intra-tumoral Treg vs $CD4^+$ T cells had been studied and showed in the present Fig S2A right, which is now better cited in the text, as mentioned above. The percentages of Treg vs $CD4^+$ T cells were largely significantly decreased in the Lenti-HPV-07-vaccinated mice, compared to the control group.

3. There is also a remarkable increase in the number of NK cells (Fig 4A). What is the therapeutic role of these cells in the model?

To address this question, we performed an in vivo experiment with TC-1 tumor-bearing mice, vaccinated with Lenti-HPV-07 and treated with the anti-NK depleting monoclonal antibody.

Results, Page 9, Lines: 220-221: "However, an efficient NK-cell depletion by anti-NK1.1 mAb treatment in tumor-bearing and Lenti-HPV-07-treated mice had no effect on the immune control of tumor growth (Fig EV4A-C)."

Discussion, Page 15, Lines: 414-417: "In the present study, depletion of NK cells revealed no role for these cells in regressing the growth of TC-1 cells, which constitutively express MHC-I molecules. However, it cannot be ruled out that in a clinical setting where tumor cells are more prone to lose their MHC-I expression, such NK recruitment could contribute to anti-tumor immunity."

4. Figure 5A. The TME from Lenti-HPV-07-vaccinated mice showed stronger inflammatory signature than that observed for Ctrl Lenti treatment. However, there is also an upregulation of immunosuppressive cytokines such as IL10 or TGF-b. The authors could also comment this finding.

Results, Page 10, Lines: 243-246: To mention and comment this observation, we added: "The immunosuppressive transforming growth factor (TGF)-β and IL-10 were also upregulated in the TME from Lenti-HPV-07-vaccinated mice, most likely as part of a typical attempt of the immune system prone to resolve inflammation and limit inflammation-induced tissue damage (Sanjabi *et al*, 2009)."

5. It is interesting to see that simultaneous administration of the vaccine and anti-PD1 has a detrimental effect. Please, revise figure S7 (In the text (line 284) it is indicated that 5 out of 10 mice treated with Lenti HPV-07 survived (50%), while in the figure it seems that this percentage is around 40%).
We thank the reviewer. In fact, one treated mouse had been forgotten by mistake in the survival curve. This individual is now reintroduced in the revised Fig EV5 C.

Is this decline significant?
Legend to the Fig EV5, Page 29, lines: 875-877: "Note that there is a net tendency to decline in the survival of mice treated with Lenti-HPV-07 + anti-PD1 compared to the mice treated with Lenti-HPV-07 alone, even though this difference did not reach statistical significance."

This could be in agreement with previous works claiming a positive role for PD-1 expression (Odorizzi, P. M., Pauken, K. E., Paley, M. A., Sharpe, A. & Wherry, E. J. Genetic absence of PD-1 promotes accumulation of terminally differentiated exhausted CD8+ T cells. J Exp Med 212, 1125-1137 (2015)) PMID: 26034050
Results, Page 15, Lines: 400-402: "This observation is also in agreement with previous results demonstrating a positive role for PD-1 in T-cell effector functions and decreased survival of $CD8^+$ T cells in the absence of PD-1 (Odorizzi *et al*, 2015)."

6. In the same line, the authors talk about synergy for the combination of the vaccine with anti-PD1 when it is administered later. Please, consider a statistical analysis to support the use of the term "synergy".
It can be seen, for instance in the Figure 8D, that the mean % of mice with anti-tumor response:

- Ctrl Lenti + anti-PD1 (10%)
- Lenti-HPV-07 + Ctrl Ig (25%)
- Lenti-HPV-07 + anti-PD1 (63.8%)

that 63.8% is much higher that the addition of 10% + 25%

Results, Pages 12-13, Lines: 345-348: "Because therapy with anti-PD1 mAb alone had practically no effect on tumor eradication and the impact of combination therapy was higher than that of Lenti-HPV-07 alone (Fig 8B, D, E), the effect of combination therapy was synergistic rather than additive."

As a summary, the article is interesting and is in line with previous LV-based vaccination studies (Grasso et al 2013). Important work has been carried out on the characterization of the tumor microenvironment, which is of great value.
We again thank the Reviewer #1 for her/his thorough analysis of the manuscript and for insightful questions.

Reviewer #2 (Remarks for Author):
Review of a full eradication of pre-clinal Human Papillomavirus virus induced tumor by lentiviral vaccine.

In this report, the authors describe a novel lentiviral based vaccine targeting the E6 and E7 proteins of HPV 16 & 18. They provide a description of the lentiviral vaccines they have constructed and demonstrate profound anti-tumor effects from implanted tumors that are subcutaneous.

These experiments are done in BLK/6 animals with subcutaneously injected models of HPV associated tumors. These data are impressive as a tumor regression is documented, with 100% of tumors treated with Lenti-HPV-07.

The analysis of these tumors includes analysis of the antigens specific T-cell responses. Most significantly is the figure 2a in which the lenti-HPV07 immune response appear to be prominently targeting E7 HPV16. The supplemental data suggests that the other vectors also induce similar levels of E7 HPV16 specific T cell responses.
The Lenti HPV-07 appears to express E7HPV16 as its primary sequence followed by E6HPV16, E7HPV18, and E6HPV18. The other vectors have different configurations E7HPV16, E6HPV16, E7HPV18, and E6HPV18 and these other vectors appear to have less profound impact on the tumors that were implanted.
We are grateful to the Reviewer #2. Her/his remarks let us know that some elements of the manuscript were not explicitly clear. Several points are now better described accordingly. We also performed additional in vivo experiments to better answer to the questions raised.

Major concern is the need for a more detailed analysis of the T cell responses to the vaccine is needed and correlation with the antitumor effects. For example, what is the E6-HPV16, E6-HPV18, E7-HPV16, and E7HPV18 T cell response to the candidate vaccines Lenti-HPV-07, Lenti-HPV-08, Lenti-HPV-09, Lenti-HPV-10?
Results, Page 7, Lines: 150-158: "We also comparatively analyzed IFN-γ T-cell responses against $E6_{HPV16}$, $E6_{HPV18}$, $E6_{HPV18}$ and $E7_{HPV18}$ antigens after immunization of C57BL/6 mice by Lenti-HPV-07, Lenti-HPV-08, Lenti-HPV-09 or Lenti-HPV-10 vectors. The T-cell immunogenicity profiles of the four Lenti-HPVs was very similar (Fig EV2A), consistent with their notable antitumor potential (Fig 1). Indeed, the slight differences between the antitumor activity of the four vectors (Fig 1) may be originated from a few outliers without significant impact on the tumor eradication rate or percentages of survival (Fig 1B-C). Importantly, the comparable immunogenicity of the four Lenti-HPVs against the selected HPV antigens clearly showed the lack of antigen positioning effect on T-cell immunogenicity of antigens in various Lenti-HPVs."

Why is there a high T cell response to E7-HPV16, but a much reduced response to E6-HPV18, and E7HPV18 as shown in the supplemental data.
Appendix Fig S2: In C57BL/6 (H-2^b) mice, there is the well described RAHYNIVTF H-$2D^b$-restricted immunodominant T-cell epitope in $E7_{HPV16}$, which explains the strong T-cell response induced. It appears that $E6_{HPV18}$ and $E7_{HPV18}$ do not contain such immunodominant epitopes for the H-2^b haplotype. These two antigens were still immunogenic in H-2^b mice, albeit at a lesser extent.

Appendix Fig S2: We preferred to reset the Y-axis scale to the same maximum value for all plots in this figure to make the level of response to each antigen more easily comparable visually.

Regardless of our response to this question of the reviewer, we realized that there was an error in the left graph of this figure, concerning the immunogenicity of $E6_{HPV16}$. With apologies, we have corrected these results. The immunogenicity of $E6_{HPV16}$ is very weak in C57BL/6 (H-2^b) mice, in accordance with all the other results in this manuscript.

What is the protein expression of the E6-HPV16, E6-HPV18, E7-HPV16, and E7HPV18 T in the candidate vaccines Lenti-HPV-007, Lenti-HPV-008, Lenti-HPV-009, Lenti-HPV-0010?

Discussion, Page 17, Lines: 457-464: "One might wonder whether the protein expression of the selected HPV antigens in the host cells transduced by Lenti-HPV-07, Lenti-HPV-08, Lenti-HPV-09 or Lenti-HPV-10 is comparable. It is technically challenging to set up a Western Blot to detect and quantitate the expression level of each of the four HPV antigens in host cells transduced with each of the Lenti-HPVs. In fact, there is no guarantee that the selected antigenic segments in the context of the poly-antigens are recognizable by mAbs which should be specific to sequential - and not conformational - B cell epitopes. However, the

comparable T-cell immunogenicity of the four Lenti-HPVs reflects well the comparable antigen availabilities in host cells transduced by each of these Lenti-HPV vectors."

Results Pages 7-8, Lines: 172-179: "Altogether, the immunogenicity of Lenti-HPV-07: (i) against $E7_{HPV16}$ and also at a lesser extent against $E6_{HPV18}$ and $E7_{HPV18}$ in C57BL/6 (H-2^b) mice (Fig 2A), and (ii) against $E6_{HPV18}$ in MHC-humanized HHD-DR1 mice (Fig 2E), reflected well the accurate expression of these various antigens in the host antigen presenting cells transduced by Lenti-HPV-07. We also emphasize that the four antigens are expressed as a single polyprotein, and that the four segments can only be expressed with the same intensity. The differences in the profile of Lenti-HPV-07 immunogenicity in these distinct animal models is linked to the selection of different T-cell epitopes by diverse MHC molecules from various H-2 or HLA haplotypes for presentation to T cells."

Based on the higher response to E7-HPV16 relative to the E6 and E7 HPV18 response have biologic consequences?
For example, does vaccine with E7-HPV16 alone provide protective immunity? Does vaccine without E7-HPV16 provide protective immunity?
Discussion, Page 17, Lines: 465-474: "Based on data from the literature, the immunogenicity of $E7_{HPV16}$ in H-2^b mice is most likely responsible for the overall anti-TC-1 tumor response in the C57BL/6 mice (Grasso *et al.*, 2013). This antigen alone encoded by viral vectors or other vaccination strategies is the one which achieves the protection against HPV-induced TC-1 tumors. The interest of the inclusion of a poly-antigen in Lenti-HPV-07 was not to broaden more the immunogenicity of the vaccine in the C57BL/6 model. The response of C57BL/6 mice to $E7_{HPV16}$ in this model validates the proof of concept. The interest of adding more antigens $E6_{HPV16}$, $E6_{HPV18}$ and $E7_{HPV18}$ antigens was essentially to broaden the antigen/epitope repertoire with a short-term perspective of achieving a clinical trial in patients, with polymorphic HLA, infected by HPV16 or HPV18. It is also noteworthy that patients with HPV16 and HPV18 co-infection have been reported (Jesus *et al*, 2018)."

The remaining portions of the manuscript report on characterization of the immune response from the potential effector cells and the combination of vaccines plus in the checkpoint blockade demonstrating a superior anti-tumor effects which is expected.
The addition of the immune checkpoint improves the response rate and the survival. It is surprising that the profound antitumor response shown in Figure 1 could not be reproduced in Figure 8 with a sub-optimal vaccine does.
Results, Page 12, Lines: 326-329: "Since the dose of 1×10^9 TU, as used in previous experiments, induced complete tumor eradication in 100% of the mice (Fig 1), to evaluate the possible beneficial effect of the additional anti-PD1 mAb treatment, we used the suboptimal dose of 1×10^8 TU, which is far from being effective alone."

Results, Page 12, Lines: 336-338: In addition, to make the sentence clearer, we reworded the following statement:
"In this protocol, the suboptimal dose of 1×10^8 TU of Lenti-HPV-07, which induced an insufficient antitumor T-cell response, acted synergistically with anti-PD1 therapy to increase the tumor regression rate".

It would be useful to show that increasing tumor burden, either a higher injection or more advanced disease, could be cured with optimized vaccine and immune checkpoint blockade.

Results, Page 13, Lines: 349-355: "In contrast to the high efficacy of the optimal dose of 1×10^9 TU of Lenti-HPV-07 on very large tumors with an average volume of 450 mm^3 (Fig 1D-E), the combination of a

suboptimal dose of 1×10^8 TU of Lenti-HPV-07 with anti-PD1 mAb therapy was not effective on very large tumors (Appendix Fig S3).

Therefore, a synergistic anti-tumor effect can be achieved when Lenti-HPV-07 is combined with delayed anti-PD1 checkpoint inhibitory treatment only in the case of small- or medium-sized tumors. Optimal dose of Lenti-HPV-07 alone remains the best therapeutic option for large tumors."

Finally, the metastatic model is confounded by the addition of the luciferase gene and selection markers which can induce immune responses and contribute to the antitumor response. This should be addressed with a model of metastasis without these additional antigens.

Our initial attempt to follow the TC-1 tumor metastasis to the lungs by histology failed, most seemingly due to the weak sensitivity of the method resulting from the fact that histological sections cannot cover the totality of the organs. In addition, this method would have required the killing of 7-10 animals/group/time point. Moreover, due to its low sensitivity, the histological method would have required injection of a much larger number of tumor cells per animal, further deviating from the conditions we attempt to maintain as close as possible to genuine metastasis.

Cytometric detection would have been a sensitive alternative method but would have again required to kill several animals per time point and also to introduce a fluorochrome marker to TC-1 cells, which would have generated the same questions about the possible immunogenicity of the reporter. On the other hand, without an internal reporter signal, TC-1 cells are hardly stainable for analysis by cytometry because of the lack of specific surface makers.

For these reasons, we opted for bioluminescence-based live animal imaging which is one of the most accurate, sensitive and quantitative assessment of tumor burden over time and allowing longitudinal tracking of metastases in individual animals.

To the best of our knowledge, there are no data in the literature showing any sign of immunogenicity of the very small (10 kDa) nLuc enzyme originated from *Oplophorus gracilirostris* and, in fact, in our hands, nLuc behaves as a non-immunogenic protein. Indeed, when a lentiviral vector encoding nLuc alone (without any other antigenic protein) is injected i.m. directly to C57BL/6 mice, there is no immunity and the reporter signal remains largely detectable at the injection site for at least 2-3 weeks. In net opposite, while a lentiviral vector encoding nLuc and another immunogenic protein is injected i.m. directly to C57BL/6 mice, the immunogenicity of the antigen accelerates the clearance of the vector, which disappear in 11-12 days. This observation strongly suggests that nLuc is not immunogenic per se, at least in C57BL/6 mice.

We do not have indication on the immunogenicity of puromycin N-acetyl-transferase which has been used to select the transduced nLuc TC-1 cells. However, we used the same TC-1-nLuc cells to inject i.v. to the mice in which we detected the lung metastases and which received Ctrl Lenti or Lenti-HPV-07.

Results, Page 10, Lines: 265-277: "We generated a TC-1 cell line stably expressing the nano-luciferase reporter gene (nLuc-TC-1) to apply a bioluminescence-based live animal imaging which is one of the most sensitive and quantitative assessment of tumor burden over time and which allows longitudinal tracking of metastases in individual animals. Such an analysis by histological method would have weak sensitivity as histological sections cannot cover the totality of the organs and would have required injection of a much larger number of tumor cells per animal. Cytometric detection would have been a sensitive alternative method but would have required introduction of a fluorochrome reporter to TC-1 cells, which would have generated the same questions about the possible immunogenicity of the reporter. To the best of our knowledge, there are no data in the literature showing any sign of immunogenicity of the small nLuc (10 kDa) enzyme which behaves as a non-immunogenic protein in our hands. In any case, we

compared the extent of metastasis with the same nLuc-TC-1 cell line in groups of mice treated with Ctrl Lenti or Lenti-HPV-07 in which a possible immunogenicity of the reporter would be the same and could not be a confounding factor."

In addition to the answers to the reviewers, we also added a few lines in the Discussion since during the revision of this manuscript, a study on mRNA-based immuno-oncotherapy of HPV-induced tumors has been published in *Science Translational Medicine*. To include this information and to compare the efficacy of this strategy to that of Lenti-HPV-07, we added:

Discussion, Page 16, Lines: 439-446: "In a recent study using mRNA technology, including its auto-amplifying version which prolongs antigen expression in the vaccinated mice, the immunotherapeutic potential of this technology could only be demonstrated against TC-1 tumors, either not yet established or of very small size, with early relapses in almost 50% of treated mice (Ramos da Silva *et al*, 2023)."

14th Aug 2023

Dear Dr. Majlessi,

Thank you for the submission of your revised manuscript to EMBO Molecular Medicine. I am pleased to inform you that we will be able to accept your manuscript pending the following final amendments:

1) In the main manuscript file, please do the following:
- Correct/answer the track changes suggested by our data editors by working from the attached document.
- Order of sections should be as followed: abstract, introduction, results, discussion, materials & methods, data availability section, acknowledgments, disclosure statement and competing interests, references, main figure legends, tables, expanded figure legends.
- Remove font color.
- Please make sure thar figure callouts are in a sequential order. Currently Fig 8D is called out before Fig 8C. Also, callout for Appendix Fig. S4 is missing. Please correct.
- In M&M, statistical paragraph should reflect all information that you have filled in the Authors Checklist, especially regarding randomization, blinding, replication.
- Please rename "Conflict of Interest" to "Disclosure Statement & Competing Interests". We updated our journal's competing interests policy in January 2022 and request authors to consider both actual and perceived competing interests. Please review the policy https://www.embopress.org/competing-interests and update your competing interests if necessary.
- Data availability: Availability of deposited data only should be reported. Please remove the password and link to the private page,
- Correct the reference citation in the reference list. Where there are more than 10 authors on a paper, 10 will be listed, followed by "et al.". Please check "Author Guidelines" for more information.
https://www.embopress.org/page/journal/17574684/authorguide#referencesformat
2) Funding: Please make sure that information about all sources of funding are complete in both our submission system and in the manuscript. Currently Grant Reference Number missing in the ms file: Lenti-HPV-07 is missing in our submission system.
3) Appendix: Please rename the figures and the table in the table of contents to Appendix Figure S1 etc. and Appendix Table S1 and add page numbers. Please check the nomenclature of the callouts in the manuscript text as well.
4) Synopsis:
- Synopsis image: Please provide the visual abstract as a high-resolution jpeg file 550 px-wide x (250-400)-px high.
- Synopsis text: Please remove it from the main manuscript text and upload it as a separate file.
- Please check your synopsis text and image before submission with your revised manuscript. Please be aware that in the proof stage minor corrections only are allowed (e.g., typos).
5) For more information: This space should be used to list relevant web links for further consultation by our readers. Could you identify some relevant ones and provide such information as well? Some examples are patient associations, relevant databases, OMIM/proteins/genes links, author's websites, etc...
6) Source data: Please upload source data as one (zipped) file per figure, i.e. all the source data files related to a single figure should be zipped up into one zip file; please provide all figure source data with your revised manuscript - it appears that the following are missing: 2B, 3C, 6A, 6D, 7E.
7) As part of the EMBO Publications transparent editorial process initiative (see our Editorial at http://embomolmed.embopress.org/content/2/9/329), EMBO Molecular Medicine will publish online a Review Process File (RPF) to accompany accepted manuscripts. This file will be published in conjunction with your paper and will include the anonymous referee reports, your point-by-point response and all pertinent correspondence relating to the manuscript. Let us know whether you agree with the publication of the RPF and as here, if you want to remove or not any figures from it prior to publication. Please note that the Authors checklist will be published at the end of the RPF.
8) Please provide a point-by-point letter INCLUDING my comments as well as the reviewer's reports and your detailed responses (as Word file).

I look forward to reading a new revised version of your manuscript as soon as possible.

Yours sincerely,

Zeljko Durdevic

Zeljko Durdevic
Editor
EMBO Molecular Medicine

*** Instructions to submit your revised manuscript ***

*** PLEASE NOTE *** As part of the EMBO Publications transparent editorial process initiative (see our Editorial at https://www.embopress.org/doi/pdf/10.1002/emmm.201000094), EMBO Molecular Medicine will publish online a Review Process File to accompany accepted manuscripts.

In the event of acceptance, this file will be published in conjunction with your paper and will include the anonymous referee reports, your point-by-point response and all pertinent correspondence relating to the manuscript. If you do NOT want this file to be published, please inform the editorial office at contact@embomolmed.org.

When submitting your revised manuscript, please include:

1) a .docx formatted version of the manuscript text (including Figure legends and tables)

2) Separate figure files*

3) supplemental information as Expanded View and/or Appendix. Please carefully check the authors guidelines for formatting Expanded view and Appendix figures and tables at
https://www.embopress.org/page/journal/17574684/authorguide#expandedview

4) a letter INCLUDING the reviewer's reports and your detailed responses to their comments (as Word file).

5) The paper explained: EMBO Molecular Medicine articles are accompanied by a summary of the articles to emphasize the major findings in the paper and their medical implications for the non-specialist reader. Please provide a draft summary of your article highlighting
- the medical issue you are addressing,
- the results obtained and
- their clinical impact.
This may be edited to ensure that readers understand the significance and context of the research.
Please refer to any of our published articles for an example.

6) For more information: There is space at the end of each article to list relevant web links for further consultation by our readers. Could you identify some relevant ones and provide such information as well? Some examples are patient associations, relevant databases, OMIM/proteins/genes links, author's websites, etc...

7) Author contributions: the contribution of every author must be detailed in a separate section.

8) EMBO Molecular Medicine now requires a complete author checklist (https://www.embopress.org/page/journal/17574684/authorguide) to be submitted with all revised manuscripts. Please use the checklist as guideline for the sort of information we need WITHIN the manuscript. The checklist should only be filled with page numbers were the information can be found. This is particularly important for animal reporting, antibody dilutions (missing) and exact values and n that should be indicted instead of a range.

9) Every published paper now includes a 'Synopsis' to further enhance discoverability. Synopses are displayed on the journal webpage and are freely accessible to all readers. They include a short stand first (maximum of 300 characters, including space) as well as 2-5 one sentence bullet points that summarise the paper. Please write the bullet points to summarise the key NEW findings. They should be designed to be complementary to the abstract - i.e. not repeat the same text. We encourage inclusion of key acronyms and quantitative information (maximum of 30 words / bullet point). Please use the passive voice. Please attach these in a separate file or send them by email, we will incorporate them accordingly.

You are also welcome to suggest a striking image or visual abstract to illustrate your article. If you do please provide a jpeg file 550 px-wide x 400-px high.

10) A Conflict of Interest statement should be provided in the main text

11) Please note that we now mandate that all corresponding authors list an ORCID digital identifier. This takes <90 seconds to

complete. We encourage all authors to supply an ORCID identifier, which will be linked to their name for unambiguous name identification.

Currently, our records indicate that the ORCID for your account is 0000-0001-8648-4058.

Please click the link below to modify this ORCID:
Link Not Available

12) The system will prompt you to fill in your funding and payment information. This will allow Wiley to send you a quote for the article processing charge (APC) in case of acceptance. This quote takes into account any reduction or fee waivers that you may be eligible for. Authors do not need to pay any fees before their manuscript is accepted and transferred to our publisher.

*Additional important information regarding Figures

Each figure should be given in a separate file and should have the following resolution:
Graphs 800-1,200 DPI
Photos 400-800 DPI
Colour (only CMYK) 300-400 DPI"

Figures are not edited by the production team. All lettering should be the same size and style; figure panels should be indicated by capital letters (A, B, C etc). Gridlines are not allowed except for log plots. Figures should be numbered in the order of their appearance in the text with Arabic numerals. Each Figure must have a separate legend and a caption is needed for each panel.

*Additional important information regarding figures and illustrations can be found at https://bit.ly/EMBOPressFigurePreparationGuideline. See also figure legend preparation guidelines: https://www.embopress.org/page/journal/17574684/authorguide#figureformat

The system will prompt you to fill in your funding and payment information. This will allow Wiley to send you a quote for the article processing charge (APC) in case of acceptance. This quote takes into account any reduction or fee waivers that you may be eligible for. Authors do not need to pay any fees before their manuscript is accepted and transferred to our publisher.

***** Reviewer's comments *****

Referee #1 (Remarks for Author):

The authors carried out an effort and performed new experiments. They have addressed all my comments successfully.

Point to point, Manuscript #EMM-2023-17723

1) In the main manuscript file, please do the following:
- Correct/answer the track changes suggested by our data editors by working from the attached document.
We corrected/answered the track changes asked by data editors in the QC document.

- Order of sections should be as followed: abstract, introduction, results, discussion, materials & methods, data availability section, acknowledgments, disclosure statement and competing interests, references, main figure legends, tables, expanded figure legends.
We reordered the sections as requested.

- Remove font color.
We removed font color.

- Please make sure that figure callouts are in a sequential order. Currently Fig 8D is called out before Fig 8C. Also, callout for Appendix Fig. S4 is missing. Please correct.
On Figure 8, we reversed the position of Figures 8 C and D, so that they now appear in the right order in the text. We have also adapted the legend to the Figure 8 accordingly.

- In M&M, statistical paragraph should reflect all information that you have filled in the Authors Checklist, especially regarding randomization, blinding, replication.

We completed the statistical paragraph as following: "*Mice were randomized on the day of immunization. Mice were allocated to cages so that each cage contained individuals representing each treatment.*"

- Please rename "Conflict of Interest" to "Disclosure Statement & Competing Interests".
We updated our journal's competing interests policy in January 2022 and request authors to consider both actual and perceived competing interests. Please review the policy https://www.embopress.org/competing-interests and update your competing interests if necessary.

We renamed this paragraph as requested.

We completed the Disclosure Statement & Competing Interests paragraph as following: "*LD, IF, BV, FLC, FM, PA, TN, AN, FN, FA, LM and PC declare competing financial interests related to the publication of this study.*"

- Data availability: Availability of deposited data only should be reported. Please remove the password and link to the private page,
We removed the password and linked them to the repository data bank.

- Correct the reference citation in the reference list. Where there are more than 10 authors on a paper, 10 will be listed, followed by "et al.". Please check "Author Guidelines" for more information.
https://www.embopress.org/page/journal/17574684/authorguide#referencesformat
Please note that the references had been formatted as such.

2) Funding: Please make sure that information about all sources of funding are complete in both our submission system and in the manuscript. Currently Grant Reference Number missing in the ms file: Lenti-HPV-07 is missing in our submission system.
Please note that there is no grant reference number for TheraVectys and that no specific - but only general - grant has been allocated by Institut Pasteur, without particular grant reference number.

3) Appendix: Please rename the figures and the table in the table of contents to Appendix Figure S1 etc. and Appendix Table S1 and add page numbers.
The supplemental Figures and Table are now renamed in Appendix and in the main text, as requested. Page numbers were also added to Appendix.

Please check the nomenclature of the callouts in the manuscript text as well.
the callouts Appendix Table S1 in the manuscript.
They were checked and corrected when necessary.

4) Synopsis:
- Synopsis image: Please provide the visual abstract as a high-resolution jpeg file 550 px-wide x (250-400)-px high.
Synopsis image provided.

- Synopsis text: Please remove it from the main manuscript text and upload it as a separate file.
Synopsis text was removed from the main text and was uploaded as a separate file.

- Please check your synopsis text and image before submission with your revised manuscript. Please be aware that in the proof stage minor corrections only are allowed (e.g., typos).
We checked the synopsis text and image.

5) For more information: This space should be used to list relevant web links for further consultation by our readers. Could you identify some relevant ones and provide such information as well? Some examples are patient associations, relevant databases, OMIM/proteins/genes links, author's websites, etc...
We added our website (*https://theravectys.site/*) above References section "For more information"

6) Source data: Please upload source data as one (zipped) file per figure, i.e. all the source data files related to a single figure should be zipped up into one zip file;
Regarding the source data files, we proceeded as requested and uploaded zipped files per figure.

Please provide all figure source data with your revised manuscript - it appears that the following are missing: 2B, 6A, 6D, 7E.
Figure source data have been provided each time it was possible.
Fig 2B, 6D, 7E are cytometric images, representative of the data. The cytometric raw data used to generate them are provided in Repository data. The digital results they represent are provided in 2C-D, 6B-C, and 7F, respectively.

Fig 3C is a histological image. A full set of histological data are provided in EV3.

Fig 6A is a Small Animal Imaging result. The digital results they represent are provided in 6B-C.

7) As part of the EMBO Publications transparent editorial process initiative (see our Editorial at http://embomolmed.embopress.org/content/2/9/329), EMBO Molecular Medicine will publish online a Review Process File (RPF) to accompany accepted manuscripts. This file will be published in conjunction with your paper and will include the anonymous referee reports, your point-by-point response and all pertinent correspondence relating to the manuscript. Let us know whether you agree with the publication of the RPF and as here, if you want to remove or not any figures from it prior to publication.
We fully agree with the publication of the Review Process File. We do not want to remove any element from it prior to publication.

Please note that the Authors checklist will be published at the end of the RPF.
We agree.

8) Please provide a point-by-point letter INCLUDING my comments as well as the reviewer's reports and your detailed responses (as Word file).
Here is the point-to-point letter, as requested.

***** Reviewer's comments *****

Referee #1 (Remarks for Author):

The authors carried out an effort and performed new experiments. They have addressed all my comments successfully.
Our thanks once again to the reviewers for their analysis and comments.

23rd Aug 2023

Dear Dr. Majlessi,

We are pleased to inform you that your manuscript is accepted for publication and is now being sent to our publisher to be included in the next available issue of EMBO Molecular Medicine.

Please read below for additional IMPORTANT information regarding your article, its publication and the production process.

Congratulations on your interesting work,
Zeljko Durdevic

Zeljko Durdevic
Editor
EMBO Molecular Medicine

Follow us on Twitter @EmboMolMed
Sign up for eTOCs at embopress.org/alertsfeeds

*** *** *** IMPORTANT INFORMATION *** *** ***

SPEED OF PUBLICATION
The journal aims for rapid publication of papers, using using the advance online publication "Early View" to expedite the process: A properly copy-edited and formatted version will be published as "Early View" after the proofs have been corrected. Please help the Editors and publisher avoid delays by providing e-mail address(es), telephone and fax numbers at which author(s) can be contacted.

Should you be planning a Press Release on your article, please get in contact with embomolmed@wiley.com as early as possible, in order to coordinate publication and release dates.

LICENSE AND PAYMENT:

All articles published in EMBO Molecular Medicine are fully open access: immediately and freely available to read, download and share.

EMBO Molecular Medicine charges an article processing charge (APC) to cover the publication costs. You, as the corresponding author for this manuscript, should have already received a quote with the article processing fee separately. Please let us know in case this quote has not been received.

Once your article is at Wiley for editorial production you will receive an email from Wiley's Author Services system, which will ask you to log in and will present you with the publication license form for completion. Within the same system the publication fee can be paid by credit card, an invoice, pro forma invoice or purchase order can be requested.

Payment of the publication charge and the signed Open Access Agreement form must be received before the article can be published online.

PROOFS

You will receive the proofs by e-mail approximately 2 weeks after all relevant files have been sent o our Production Office. Please return them within 48 hours and if there should be any problems, please contact the production office at embopressproduction@wiley.com.

Please inform us if there is likely to be any difficulty in reaching you at the above address at that time. Failure to meet our deadlines may result in a delay of publication.

All further communications concerning your paper proofs should quote reference number EMM-2023-17723-V3 and be directed to the production office at embopressproduction@wiley.com.

Thank you,

Zeljko Durdevic
Editor
EMBO Molecular Medicine

EMBO Press Author Checklist

Corresponding Author Name: Laleh MAJLESSI
Journal Submitted to: EMBO Molecular Medicine
Manuscript Number: EMM-2023-17723

USEFUL LINKS FOR COMPLETING THIS FORM
The EMBO Journal - Author Guidelines
EMBO Reports - Author Guidelines
Molecular Systems Biology - Author Guidelines
EMBO Molecular Medicine - Author Guidelines

Reporting Checklist for Life Science Articles (updated January
This checklist is adapted from Materials Design Analysis Reporting (MDAR) Checklist for Authors. MDAR establishes a minimum set of requirements in transparent reporting in the life sciences (see Statement of Task: 10.31222/osf.io/9sm4x). Please follow the journal's guidelines in preparing your manuscript.
Please note that a copy of this checklist will be published alongside your article.

Abridged guidelines for figures
1. Data
The data shown in figures should satisfy the following conditions:
- the data were obtained and processed according to the field's best practice and are presented to reflect the results of the experiments in an accurate and unbiased manner.
- ideally, figure panels should include only measurements that are directly comparable to each other and obtained with the same assay.
- plots include clearly labeled error bars for independent experiments and sample sizes. Unless justified, error bars should not be shown for technical
- if n<5, the individual data points from each experiment should be plotted. Any statistical test employed should be justified.
- Source Data should be included to report the data underlying figures according to the guidelines set out in the authorship guidelines on Data

2. Captions
Each figure caption should contain the following information, for each panel where they are relevant:
- a specification of the experimental system investigated (eg cell line, species name).
- the assay(s) and method(s) used to carry out the reported observations and measurements.
- an explicit mention of the biological and chemical entity(ies) that are being measured.
- an explicit mention of the biological and chemical entity(ies) that are altered/varied/perturbed in a controlled manner.
- the exact sample size (n) for each experimental group/condition, given as a number, not a range;
- a description of the sample collection allowing the reader to understand whether the samples represent technical or biological replicates (including how many animals, litters, cultures, etc.).
- a statement of how many times the experiment shown was independently replicated in the laboratory.
- definitions of statistical methods and measures:
  - common tests, such as t-test (please specify whether paired vs. unpaired), simple χ2 tests, Wilcoxon and Mann-Whitney tests, can be unambiguously identified by name only, but more complex techniques should be described in the methods section;
  - are tests one-sided or two-sided?
  - are there adjustments for multiple comparisons?
  - exact statistical test results, e.g., P values = x but not P values < x;
  - definition of 'center values' as median or average;
  - definition of error bars as s.d. or s.e.m.

Please complete ALL of the questions below.
Select "Not Applicable" only when the requested information is not relevant for your study.

Materials

Newly Created Materials	Information included in the manuscript?	In which section is the information available? (Reagents and Tools Table, Materials and Methods, Figures, Data Availability Section)
New materials and reagents need to be available; do any restrictions apply?	Yes	Data Availability Section

Antibodies	Information included in the manuscript?	In which section is the information available? (Reagents and Tools Table, Materials and Methods, Figures, Data Availability Section)
For **antibodies** provide the following information:<br>- Commercial antibodies: RRID (if possible) or supplier name, catalogue number and or/clone number<br>- Non-commercial: RRID or citation	Yes	Are antibodies used in this study have been purchased, are commercially available. Their supplier name and catalogue number/clone number are indicated in the Material & Methods Section.

DNA and RNA sequences	Information included in the manuscript?	In which section is the information available? (Reagents and Tools Table, Materials and Methods, Figures, Data Availability Section)
**Short novel DNA or RNA including primers, probes**: provide the sequences.	Yes	A.a. sequences of polyantigens inserted in the lentiviral vectors are included in the Figures and Supplemental Data. DNA sequence of these polyantigens are available in the Supplemental Data.

Cell materials	Information included in the manuscript?	In which section is the information available? (Reagents and Tools Table, Materials and Methods, Figures, Data Availability Section)
**Cell lines:** Provide species information, strain. Provide accession number in repository **OR** supplier name, catalog number, clone number, and/**OR** RRID.	Yes	The information on the TC-1 cell line are provided in the Material and Method Section.
**Primary cultures:** Provide species, strain, sex of origin, genetic modification status.	Yes	Results and Material and Method Sections.
Report if the cell lines were recently **authenticated** (e.g., by STR profiling) and tested for mycoplasma contamination.	Yes	TC-1 cell line have been authenticated and tested negative for mycoplasma contamination.

Experimental animals	Information included in the manuscript?	In which section is the information available? (Reagents and Tools Table, Materials and Methods, Figures, Data Availability Section)
**Laboratory animals or Model organisms:** Provide species, strain, sex, age, genetic modification status. Provide accession number in repository **OR** supplier name, catalog number, clone number, **OR** RRID.	Yes	Materials and Methods Section
**Animal observed in or captured from the field:** Provide species, sex, and age where possible.	Not Applicable	
Please detail **housing and husbandry conditions**.	Yes	Materials and Methods Section

Plants and microbes	Information included in the manuscript?	In which section is the information available? (Reagents and Tools Table, Materials and Methods, Figures, Data Availability Section)
**Plants:** provide species and strain, ecotype and cultivar where relevant, unique accession number if available, and source (including location for collected wild specimens).	Not Applicable	
**Microbes:** provide species and strain, unique accession number if available, and source.	Not Applicable	

Human research participants	Information included in the manuscript?	In which section is the information available? (Reagents and Tools Table, Materials and Methods, Figures, Data Availability Section)
If collected and within the bounds of privacy constraints report on age, sex and gender or ethnicity for all study participants.	Not Applicable	

Core facilities	Information included in the manuscript?	In which section is the information available? (Reagents and Tools Table, Materials and Methods, Figures, Data Availability Section)
If your work benefited from core facilities, was their service mentioned in the acknowledgments section?	Yes	Acknowledgments

Design

Study protocol	Information included in the manuscript?	In which section is the information available? (Reagents and Tools Table, Materials and Methods, Figures, Data Availability Section)
If study protocol has been **pre-registered, provide DOI in the manuscript**. For clinical trials, provide the trial registration number **OR** cite DOI.	Not Applicable	
Report the **clinical trial registration number** (at ClinicalTrials.gov or equivalent), where applicable.	Not Applicable	

Laboratory protocol	Information included in the manuscript?	In which section is the information available? (Reagents and Tools Table, Materials and Methods, Figures, Data Availability Section)
Provide DOI OR other citation details if **external detailed step-by-step protocols** are available.	Not Applicable	

Experimental study design and statistics	Information included in the manuscript?	In which section is the information available? (Reagents and Tools Table, Materials and Methods, Figures, Data Availability Section)
Include a statement about **sample size** estimate even if no statistical methods were used.	Yes	Figure Captions
Were any steps taken to minimize the effects of subjective bias when allocating animals/samples to treatment (e.g. **randomization procedure**)? If yes, have they been described?	Yes	Material and Methods section
Include a statement about **blinding** even if no blinding was done.	Not Applicable	
Describe **inclusion/exclusion criteria** if samples or animals were excluded from the analysis. Were the criteria pre-established?<br><br>If sample or data points were omitted from analysis, report if this was due to attrition or intentional exclusion and provide justification.	Not Applicable	
For every figure, are **statistical tests** justified as appropriate? Do the data meet the assumptions of the tests (e.g., normal distribution)? Describe any methods used to assess it. Is there an estimate of variation within each group of data? Is the variance similar between the groups that are being statistically compared?	Yes	The statistical tests are all indicated in the Figure Legends and their use justified.

Sample definition and in-laboratory replication	Information included in the manuscript?	In which section is the information available? (Reagents and Tools Table, Materials and Methods, Figures, Data Availability Section)
In the figure legends: state number of times the experiment was **replicated** in laboratory.	Yes	Done in the Figure Legends.
In the figure legends: define whether data describe **technical or biological replicates**.	Yes	Done in the Figure Legends.

Ethics

Ethics	Information included in the manuscript?	In which section is the information available? (Reagents and Tools Table, Materials and Methods, Figures, Data Availability Section)
Studies involving **human participants**: State details of **authority granting ethics approval** (IRB or equivalent committee(s), provide reference number for approval.	Not Applicable	
Studies involving **human participants**: Include a statement confirming that **informed consent** was obtained from all subjects and that the experiments conformed to the principles set out in the WMA Declaration of Helsinki and the Department of Health and Human Services Belmont Report.	Not Applicable	
Studies involving **human participants:** For publication of **patient photos**, include a statement confirming that consent to publish was obtained.	Not Applicable	
Studies involving experimental **animals**: State details of **authority granting ethics approva**l (IRB or equivalent committee(s), provide reference number for approval. Include a statement of compliance with ethical regulations.	Yes	Material and Methods Section.
Studies involving **specimen and field samples:** State if relevant **permits** obtained, provide details of authority approving study; if none were required, explain why.	Not Applicable	

Dual Use Research of Concern (DURC)	Information included in the manuscript?	In which section is the information available? (Reagents and Tools Table, Materials and Methods, Figures, Data Availability Section)
Could your study fall under dual use research restrictions? Please check biosecurity documents and list of **select agents and toxins** (CDC): https://www.selectagents.gov/sat/list.htm	Not Applicable	
If you used a select agent, is the security level of the lab appropriate and reported in the manuscript?	Not Applicable	
If a study is subject to dual use research of concern regulations, is the name of the **authority granting approval and reference number** for the regulatory approval provided in the manuscript?	Not Applicable	

Reporting

The MDAR framework recommends adoption of discipline-specific guidelines, established and endorsed through community initiatives. Journals have their own policy about requiring specific guidelines and recommendations to complement MDAR.

Adherence to community standards	Information included in the manuscript?	In which section is the information available? (Reagents and Tools Table, Materials and Methods, Figures, Data Availability Section)
State if relevant guidelines or checklists (e.g., **ICMJE, MIBBI, ARRIVE, PRISMA**) have been followed or provided.	Not Applicable	
For **tumor marker prognostic studies,** we recommend that you follow the **REMARK** reporting guidelines (see link list at top right). See author guidelines, under 'Reporting Guidelines'. Please confirm you have followed these guidelines.	Not Applicable	
For **phase II and III randomized controlled trials**, please refer to the **CONSORT** flow diagram (see link list at top right) and submit the CONSORT checklist (see link list at top right) with your submission. See author guidelines, under 'Reporting Guidelines'. Please confirm you have submitted this list.	Not Applicable	

Data Availability

Data availability	Information included in the manuscript?	In which section is the information available? (Reagents and Tools Table, Materials and Methods, Figures, Data Availability Section)
Have **primary datasets** been deposited according to the journal's guidelines (see 'Data Deposition' section) and the respective accession numbers provided in the Data Availability Section?	Yes	Material and Methods Section.
Were **human clinical and genomic datasets** deposited in a public access-controlled repository in accordance to ethical obligations to the patients and to the applicable consent agreement?	Not Applicable	
Are **computational models** that are central and integral to a study available without restrictions in a machine-readable form? Were the relevant accession numbers or links provided?	Not Applicable	
If publicly available data were reused, provide the respective **data citations in the reference list**.	Not Applicable	
